# Stimuli-sensitive polymer prodrug nanocarriers by reversible-deactivation radical polymerization

**DOI:** 10.1039/d2cs01060g

**Published:** 2024-05-22

**Authors:** Léa Guerassimoff, Marianne Ferrere, Amaury Bossion, Julien Nicolas

**Affiliations:** a Université Paris-Saclay, CNRS, Institut Galien Paris-Saclay 91400 Orsay France julien.nicolas@universite-paris-saclay.fr +33 1 80 00 60 81

## Abstract

Polymer prodrugs are based on the covalent linkage of therapeutic molecules to a polymer structure which avoids the problems and limitations commonly encountered with traditional drug-loaded nanocarriers in which drugs are just physically entrapped (*e.g.*, burst release, poor drug loadings). In the past few years, reversible-deactivation radical polymerization (RDRP) techniques have been extensively used to design tailor-made polymer prodrug nanocarriers. This synthesis strategy has received a lot of attention due to the possibility of fine tuning their structural parameters (*e.g.*, polymer nature and macromolecular characteristics, linker nature, physico-chemical properties, functionalization, *etc.*), to achieve optimized drug delivery and therapeutic efficacy. In particular, adjusting the nature of the drug–polymer linker has enabled the easy synthesis of stimuli-responsive polymer prodrugs for efficient spatiotemporal drug release. In this context, this review article will give an overview of the different stimuli-sensitive polymer prodrug structures designed by RDRP techniques, with a strong focus on the synthesis strategies, the macromolecular architectures and in particular the drug–polymer linker, which governs the drug release kinetics and eventually the therapeutic effect. Their biological evaluations will also be discussed.

## Abstract 2

An overview of stimuli-sensitive polymer prodrugs designed by RDRP techniques is presented, focusing on the synthesis strategies, the macromolecular architectures and especially the drug-polymer linkers, which govern drug release kinetics and therapeutic effect.

## Graphical abstract

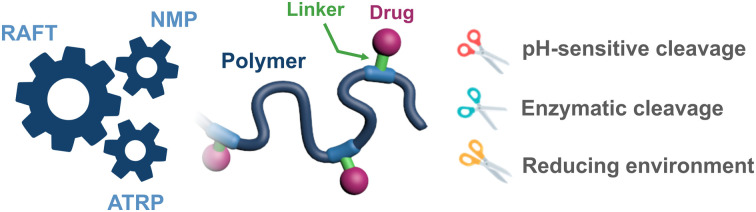

## 1. Introduction

Nanomedicine is now a well-established field of research that is generating a lot of enthusiasm because of its great potential to improve current treatments and enable better diagnosis of many diseases.^1–6^ Most of the current treatments for severe diseases (*e.g.*, cancer) are based on small molecule therapeutics. However, they still face significant limitations and issues such as the occurrence of severe secondary effects due to off-target toxicity, potential early degradation and difficulties in administering poorly soluble drugs. To address these therapeutic challenges, drug-loaded nanocarriers are being extensively studied for their many advantages, such as their ability to prevent early drug release and/or degradation, to allow delivery of poorly soluble drugs, to induce more precise targeted delivery to diseased tissues and cells, and to enable combination therapy.^2,7–12^

Since the lipid vesicles reported in the 1960s,^13^ various families of nanocarriers have been developed, such as liposomes, micelles, nanoparticles or polymersomes, covering a wide range of materials (*e.g.*, organic, inorganic, biological).^14^ Among them, the use of polymers is very popular in the construction of nanocarriers due to their great diversity in nature and properties.^15–18^ Aliphatic polyesters,^19–22^ synthetic polypeptides^23–26^ and natural carbohydrates^27–29^ have long been considered as reference polymers in this field. However, vinyl polymers have received increasing attention as building blocks for nanocarriers, especially since the advent of reversible deactivation radical polymerization (RDRP) techniques, such as nitroxide-mediated polymerization (NMP), atom-transfer radical polymerization (ATRP) and reversible addition–fragmentation chain transfer (RAFT) polymerization,^30–32^ which allow for synthesis of tailor-made polymer architectures.^33^ Vinyl polymers offer numerous advantages such as: (i) their great versatility (*e.g.*, size, nature, composition, properties); (ii) the possibility to obtain nanoparticles with various morphologies (*e.g.*, spherical, vesicular, rod-like, core–shell)^34^ (iii) as well as their ease of synthesis and functionalization, which allows for easy implementation of stimuli-responsiveness^9,35^ for greater therapeutic efficacy and for the grafting of biologically active and/or imaging agents for “theranostic” purposes (*i.e.*, to combine therapeutic and imaging modalities).^15,17,36–38^ In addition, long criticized for their non-degradability which can lead to deleterious side effects when used *in vivo*, vinyl polymers can now be efficiently made (bio)degradable thanks to advances in radical ring-opening polymerization (rROP).^39–42^

Polymer prodrug nanocarriers,^43^ which rely on coupling drugs to the polymer *via* cleavable linkers, have been widely studied as drug delivery systems capable of addressing problems associated with traditional drug-loaded polymer nanoparticles based on physical drug encapsulation.^44–47^ Indeed, the covalent linkage between the drug and the polymer transiently inactivates the drug until it is cleaved, preventing the “burst release” effect from occurring, which can be toxic to patients. Such approach also increases the compatibility of the drug with the polymer matrix and can lead to high drug loadings. Polymer prodrugs therefore allow for improved solubility of poorly soluble drugs and increase their blood circulation time for prolonged drug exposure.

**Fig. 1:**
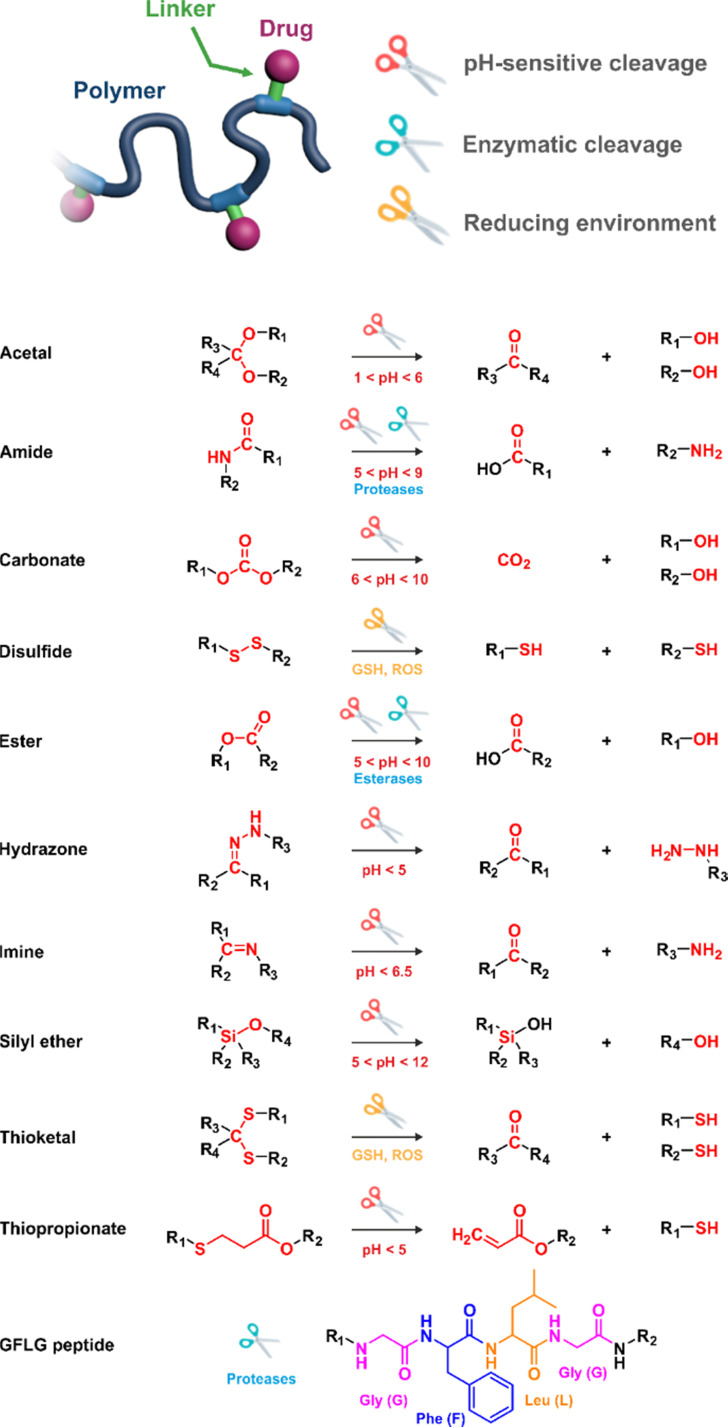
Stimuli-sensitive polymer prodrugs, obtained by reversible deactivation radical polymerization techniques, bearing drug–polymer linkers which can be cleaved by pH variation, the action of specific enzymes or the presence of a reducing environment. GSH = glutathion, ROS = reactive oxygen species.

In these systems, the role of the linker is essential because if properly conceived, it can induce a spatiotemporal release of the drug, which is of paramount importance to minimize off-target toxicity and achieve optimized therapeutic effect.^44^ In this context, drug linkers are usually designed to be sensitive to endogenous stimuli (*e.g.*, pH, enzyme concentration, reducing environment, Fig. 1) or, to a lesser extent, to exogenous stimuli (*e.g.*, light, magnetic field). Such stimuli-responsiveness can be a strong asset for the treatment of pathologies with marked biological specificities like cancer,^48^ which presents, for instance, differences in pH,^49–54^ redox status^55–57^ and/or in concentration of certain enzymes^58–60^ between cancerous and healthy cells.

In recent years, significant progress in the field of polymer prodrug nanocarriers has been facilitated by the use of RDRP techniques, in particular *via* the engineering of advanced systems with precise implementation of linkers sensitive to different endogenous stimuli. More sophisticated polymer prodrug nanocarriers have also been made sensitive to both endogenous and exogenous stimuli, with the exogenous stimulus facilitating cleavage of the linker by the endogenous stimulus, or cleaving it directly.

The objective of this review is to present the recent advances in the field of polymer prodrug nanocarriers obtained by RDRP techniques. The review is focused and articulated on the different synthetic routes to achieve polymer prodrugs and on the nature of the linkers that have been implemented between the drug and the polymer. More specifically, the different stimuli that mediate and/or facilitate their cleavage to optimize drug delivery and therapeutic efficacy will be discussed.

## 2. Reversible deactivation radical polymerization (RDRP)

### 2.1. Main features of RDRP techniques

**Fig. 2:**
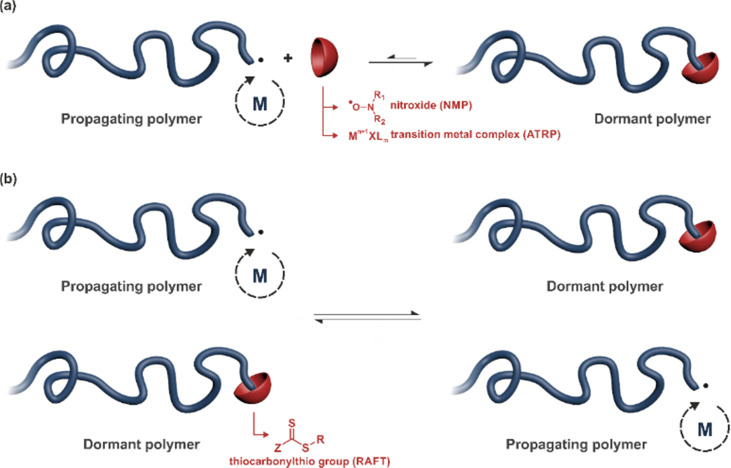
Principle of the main reversible deactivation radical polymerization (RDRP) techniques based on: (a) reversible termination mechanism such as nitroxide-mediated polymerization (NMP) and atom-transfer radical polymerization (ATRP) and (b) reversible transfer mechanism such as reversible addition–fragmentation chain transfer (RAFT) polymerization.

RDRP techniques have become powerful polymerization methods for preparing well-defined polymers with predictable molar masses, low dispersity and sophisticated architectures, with the ability to be functionalized with relative ease.^61–63^ One can distinguish two different polymerization mechanisms to achieve RDRP: (i) reversible termination mechanism (Fig. 2a) and (ii) reversible transfer mechanism (Fig. 2b). Representative RDRP techniques that are based on a reversible termination mechanism are NMP^30,64^ and ATRP,^32,65^ while the RAFT polymerization^66^ is governed by a reversible transfer mechanism. To achieve a good control of polymerizations based on a reversible termination mechanism, the activation–deactivation equilibrium is strongly shifted towards the dormant species to ensure a low concentration of growing macroradicals and minimize termination reactions. For polymerization based on a reversible transfer mechanism, the main equilibrium must allow a rapid exchange of transfer agents between dormant and propagating chains, ensuring homogeneous growth amongst them.

#### 2.1.1. Nitroxide-mediated polymerization (NMP)

The NMP process is based on a reversible termination reaction between a propagating macroradical and a nitroxide to form a macroalkoxyamine^30,64^ (Fig. 2a). The resulting equilibrium between the active macroradical and the dormant macroalkoxyamine is a thermal process as the macroalkoxyamine is homolytically cleaved at elevated temperature to give back the free nitroxide (a typical nitroxide is the *N-tert*-butyl-*N*-[1-diethylphosphono-(2,2-dimethylpropyl)nitroxide], often termed SG1) and the propagating macroradical.

NMP generally operates at temperatures ranging from 70 to 120 °C and is particularly well-suited for the polymerization of a wide range of monomers including styrenics, acrylates, acrylamides and isoprene. Methacrylates can also be successfully controlled by NMP, provided a small fraction of a good comonomer (*e.g.*, styrene, acrylonitrile) is added during the polymerization or a dedicated nitroxide is used. NMP is compatible with many different polymerization processes, such as bulk polymerization, solution polymerization, and polymerization in aqueous dispersed media.

#### 2.1.2. Atom-transfer radical polymerization (ATRP)

ATRP relies on a reversible termination reaction akin to NMP. The living process is based on the reversible activation of halide species by a transition-metal complex (*e.g.*, ruthenium, copper, iron, or nickel) typically coordinated with nitrogen-donor ligands^32,65^ (Fig. 2a). This activation occurs through a redox process entailing *a* ± 1 change in the formal oxidation state of the metal.

ATRP can operate from room temperature and control the polymerization of a broad spectrum of monomers, including styrenics, (meth)acrylates, acrylonitrile and (meth)acrylamides. Interestingly, most (functionalized) initiators and ligands are commercially available. In addition to conventional polymerization processes like bulk and homogeneous organic solutions, ATRP can be performed in aqueous solution, ionic liquids, miniemulsion, microemulsion and emulsion.

#### 2.1.3. Reversible addition–fragmentation chain transfer (RAFT) polymerization

The RAFT technique relies on a reversible transfer reaction occurring between a growing (macro)radical and a (macro)RAFT agent^66,67^ (Fig. 2b). This exchange reaction is established subsequent to the addition of the growing radical Pi˙ onto the dormant species Pj, resulting in an intermediate radical followed by its fragmentation. This process yields the growing radical Pj˙ and the dormant species Pi.

Temperatures reported for RAFT polymerization start from ambient temperature. The RAFT agent can be a thiocarbonylthio group such as dithioester (Z = alkyl), trithiocarbonate (Z = *S*–alkyl), xanthate (*O*–alkyl), or dithiocarbamate (Z = *N*(alkyl)_2_). RAFT has demonstrated a broad applicability as it can control the polymerization of a broad range of functional monomers (*e.g.*, styrenics, (meth)acrylates, acrylic acid, vinyl acetate, isoprene, *etc.*). RAFT polymerization can be carried out in homogeneous media (bulk and solution) as well as in ionic liquids and aqueous dispersed systems such as miniemulsion and emulsion.

### 2.2. Particularities and use of RDRP techniques in a biomedical context

#### 2.2.1. RDRP *vs.* free-radical polymerization for the design of polymer prodrugs

Conversely to free-radical polymerization (FRP), RDRP techniques allow well-defined, functional macromolecular architectures to be synthesized. These specific features are essential when designing polymer-based nanoscale drug delivery systems. Indeed, polymer chains will be homogeneous to each other in terms of length and composition, allowing for less sample-to-sample variability and improved reproducibility in biological assessments.^68,69^ Furthermore, the ability to fine-tune polymer chain length and composition is an important lever for targeting different physico-chemical properties and drug loadings, thus offering greater flexibility in polymer prodrug synthesis.

The use of RDRP techniques also makes it be possible to precisely position drug molecules on the polymer chain, enabling better control of their release. For instance, most effort has focused on coupling drugs to amphiphilic copolymers obtained by controlled polymerization methods.^47^ Various placements of the drug on the copolymer chain have been explored to alter its spatial localization within the resulting nanocarriers, which could significantly affect the rate at which the drug is released. For instance, positioning the drug at the core–shell interface of the nanocarrier may enhance its solvation, potentially accelerating the hydrolysis rate of the drug–polymer linker, which could be advantageous for achieving rapid tumor inhibition. This level of control is not possible with FRP, which makes RDRP methods unique and much more advantageous for the fine tuning of polymer prodrug properties.

Selecting the most appropriate RDRP technique for the synthesis of polymer prodrugs depends on the desired properties of the final conjugate for its intended application. While NMP, ATRP and RAFT polymerization are all powerful synthetic tools^61,63^ for synthesizing functional materials for biomedical applications,^30,70,71^ each offers distinct advantages and limitations. NMP has long been praised for its simplicity and safety (see Section 2.2.2), but it may suffer from a less broad spectrum of monomers that can be controlled and slightly lower efficiency for the synthesis of block copolymers and more sophisticated architectures. ATRP and RAFT polymerizations are undoubtedly more effective in the synthesis of complex macromolecular architectures and in their versatility with regard to the polymerization of monomers of different natures. However, rather extensive purification of ATRP- and RAFT-derived polymers is often required, although recent developments have attempted to mitigate this point (see Section 2.2.2).

#### 2.2.2. Safety and cytotoxicity of RDRP components

When developing new materials for biomedical applications, it is important to guarantee their safety (*e.g.*, biocompatibility), as they are intended for human administration. Controlling agents (*i.e.*, nitroxides for NMP, transition metal catalysts for ATRP and chain transfer agents for RAFT) are the main source of potential toxicity associated with the use of RDRP techniques.

While *in situ* NMP is often associated with high toxicity of C-nitroso compounds as precursors of nitroxides,^72^ polymers synthesized by traditional NMP appeared safe and innocuous for use in biomedical applications. For instance, exposure of three different healthy cell lines (HUVEC, NIH/3T3 and J774.A1) to water soluble copolymers based on poly(oligo(ethylene glycol)methyl ether methacrylate (POEGMA) obtained using the BlocBuilder alkoxyamine resulted in high cell viabilities (∼80%) and no changes in morphological appearance and cell density up to 1 or 10 mg mL^−1^, depending on the nature of the comonomer.^73^ To be noted that such concentrations are not representative of typical therapeutic doses administered in clinical trials or biomedical assays. These high doses were chosen to amplify any potential cytotoxic effects stemming from the monomer(s), the copolymers, as well as from the presence of the nitroxide (SG1) end group. Moreover, to rule out potential cytotoxicity from the SG1 nitroxide itself, which could be released by homolytic cleavage from the polymer in the long run, further cell viability assays at concentration mimicking quantitative release of SG1 from polymers at 10 mg mL^−1^ gave >90% cell viability.^73^ These results clearly evidenced the safety profile of these polymers and of the SG1 nitroxide.

The cytotoxicity of ATRP polymers is mostly related to the use of transition metal complexes (often based on copper) as catalysts and, in particular, to the efficiency of residual catalyst removal after polymerization. Purification methods such as precipitation, dialysis or the use of ion exchange resins, which also enable copper to be recovered and recycled, are usually applied.^74^ Recent development in ATRP have also made it possible to easily produce safe polymers for use in biomedical applications.^75^ For instance, ATRP systems using ppm amount of copper catalysts,^75,76^ such as supplemental activator and reducing agent (SARA) and initiators for continuous activator regeneration (ICAR) ATRP, have been developed, guaranteeing minimal (and almost negligible) amounts of trace copper after purification. In SARA ATRP, some key examples are the use of reducing agents such as tin(ii) 2-ethylhexanoate, which is FDA-approved, or glucose and ascorbic acid, which are biocompatible. The use of harmless metals is also an interesting option for ensuring the production of non-cytotoxic polymers.^75^ In this context, considerable interest has been focused on iron complexes, owing to their low toxicity, inexpensive cost, commercial availability and innocuousness compared to copper-based catalysts. More recently, metal-free ATRP processes, including organo-catalyzed ATRP,^77^ have been extensively studied to overcome the challenge of metal contamination in traditional ATRP systems.^75^ For example, 10-phenylphenothiazine^78^ or diaryl dihydrophenazines^79^ have been successfully used as reducing photoredox catalysts to produce well-defined polymers by photo-mediated ATRP. Enzyme-mediated ATRP,^75^ has also garnered significant attention because of its high efficiency and selectivity, mild reaction conditions, and excellent compatibility with biological systems. Typically, they exhibit a high catalytic turnover rate and are readily separable from the reaction products. Some typical examples used metalloenzymes, such as HRP,^80^ hemoglobin,^81^ catalase, or laccase.^82^

In the case of polymers obtained by RAFT, cytotoxicity depends on the nature of the RAFT agent.^83^ For instance, ω-dithiobenzoate-ended poly(*N*-(2-hydroxypropyl)methacrylamide) (PHPMA) showed important cytotoxicity on CHO-K1, NIH/3T3 and Raw264.7 cell lines at high concentration (1 000 μM), whereas the ω-trithiocarbonate-ended counterparts were not cytotoxic under the same conditions.^84^ However, no cytotoxicity on CHO-K1 and NIH/3T3 cell lines was observed with POEGMA and poly(oligo(ethylene glycol)methyl ether acrylate) (POEGA), irrespective of the RAFT end group, whereas the Raw264.7 cells were more sensitive to ω-dithiobenzoate end-groups with a cell viability dropping to 73% after 24 h.^84^ Interestingly, at a concentration of 200 μM, no cytotoxic effect was obtained. Further studies confirmed the safety of trithiocarbonate-based polymers, such as POEGMA star polymers obtained from 3-benzylsulfanylthiocarbonylsulfanyl-propionic acid as a RAFT agent, which were noncytotoxic (below 10 mg mL^−1^) on MRC-5 fibroblasts and SH-SY5Y cancer cells.^85^ Studies have also been carried out to assess the *in vitro* cytotoxicity of free RAFT agents. For instance, incubation of L929 fibroblasts^86^ with 3-benzylsulfanylthiocarbonylsulfanyl-propionic acid resulted in only 9.7% of cell growth inhibition, whereas benzyl dithiobenzoate inhibited almost 72% of cell growth.^87^ Xanthates, such as methyl [(ethoxycarbonothioyl)sulfanyl)]acetate, have also been associated with significant cytotoxicity (85% L929 cell death after 24 h). Importance of the nature of the RAFT agent on cytotoxicity was also confirmed by comparing dithioester- and trithiocarbonate-POEGAs, demonstrating the cytotoxicity of the former and the safely of the latter during cell viability assays on NIH 3T3 cells up to 10 mg mL^−1^.^88^ Importantly, if toxicity of the RAFT moiety is a matter of concern, several effective removal methods have been reported for generating polymers without RAFT end group.^89^

Impurities may also play a role in the toxicity associated with RDRP-derived polymers. RDRP techniques often use organic solvents that may be associated with residual toxic traces, a serious issue for biomedical applications. Ongoing research is therefore focused on the discovery of greener solvents such as water, ionic liquids or deep eutectic solvents for ATRP,^90^ poly(ethylene glycol) for RAFT,^91^ or cyclopentyl methyl ether^92^ and supercritical carbon dioxide (scCO_2_) for NMP.^93^

#### 2.2.3. Implementation of RDRP techniques for the design of polymer prodrugs

**Fig. 3:**
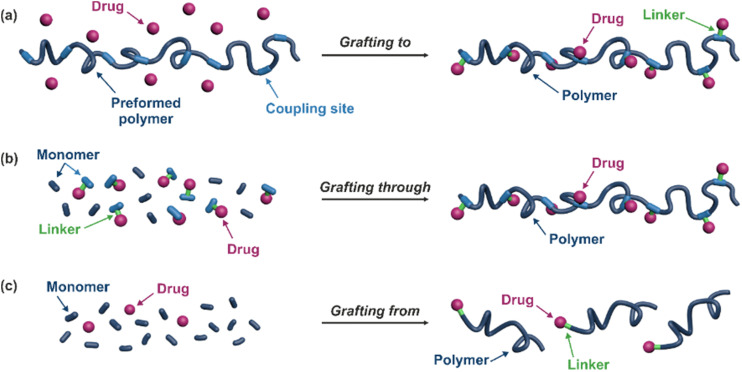
Synthesis of polymer prodrugs by: (a) the grafting to approach (based on the coupling of drug to a preformed polymer); (b) the grafting through approach (based on the (co)polymerization of drug-monomer molecules) and (c) the grafting from approach, also called drug-initiated method (based on the growth of a polymer chain from a drug).

Polymer prodrugs synthesized by RDRP techniques can be obtained *via* three main synthetic routes:^47,94^ (i) the “grafting to” strategy, which consists in coupling the drug to a preformed polymer (Fig. 3a); (ii) the “grafting through” strategy, which consists in grafting a drug onto a monomer prior to polymerization (Fig. 3b) and (iii) the “grafting from”, also called “drug-initiated“ strategy, which consists in the polymerization of monomers from a drug functionalized by a RDRP controlling agent (Fig. 3c). Each synthetic route has its own characteristics, advantages and disadvantages.

The “grafting to” method is certainly the most popular way to produce polymer prodrugs and is praised for its versatility.^47,95,96^ Free drugs can be conjugated to various preformed polymers, allowing for flexibility in choosing both components. However, this approach offers limited control over the final product's structure, composition and drug distribution along the polymer backbone, potentially impacting the drug release. Additionally, depending on the chosen chemistry, the drugs may interfere with each other during coupling due to excessive steric hindrance, or stack up due to hydrophobic interactions, thus reducing the conjugation efficacy. Purification of the final conjugate may also be challenging as unreacted drugs and polymer chains must be separated from the desired conjugate.

The “grafting through“ method provides greater control over the polymer prodrug's structure.^47^ By coupling drug molecules to functional monomers, this technique allows for precise design of the polymer backbone, especially by using RDRP techniques. Additionally, drugs are usually uniformly distributed throughout the polymer chain during polymerization, leading to a more homogenous distribution within the final polymer prodrug. However, this approach requires suitable chemical functionalities on both the drug and the chosen monomers. Furthermore, incorporating bulky drug molecules within the polymer chain might limit the achievable drug loading capacity compared to the other methods.

Finally, the “grafting from” method, only achievable using RDRP techniques (and controlled polymerization in general) offers the most versatility and simplicity.^97^ This approach allows for the synthesis of drug–polymer conjugates bearing one drug molecule attached at the extremity of a well-defined polymer chain. This is achieved by derivatization of the drug with RDRP controlling agents followed by polymerization of the desired monomer. It offers valuable advantages compared to other methods. For instance, a nearly quantitative conjugation efficiency is obtained, as all the drugs should be retained at the chain ends. The purification of the conjugates is facilitated, since only the unreacted monomer (often a volatile) has to be removed. Finally, high and tunable drug loadings can be easily obtained by varying the polymer chain length. Owing to the living nature of the polymer prodrugs obtained by drug-initiated RDRP, more sophisticated systems can be constructed by applying post-functionalization methods and taking advantage of the presence of the controlling agent at the other chain end to introduce other molecules of interest.^98–100^ This was illustrated by the synthesis of heterotelechelic polymer prodrugs for combination therapy or theranostic applications.

## 3. Linking drugs to preformed polymers (grafting to)

The most used method to synthesize polymer prodrugs relies on direct conjugation of the drug to a preformed polymer *via* post-functionalization, also termed “grafting to” method (Fig. 3a). RDRP techniques have been extensively used to elaborate well-defined amphiphilic copolymers for subsequent self-assembly into nano-objects.^101–105^ Interestingly, the compatibility of RDRP with a wide range of functional groups and its ability to achieve complex macromolecular architectures allow predetermined positioning of functionalization sites on the polymer structure for subsequent coupling with drugs. This has direct consequences on the localization of the drugs (*e.g.*, on the side chain or chain end of the copolymer, on the shell or in the core of the nano-object, *etc.*) and thus on their release kinetics. The “grafting to” strategy also allows drugs to be bound to polymers before or after their self-assembly, thus providing greater flexibility in achieving the desired structure.

The post-functionalization step is usually based on a library of well-established organic chemistry reactions such as Schiff base reaction, esterification or amidation, the choice of which is governed by the nature of the available functional groups on the polymer and on the selected drug. Once the coupling is achieved, it results in the introduction of a linker (*e.g.*, hydrazone, ester, amide, disulfide, *etc.*) between the polymer scaffold and the drug, which can be selectively cleaved under the action of endogenous stimuli such as pH, redox conditions, or the presence of specific enzymes in the biological environment (Table 1). The choice of the linker may also be dictated by the nature of the diseased area, as in the case of the tumor microenvironment, which presents intrinsic singularities and/or dysregulations that could be precisely targeted. The additional application of an external stimulus, such as the temperature, may achieve enhanced or more controlled drug release in targeted areas (see Table 2, Section 2.2).

**Table 1:** Stimuli-sensitive drug linkers in prodrugs obtained by RDRP techniques *via* the “grafting to” strategy

Linker	Drug	Polymerization method	Polymer prodrug	Cleavage conditions	Release	Ref.
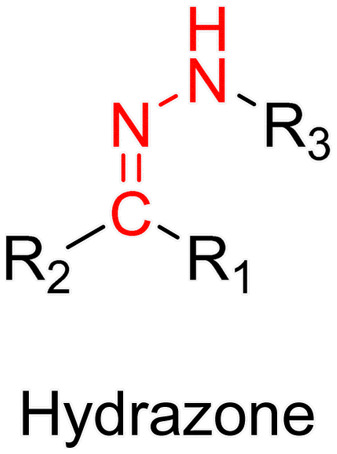	Dox	RAFT	P(MPC-*co*-Ada)	pH 5	43% after 48 h	115
	Dox	ATRP	PMCP-*b*-PMEMA	pH 5	60% after 65 h	110
	Dox	ATRP	P(MPC-*co*-TBOEMA)	pH 5	80% after 48 h	117
	Dox	RAFT	POEGMA-*b*-P(MAH-*co*-Rh6GEAm)	pH 5	73% after 72 h	118
	Dox	RAFT/ROP	PMaIpGP-*b*-POEGMA-*b*-P(Llys-*co*-Asp)	pH 5.4	65% after 72 h	119
	Dox	RAFT	HMSNs + P(OEGMA-*co*-MABH)	pH 5	18% after 58 h	121
				pH 6	69% after 58 h	
	Dox	ATRP/ROP	PBYP-*SS*-P(DMAEMA-*co*-FBEMA)	pH 5 + GSH 10 mM	70% after 70 h	122
	Pt(ii)	RAFT	POEGMA-*b*-PHEMA	—	—	111
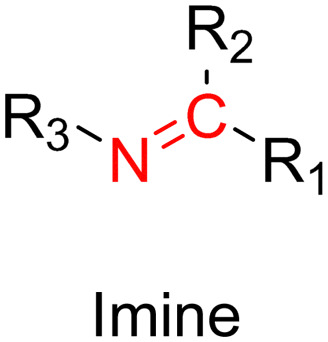	Dox	ATRP	PLlys-*b*-PMPC	pH 5.5	80% after 48 h	123
				pH 6.8	<50% after 48 h	
	Dox	ATRP	PEG-P(GMA-CBA)	pH 5	80% after 12 h	124
	Dox	RAFT	P(MPC-*co*-POEGMA-Bz)	pH 5	70% after 140 h	125
	Dox	RAFT	P(OEGMA-*co*-FPMA)-*b*-PDPA	pH 6.5	40% after 30 h	126
				pH 5.5	80% after 4 h	
	Dox	ATRP	β-CD-*star*-P(DEAEMA-*co*-FPMA)-*b*-POEGMA	pH 5	45% after 48 h	127
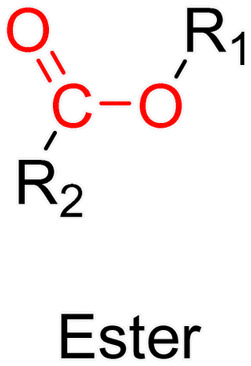	Oxoplatin	RAFT	POEGMA-*b*-PMAA	pH 5	∼50% after 30 h	133
	CDDP	RAFT	POEGMA-*b*-PMANHS-*b*-PMAETC + cross-linker: ketal diamine	pH 5.5	∼75% after 72 h	135
	Cisplatin	ATRP	POEGA-*b*-PGAP-*b*-POEGA	pH 5.6	60% after 20 h	136
	Auranofin	RAFT	PHEA-*b*-P(4-AuPEt_3_)	—	—	137
	SN-38	ROP/ATRP	P(ACL-*co*-CL)-*b*-PMPC	Esterases	70% after 70 h	162
	RAPTA-C	ROP/RAFT	PLA-*b*-P(HEA-*co*-CEMA)	Hydrolases	Complete disassembly of the micelles	163
	Cpt	RAFT	P(OEGMA-*co*-BSMA-*co*-G3-C12)	Esterases	77% after 24 h	166
	Buf	ATRP/RAFT	P(OEGMA-*co*-BSTMA)-*g*-P(DEAEMA-*co*-BMA)	Esterases	83% after 24 h	169
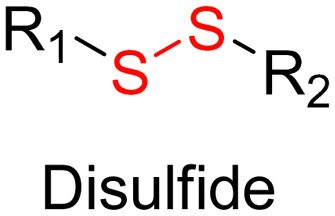	Vorinostat	RAFT	POEGMA-*b*-PS	GSH 10 mM	Vorinostat: 70% after 12 h	146
	Tamoxifen				Tamoxifen: 40% after 48 h	
	Gem	RAFT	PMPC-*b*-P(DEAEMA-*co*-MMA-*co*-TPMA)	GSH 10 mM + pH 5	95% after 48 h	149
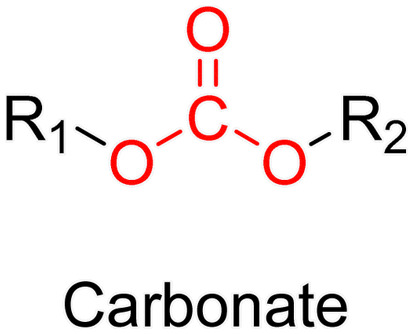	Cpt	RAFT	h-P(GMA-*co*-OEGMA)-*b*-POEGMA	pH 5 + GSH 10 mM	45% after 96 h	147
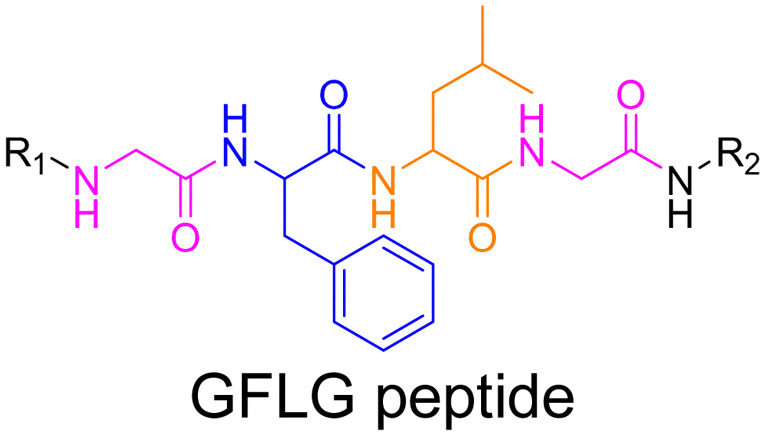	Dox	RAFT	PDHPMA	pH 5 + cathepsin B	82% after 10 h	175
	Dox	RAFT	POEGMA	pH 5.4 + cathepsin B	78% after 12 h	176

**Table 2:** Dual-sensitive polymer prodrugs based on the combination of endogenous stimuli-sensitive linkers and external stimuli, obtained *via* the “grafting to” strategy

External stimuli	Drug	Drug linker	Polymerization method	Nanocarrier	Cleavage conditions	Release	Role of external stimuli	Ref.
Temperature	Dox	Hydrazone	RAFT/ATRP	Alkyne-P(HPMA-*st*-EGMA)-*SS*-PNIPAAm	pH 5.5 + GSH 10 mM	80% after 48 h	Micelles formation at 37 °C	179

### 3.1. The use of endogenous stimuli

#### 3.1.1. pH-Sensitivity

Polymer prodrug nanocarriers synthesized by the “grafting to” approach have been equipped with different pH-responsive linkers to achieve tumor-targeted drug delivery owing to their ability to detect changes in pH within the body and in particular in the tumor microenvironment.^49–54^

##### 3.1.1.1. Hydrazone linker

The hydrazone moiety represents one of the most used linkers for developing pH-responsive polymer prodrugs nanocarriers due to its ease of formation and incorporation into polymers. The synthesis of the hydrazone bond relies on the condensation of hydrazine or hydrazide-containing compounds with aldehyde or ketone derivatives (Fig. 1). This chemical linkage as a Schiff base bond is sensitive to slightly acidic pH (∼5–6)^106^ and remains extremely stable from physiological pH and above (>7.4). The nature of the carbonyl group (ketone or aldehyde) and its substituents can strongly affect the lability and stability of the hydrazone bond formed.^107^ Recent progress have been made to accelerate the formation of hydrazone bonds notably by improving the rate and the versatility of the condensation. This aimed to propose more efficient bioconjugation of molecules (that should possess either a carbonyl moiety or an alpha-nucleophile group) such as reducing sugars, peptides or proteins.^108^ In the context of the “grafting to” approach, the main strategy to introduce hydrazone linkers into prodrugs is based on the conjugation between a drug, containing a ketone or an aldehyde group, and a polymer functionalized with hydrazine moieties *via* hydrazinolysis of ester groups or acylhydrazine formation.^109,110^ If no functional group is available on the drug molecule, a ketone or an aldehyde group can be grafted onto the polymer *via* hydrazone linkage prior to drug conjugation, such as a diamino-ketone ligand in the case of platinum (Pt).^111^ Such hydrazone linker allows an acid-sensitive drug release in intracellular biological compartments such as endosomes (pH ∼ 5–6)^112^ and lysosomes (pH ∼ 4–5)^112,113^ but also more specifically in the tumor microenvironment, which is characterized by a slightly lower extracellular pH comprised between 6.5 and 7.2^114^ compared to that of healthy cells (pH ∼ 7.4). The relatively facile formation and incorporation of hydrazone bonds into polymer prodrug systems, combined to their physiological stability and acid-sensitivity represent major advantages for their use as drug linkers, especially in anticancer drug delivery systems.

**Fig. 4:**
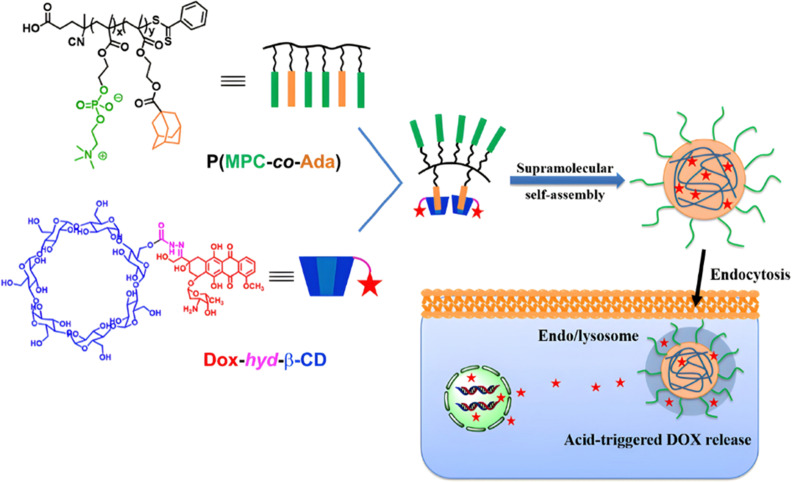
Amphiphilic P(MPC-*co*-Ada) copolymer conjugated to Dox-hyd-β-CD *via* host–guest interactions and its pH-driven drug release in endosomes/lysosomes. Adapted from ref. 115.

Due to its tertiary alpha-hydroxy ketone in its structure, the anticancer agent doxorubicin (Dox) is perhaps the most representative drug used for the development of pH-sensitive polymers *via* hydrazone bonding by RDRP. For instance, pH-sensitive, Dox-loaded polymer prodrug nanoparticles have been successfully obtained from amphiphilic random copolymers containing zwitterionic monomer units and pendant adamantane (Ada) moieties, the latter being able to form inclusion complexes with Dox-hydrazone-β-cyclodextrin (Dox-*hyd*-β-CD) through host–guest interactions (Fig. 4).^115^ P(MPC-*co*-Ada) copolymers were synthesized by RAFT copolymerization of 2-methacryloyloxyethyl phosphorylcholine (MPC) with 2-(methacryloyloxy)ethyl adamantane-1-carboxylate (MEAC), while Dox-*hyd*-β-CD was obtained by converting some hydroxyl groups of β-CD into activated esters, followed by reaction with hydrazine monohydrate and Dox coupling under acidic conditions. A similar approach was also reported from Dox-*hyd*-β-CD and ferrocene-conjugated poly(ethylene glycol) (Fc-PEG). The resulting polymer prodrugs were able to self-assemble into nanoparticles of 83 nm diameter and to release Dox in a controlled, acid-sensitive manner at endosomal pH. At pH 5, Dox release increased 1.3-fold compared with release at pH 7.4 (∼32% after 48 h). These nanoparticles were almost completely internalized in HepG2 cells after 5 h, demonstrating rapid internalization. Eventually, *in vitro* 3-[4,5-dimethylthiazol-2-yl]-2,5 diphenyl tetrazolium bromide) (MTT) assays showed dose-dependent cytotoxicity on HUVECs and HepG2 cells.^116^

**Fig. 5:**
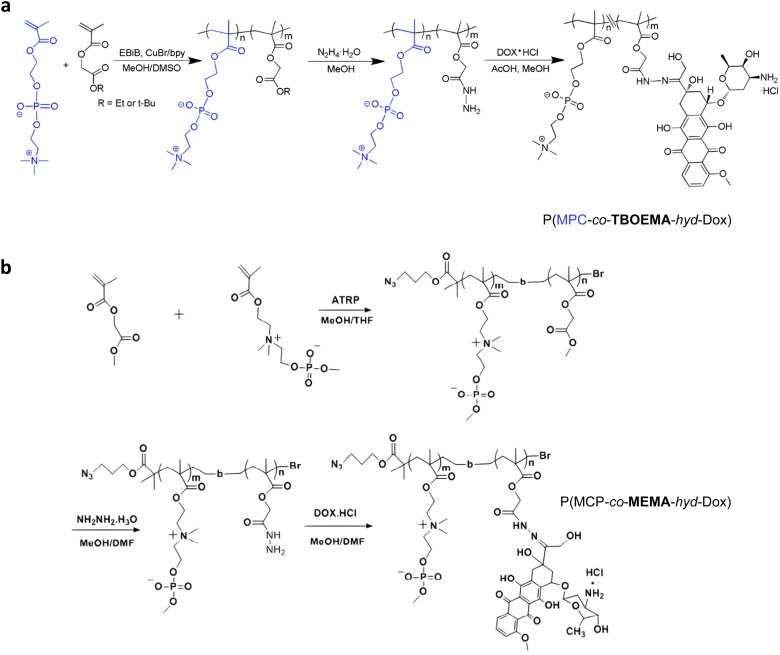
Synthetic strategies for: (a) P(MPC-*co*-TBOEMA-*hyd*-Dox) and (b) P(MCP-*co*-MEMA-*hyd*-Dox) by ATRP. Adapted from ref. 110 and 117.

Direct grafting of Dox to a polymer scaffold to produce pH-sensitive polymer prodrug nanoparticles can be achieved from poly(2-methacryloyloxyethyl phosphorylcholine)-*co*-2-*tert*-butoxy-2-oxoethyl methacrylate) (P(MPC-*co*-TBOEMA)) random copolymers obtained by ATRP (Fig. 5a).^117^ After ester-to-acyl hydrazine conversion, using hydrazine hydrate, Dox conjugation was carried out to achieve the corresponding pH-sensitive PMPC-*b*-TBOEMA-*hyd*-Dox polymer prodrug with 15–45 wt% drug loading and 7–15 nm in diameter depending on the Dox content. The Dox release was pH-dependent, with half-life time ranging from 2 to 40 h at pH 5, and cell internalization experiments showed that the higher the drug loading, the higher the intracellular internalization and the cytotoxicity. In addition, the polymer prodrug with 30 wt% drug loading exhibited maximum tolerated doses in the range of 30–50 mg kg^−1^ Dox equiv. in mice. A very similar post-polymerization approach was carried out *via* the synthesis of poly(2-(methacryloyloxy)ethyl choline phosphate)-*block*-poly(2-methoxy-2-oxoethyl methacrylate) (PMCP-*b*-PMEMA) by ATRP (Fig. 5b).^110^ Subsequent hydrazinolysis of MEMA moieties in the presence of hydrazine hydrate, enabled conjugation of Dox under acidic conditions, resulting in PMCP-*b*-PMEMA-*hyd*-Dox polymer prodrug aggregates of 180 nm in diameter and 10 wt% drug loading. Dox release was shown to be pH-dependent as only ∼6% of Dox was released at pH 7.4 whereas ∼60% was released at pH 5 after 65 h. This polymer prodrug was also efficiently internalized by MCF-7 breast cancer cells within 1 h and appeared to be cytotoxic on three different cancer cell lines (MCF-7, A549, HepG2) while the drug-free copolymer showed good cytocompatibility. Surprisingly, despite a small structural difference in monomer structure (MCP *vs.* MPC and MEMA *vs.* TBOEMA) between these two studies, significant differences in drug loadings (15–45 wt% *vs.* 10 wt%, respectively) were obtained.

Instead of post-functionalizing with free Dox, a one-pot ATRP/click chemistry coupling process was developed to yield pH-sensitive PMPC-based polymer prodrugs. After derivatization of Dox with a hydrazone-azide linker, the resulting Dox-*hyd*-azide was reacted with trimethylsilyl-protected propargyl methacrylate (TMS-PgMA) during its copolymerization with MPC by ATRP. Even if the desired pH-sensitive structures were obtained with good control (*M*_n_ = 6700–12 400 g mol^−1^, *Đ* = 1.23–1.40), they exhibited a rather poor drug loading (3–5 wt%).^117^

Theranostic polymer prodrugs based on the hydrazone linker for the release of Dox were also developed by RDRP *via* covalent linkage of a fluorescent dye onto the polymer backbone. This strategy could enable real-time fluorescence imaging of tumor tissue combined with controlled release tailored to the tumor microenvironment. Two different strategies were investigated: the copolymerization of a fluorescent dye-bearing monomer during the polymer prodrug synthesis or the grafting of the fluorescent dye onto a preformed polymer prodrug. In the first strategy, sequential RAFT polymerization was used to synthesize a poly(oligo(ethylene glycol)methyl ether methacrylate)-*block*-poly(methylacryloylhydrazide-*co*-Rhodamine 6G ethyl acrylamide) (POEGMA-*b*-P(MAH-*co*-Rh6GEAm)) amphiphilic diblock copolymer to which Dox was grafted through an acylhydrazone bond.^118^ The copolymer was able to self-assemble into micelles of 50 nm in diameter, with enhanced Dox release at pH 5 (73%) and pH 6.5 (42%) after 72 h compared to physiological pH (13%). They also demonstrated significant cytotoxicity on HepG2 cells with 23% of cell viability at 0.1 mg mL^−1^. The second strategy relied on the combination of RAFT polymerization, ring-opening polymerization (ROP) and click chemistry to produce a poly(6-*O*-methacryloyl-1,2:3,4-di-*O*-isopropylidene-d-galactopyranose-*block*-poly(oligo(ethylene glycol)methyl ether methacrylate)-*block*-poly(carbobenzoxy-l-lysine-*co*-l-aspartic acid-4-benzyl ester) triblock copolymer (PMaIpGP-*b*-POEGMA-*b*-P(Llys-*co*-Asp)).^119^ Dox was conjugated *via* hydrazone bonding on Asp units after displacement of the benzyloxy groups with hydrazine. The cyanine dye, which is suitable for near-infrared fluorescence (NIR) imaging, was conjugated to the terminal amine group of the polypeptide block. The resulting fluorescent polymer prodrug was able to self-assemble into 90 nm-micelles, which showed enhanced Dox release at pH 5.4 compared to physiological conditions after 72 h (65% and 31%, respectively). The micelles also demonstrated enhanced uptake by HepG2 and NIH3T3 cells due to the presence of galactose units acting as targeting ligand.

**Fig. 6:**
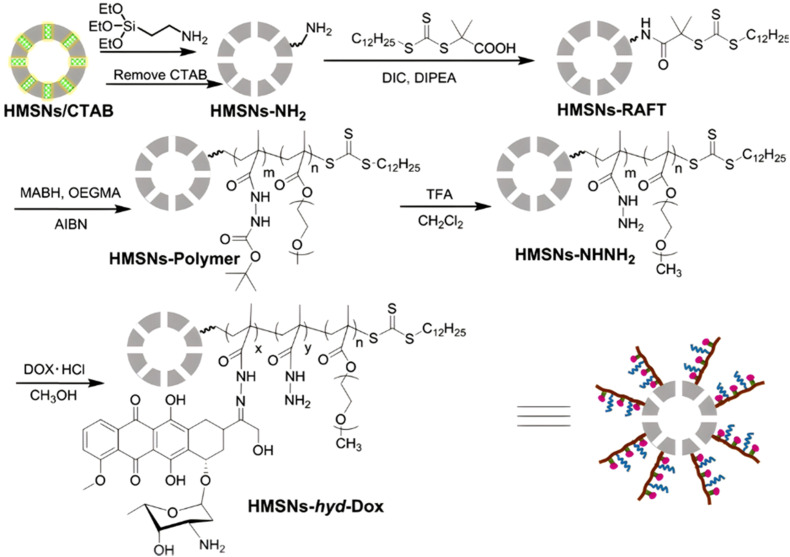
Synthetic route of hollow mesoporous silica nanoparticles (HMSNs) and covalent linkage of Dox *via* hydrazone formation (HMSNs-hyd-Dox). Adapted from ref. 121.

Inorganic silica nanoparticles have also been decorated with polymers from RDRP in which hydrazone linkages have been incorporated to obtain theranostic systems. Mesoporous silica nanoparticles are considered promising candidates for theranostic applications,^120^ particularly as they can both allow the encapsulation of a fluorescent dye in their pores and the grafting of polymers onto their surface. For instance, random copolymerization of OEGMA and methacrylamide *tert*-butyl carbazate (MABH) was achieved from RAFT agents immobilized at the surface of hollow mesoporous silica nanoparticles (HMSNs).^121^ This step was followed by the coupling of Dox to the acylhydrazine groups of MABH (Fig. 6) and the encapsulation of the IR825 photothermal dye into the hydrophobic hollow cavity of silica. The resulting HMSNs-*hyd*-Dox@IR825 polymer prodrug hybrid nanoparticles exhibited an average diameter of 120 nm and showed enhanced Dox release in mild acid conditions (*i.e.*, pH 5 and 6) compared to pH 7.4 (76%, 69% and 18% after 58 h, respectively). *In vitro* studies on HeLa cells confirmed significant cytotoxicity of such particles even if it was lower than that of free Dox and fast internalization as strong Dox fluorescence was observed in the cell nucleus region after 24 h.

**Fig. 7:**
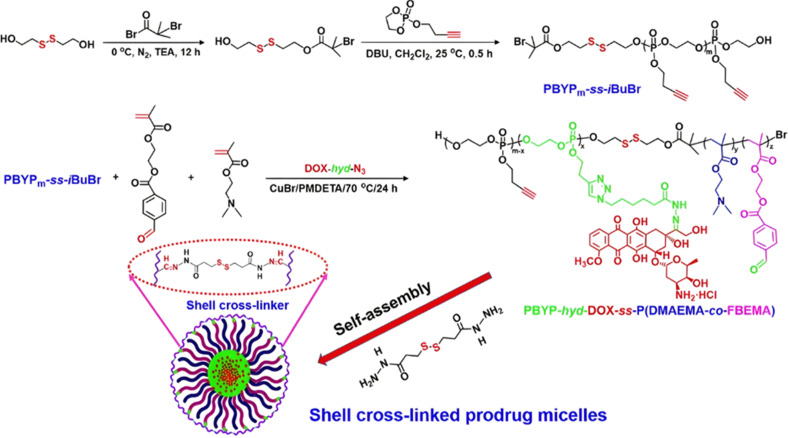
Synthesis route, self-assembly and cross-linking of PBYP-*hyd*-DOX-*SS*-P(DMAEMA-*co*-FBEMA) polymer prodrug. Adapted from ref. 122.

Dual-sensitive diblock copolymer prodrug micelles of Dox based on hydrazone drug linkers and disulfide bonds for colloidal disassembly have also been proposed.^122^ A poly(2-(but-3-yn-1-yloxy)-2-oxo-1,3,2-dioxaphospholane) (PBYP) first block end-functionalized by an ATRP initiator through a disulfide bond was obtained by ROP. Its chain extension by ATRP with *N*,*N*-(2-dimethylamino)ethyl methacrylate (DMAEMA) and 2-(4-formylbenzoyloxy)ethyl methacrylate (FBEMA) gave a PBYP-*SS*-P(DMAEMA-*co*-FBEMA) diblock copolymer which was then reacted with an azide group-containing Dox-hydrazone derivative (Dox-*hyd*-N_3_), by click chemistry. The resulting PBYP-*hyd*-DOX-*SS*-P(DMAEMA-*co*-FBEMA) self-assembled into 144 nm-micelles and further stabilized with a disulfide bond-containing crosslinker (Fig. 7). The combination between the acid-sensitive hydrazone Dox linker and the presence of disulfide bonds in the copolymer structure resulted in optimal drug release at pH 5 in presence of 10 mM GSH (70% after 70 h), likely due to fast micelle disassembly. The crosslinked polymer prodrug micelles exhibited significant cytotoxicity on HeLa and HepG2 cells, even if the free drug was more cytotoxic, which is explained by the time required to cleave the covalent linkage between the drug and the polymer backbone.

**Fig. 8:**
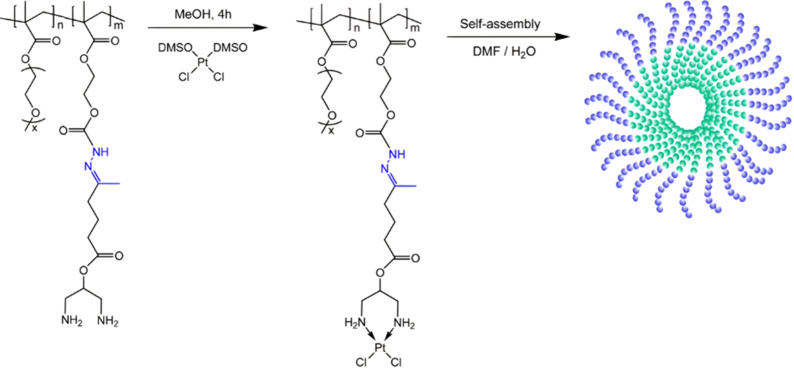
Synthesis of POEGMA-*b*-PHEMA-*hyd*-Pt copolymer and self-assembly into polymer prodrug micelles. Adapted from ref. 111.

Anticancer platinum drugs have also been conjugated *via* hydrazone linkage to amphiphilic diblock copolymers made by RDRP. Hydrazide functionalities were introduced on a poly(oligo(ethylene glycol)methyl ether methacylate-*block*-2-hydroxyethyl methacrylate) (POEGMA-*b*-PHEMA) copolymer obtained by RAFT polymerization *via* a two-step post-modification using 4-nitrophenyl chloroformate and hydrazine monohydrate.^111^ A ketone-functional diamino ligand was then installed onto the copolymer *via* hydrazone linkage, allowing platinum conjugation (Fig. 8). Self-assembly of POEGMA-*b*-PHEMA-*hyd*-Pt polymer prodrugs led to Pt(ii)-containing acid-degradable polymer prodrug micelles of 27 nm in diameter and exhibiting significant cytotoxicity on ovarian cancer cells.

##### 3.1.1.2. Imine linker

The imine group is another Schiff base bond widely used as acid-labile linkage for the design of pH-sensitive polymer prodrugs by RDRP. It is formed by condensation reaction between an aldehyde or a ketone moiety and a primary amine group (Fig. 1). It usually degrades at pH < 5–6 and remains stable at physiological pH, which facilitates its use for drug delivery applications by enabling a precise control of the drug release under physiopathological conditions.

**Fig. 9:**
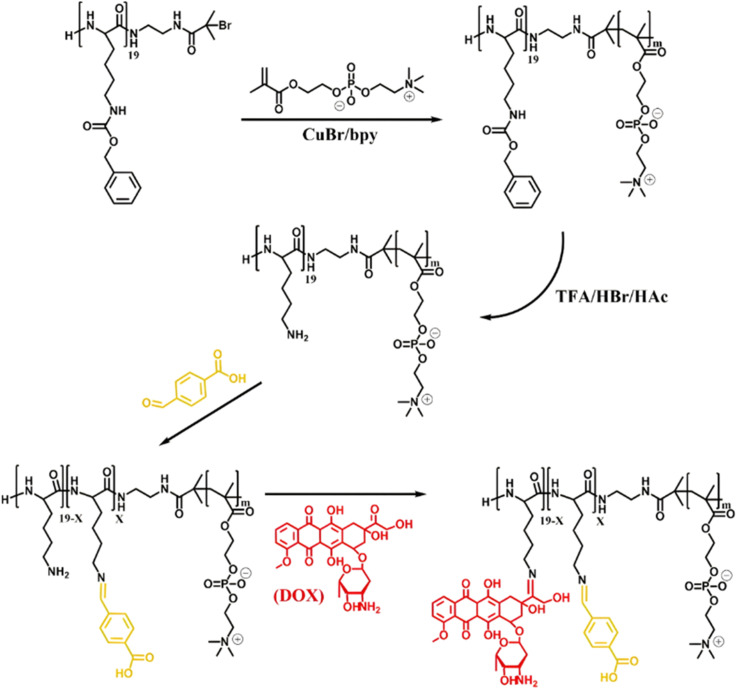
Synthesis of pH-sensitive Dox-based polymer prodrug micelles with charge conversion capability composed of PMPC as hydrophilic shell and PLlys-*imine*-Dox as hydrophobic core. Adapted from ref. 123.

Owing to its primary amine group, Dox has also been conjugated to polymer prodrugs obtained by RDRP *via* the formation of imine linkage. A PLlys-*b*-PMPC copolymer was prepared by ATRP of MPC from a protected PLlys-based ATRP macroinitiator.^123^ The PLlys block was then deprotected to release its primary amine groups which were sequentially conjugated to 4-carboxylbenzaldehyde (CBA) to confer a pH-dependent charge conversion property, and to Dox *via* formation of imine linkages (Fig. 9). Due to the amphiphilic nature of the resulting polymer prodrug, it formed micelles of 90 nm diameter with PMPC as the hydrophilic shell and PLlys-*imine*-Dox as the hydrophobic core. They exhibited accelerated drug release kinetics due to: (i) a surface pH-triggered charge conversion and (ii) a pH-dependent structural disassembly due to imine bond hydrolysis and Dox release. Interestingly, due to the inherent imine linkage stability, the micelles remained stable at physiological pH over 30 days. Cell internalization studies showed greater endocytosis of the micelles at pH 6.8 than at pH 7.4, in addition to a more efficient drug release under acidic environment, leading to a similar cytotoxicity and cell apoptosis level on 4T1 and HeLa cells compared to those of the free drug.

Another strategy to achieve similar amphiphilic polymer prodrugs is to polymerize glycidyl methacrylate (GMA) from a linear PEG-functionalized ATRP macroinitiator, which epoxy rings were post-functionalized by CBA to install pendant aldehyde groups for subsequent Dox conjugation *via* imine bond formation.^124^ This polymer prodrug was able to self-assemble into micelles, whose average diameters ranged from ∼100 nm for a PGMA average chain length of 21 repeat units to 260 nm for 89 repeat units. A pH-triggered Dox release up to 80% within 12 h was observed, which was explained by a conformational modification of the hydrophobic core into a semi-hydrophobic one under acidic conditions leading to an improved diffusion of the protons H^+^. A fast internalization of the micelles in HepG2 cells followed by a pH-driven release of Dox into the cytosol and its accumulation in the nucleus were demonstrated by confocal microscopy.

Pendant Dox molecules can be similarly installed on a copolymer backbone *via* RAFT copolymerization of MPC with oligo(ethylene glycol) methacrylate ester benzaldehyde (OEGMA-Bz) to achieve P(MPC-*co*-POEGMA-Bz), to which Dox was grafted *via* imine bond formation. The resulting P(MPC-*co*-POEGMA-Bz-*imine*-Dox) copolymer prodrug was then functionalized at the chain-end with folic acid (FA) using click chemistry for the targeting of tumor-overexpressed FA receptors. Prodrug nanoparticles of 140 nm diameter with high Dox loadings (up to 28 wt%) exhibited selective release of Dox and colloidal disassembly at pH 5, as shown by dynamic light scattering (DLS) and transmission electron microscopy (TEM). The FA-decorated nanoparticles led to greater internalization in HeLa cells compared to their non-targeted counterparts. However, the cytotoxicity was rather similar with or without targeting ligand, which may be explained by a partial accessibility of FA to folic acid receptors.^125^

**Fig. 10:**
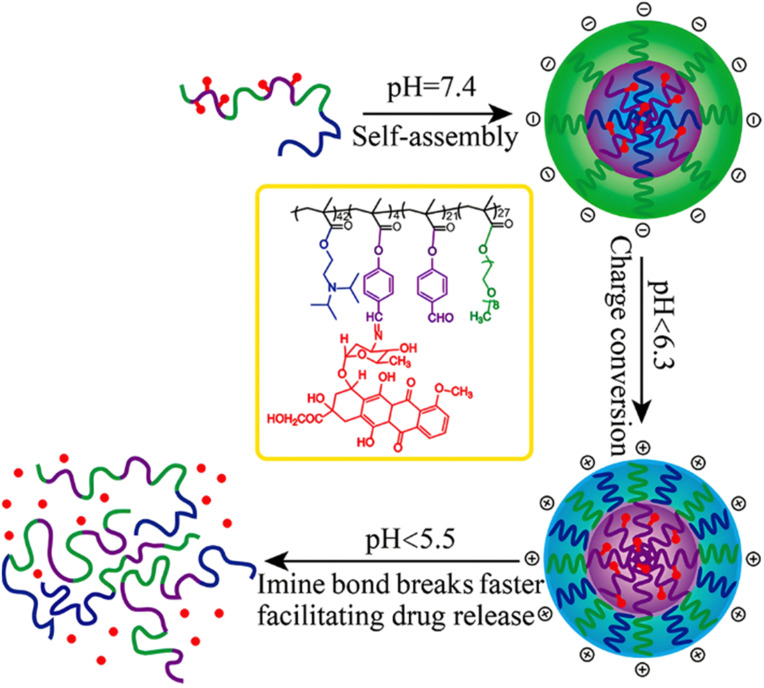
Structure of dual pH-sensitive P(OEGMA-*co*-FPMA)-*b*-PDPA diblock copolymer prodrug micelles obtained by RAFT polymerization and Dox conjugation *via* imine bond formation, and influence of the pH over micelle disassembly and Dox release. Adapted from ref. 126.

Interestingly, polymer prodrugs from RDRP can also combine two pH-sensitive modalities to better control the drug release kinetics and thus the therapeutic efficacy. This strategy seems particularly useful in cancer therapy, where the pH difference between cancer cells and healthy cells remains very small and difficult to exploit.^54,114^ Such system can be obtained from a pH-sensitive poly(oligo(ethylene glycol)methyl ether methacrylate)-*co*-4-formylphenyl methacrylate)-*block*-poly(2-(diisopropylamino)ethyl methacrylate) (P(OEGMA-*co*-FPMA)-*b*-PDPA) diblock copolymer *via* RAFT polymerization and further conjugated to Dox *via* imine bond formation through the aldehyde groups of FPMA.^126^ Micelles of 54 nm in diameter were formed and exhibited two pH sensitivities: (i) the protonation of the PDPA block at pH < 6.3 that resulted in a charge conversion causing micelles disassembly and (ii) hydrolysis of the imine groups that led to Dox release at pH < 5.5 (Fig. 10). Such dual pH-responsiveness led to a greater Dox release after 4 h at pH 5.5 (∼80%) than at pH 6.5 (∼40%) and at pH 7.4 (∼10%). They also exhibited significant cell internalization into HeLa cells at pH 5.5 compared to pH 6.5 (which was already greater than at pH 7.4), whereas the Dox-free micelles showed good cytocompatibility up to 10 mg mL^−1^. Other studies have reported dual pH-sensitive polymer prodrugs for the delivery of Dox using, for instance, β-cyclodextrin-*star*-poly(2-(diethylamino)ethyl methacrylate-*co*-4-formylphenyl methacrylate)-*b*-poly(oligo(ethylene glycol) methyl ether methacrylate) (β-CD-*star*-P(DEAEMA-*co*-FPMA)-*b*-POEGMA) star copolymers.^127^ These dual pH response systems offer interesting potential for the precise delivery of Dox to cancer cells, by conferring finer spatio-temporal control of drug release.

##### 3.1.1.3. Ester linker

The ester bond is also a widely-used drug linker to design polymer prodrugs by RDRP techniques (Fig. 1). It is usually obtained by condensation between hydroxyl and carboxylic acid groups and appeared to be a valuable polymer–drug linker because it can be cleaved by more than one stimuli including different pH conditions (acid and basic^128^), metal ions *via* hydrolytic degradation,^129^ or also the action of enzymes such as esterases and acid hydrolases.^130,131^ Such sensitivity to different stimuli may allow better control of drug release kinetics without complicating prodrug synthesis. Similarly to what has been shown with hydrazone and imine linkers, the acid-sensitivity can be selectively triggered when the polymer prodrugs reach specific cell compartments such as lysosomes or endosomes, where it is also frequent to find a variety of enzymes (as acid hydrolases and esterases) that could enhance intracellular ester bond cleavage. In addition, ester bonds are suitable for basic-catalyzed hydrolysis that could be relevant for achieving drug delivery to subcellular compartments such as mitochondria or peroxisomes, where the pH is slightly basic (∼8–9).^132^

**Fig. 11:**
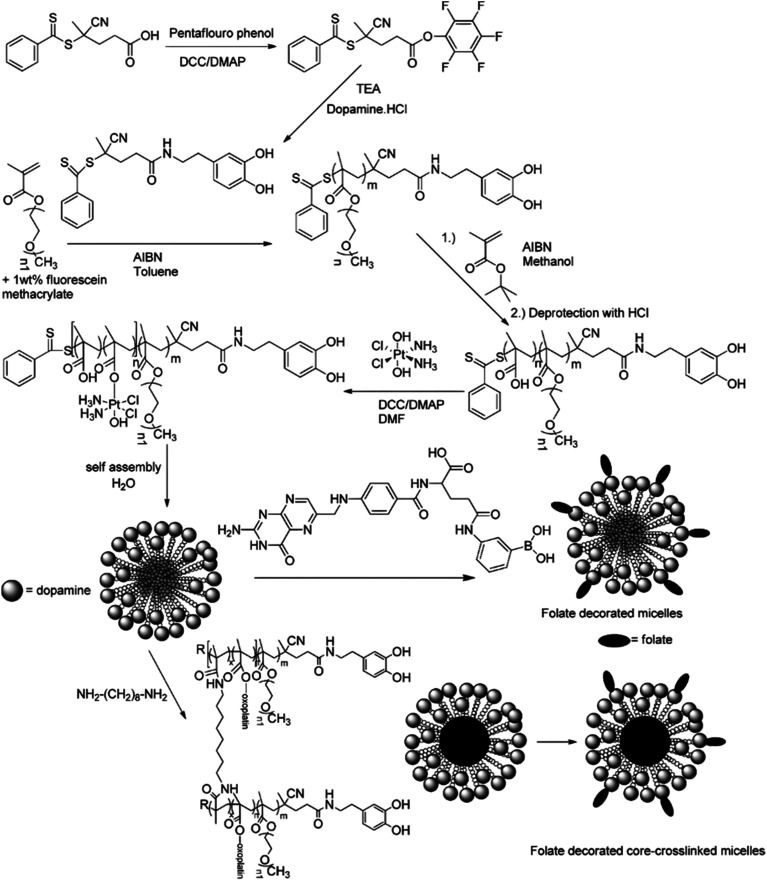
Synthesis of POEGMA-*b*-PMAA diblock copolymer from dopamine-terminated RAFT agent, followed by conjugation of oxoplatin and self-assembly into micelles, which were further stabilized by using a diamine cross-linker and surface-functionalized with folic acid. Adapted from ref. 133.

A typical example of platinum drug conjugation *via* ester drug linkage was reported by Stenzel and co-workers.^133^ They developed oxoplatin-functionalized poly(oligo(ethylene glycol) methyl ether methacrylate)-*block*-poly(methacrylic acid) (POEGMA-*b*-PMAA) diblock copolymer micelles by RAFT polymerization from a dopamine-derived RAFT agent for further conjugation to FA as targeting moiety for folate receptors (FR) (Fig. 11). Oxoplatin conjugation was performed on MAA units *via* carbodiimide coupling chemistry, leading to one grafted oxoplatin molecule every 7–11 MAA unit. The micelle colloidal stability was also enhanced *via* the use of a diamine cross-linker to react with free carboxylic acid groups of MAA units. The drug conjugation-induced self-assembly led to stable micelles exhibiting different average diameters as function of the PMAA block length. Drug release studies under reducing and acidic conditions (using ascorbic acid 7.5 mM and pH 5) showed reduction of oxoplatin to the platinum(ii) complex, followed by gradual release of cisplatin. Release of oxoplatin also led to micelle disassembly due to the formation of a water-soluble PMAA block, which should induce better clearance of the polymer when administered *in vivo*. Interestingly, the largest micelles showed the highest cytotoxicity on OVCAR-3 (FR+) cells but not on A549 (FR−) cells, demonstrating the targeting efficiency.

**Fig. 12:**
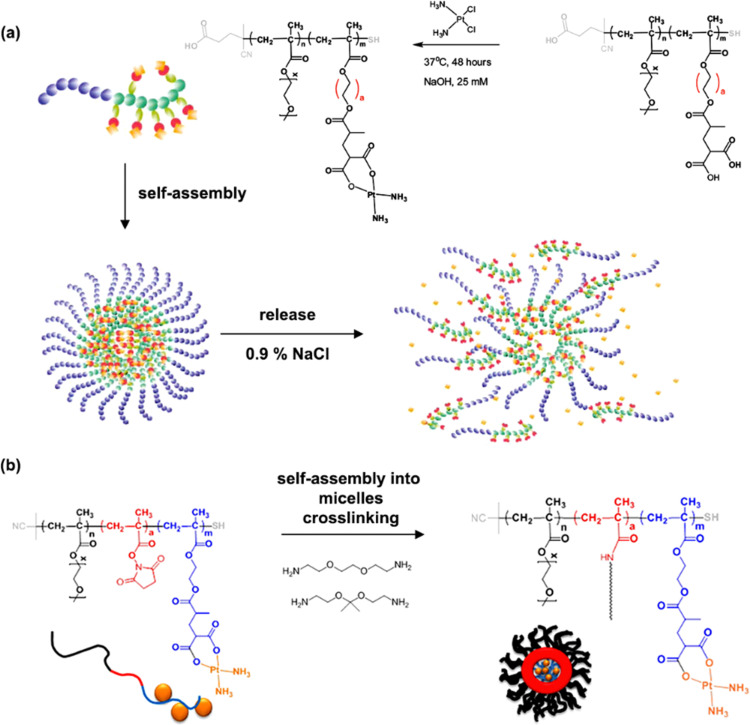
(a) Formation of polymer micelles by conjugation of POEGMA-*b*-PMAETC diblock copolymers to *cis*-diamminediaquaplatinum(ii) (CDDP); (b) formation of degradable, pH-sensitive and crosslinked polymer micelles by conjugation of POEGMA-*b*-PMANHS-*b*-PMAETC triblock copolymers to CDDP using (ketal) diamine linkers. Adapted from ref. 134 and 135.

The same group reported another synthetic strategy to produce ester linker-containing polymer prodrug micelles for cisplatin delivery, *via* the use of methacrylate monomers with 1,3-dicarboxylate functional groups as bifunctional chelator for platinum drugs.^134^ Sequential RAFT polymerization of OEGMA and 1,1-di-*tert*-butyl 3-(2-(methacryloyloxy)ethyl)butane-1,1,3-tricarboxylate (MAETC) with varying spacer lengths gave diblock copolymers that were then conjugated to *cis*-diamminediaquaplatinum(ii) (CDDP), resulting in amphiphilic copolymer prodrugs which formed micelles in aqueous solution (Fig. 12a). Interestingly, increasing the length of the spacer improved the colloidal stability of the micelles without impacting on drug release kinetics. This cisplatin delivery system can also be made sensitive to pH by crosslinking it with either permanent or pH-sensitive ketal diamine crosslinkers,^135^ with the aim of improving its colloidal stability, enabling better cellular uptake and higher cytotoxicity (Fig. 12b).^18^ The synthesis strategy relied on synthesis of poly(oligo(ethylene glycol)methylether methacrylate)-*block*-poly(*N*-hyroxysuccinic methacrylate)-*block*-poly(1,1-di-*tert*-butyl 3-(2-(methacryloyloxy)ethyl)butane-1,1,3-tricarboxylate) (POEGMA-*b*-PMANHS-*b*-PMAETC) triblock copolymers by RAFT polymerization, followed by deprotection of the carboxylic groups and complexation with CDDP. Self-assembly of the copolymer prodrugs in water gave 90 nm diameter micelles, which were then cross-linked by ketal diamine linkers, by reaction with the activated ester groups of the pendant *N*-succinimidyl units. The acid-sensitivity and degradability of the micelles were demonstrated after incubation at pH 5.5 for 72 h, leading to the formation of free unimers. CDDP was released in the presence of NaCl to promote ligand exchange with the carboxylate groups conjugated to the drug. It was shown that the amount of released CDDP at pH 5.5 for the acid-cleavable crosslinked micelles was twice as much as that at pH 7.4 demonstrating accelerated acidic pH-driven drug release in conditions close to tumoral environment, whereas the pH value did not have an effect for the non-crosslinked counterparts. The acid-cleavable crosslinked micelles also showed superior cytotoxicity against OVCAR-3 cells compared to uncross-linked micelles due to a greater cellular uptake, but also to a faster drug action in comparison to permanently cross-linked micelles.^134,135^

Another system for cisplatin delivery was obtained from a poly(oligo(ethylene glycol) methyl ether acrylate)-*block*-poly(glycidyl azide)-*block*-poly(oligo(ethylene glycol)methyl ether acrylate) (POEGA-*b*-PGAP-*b*-POEGA) triblock copolymer obtained by divergent ATRP of OEGA from a difunctional PGAP macroinitiator. The pendant azide groups from PGAP were then reduced into amines to install bidentate carboxylate moieties *via* consecutive amidation and thiol–ene reactions. After complexation with cisplatin, the polymer–cisplatin prodrugs were able to self-assemble into stable micelles of 142 nm. The release of cisplatin was monitored at pH 5.6, leading to 60% release after 20 h and *in vitro* studies on MCF-7 cell line confirmed their cytotoxicity, whereas the drug-free copolymer remained cytocompatible.^136^

**Fig. 13:**
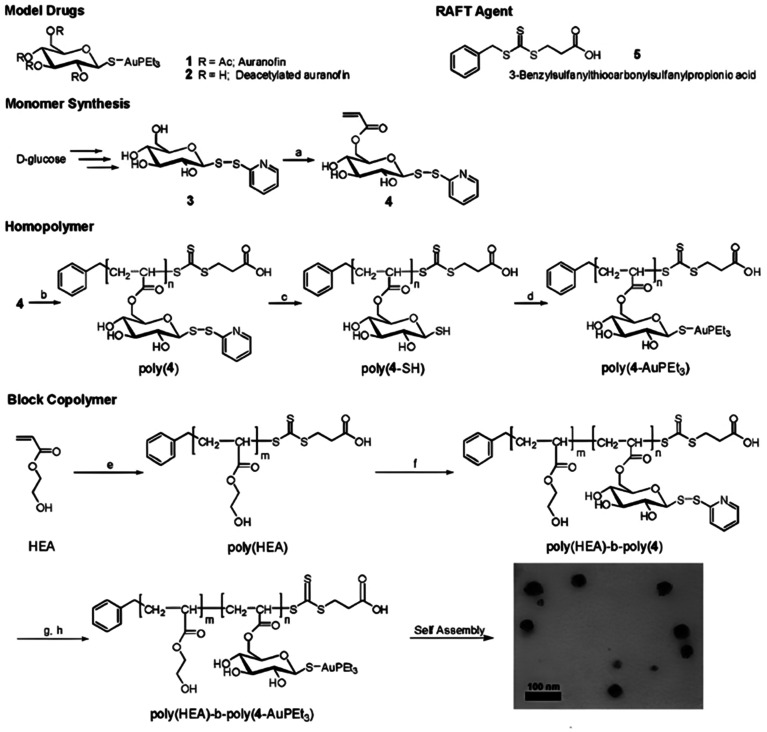
Synthesis of deacetylated auranofin-based polymer prodrug micelles by RAFT polymerization and AuPEt_3_Cl conjugation. Adapted from ref. 137.

The synthesis of polymer prodrugs based on gold-based metallodrugs through ester bond linkages has also been reported. This was achieved by RAFT polymerization of a protected thiosugar moiety-bearing glycomonomer from a poly(2-hydroxyethyl acrylate) (PHEA) macro RAFT agent (Fig. 13).^137^ The obtained diblock copolymer was then functionalized with AuPEt_3_Cl with a coupling efficiency of ∼72% to give the deacetylated structure of auranofin, a gold(i) complex which has extensively been used to treat rheumatoid arthritis. The amphiphilic copolymer prodrugs gave micelles of 75 nm in diameter with greater cytotoxicity against OVCAR-3 human ovarian carcinoma cells than free auranofin.

##### 3.1.1.4. Other pH-sensitive linkers

**Fig. 14:**
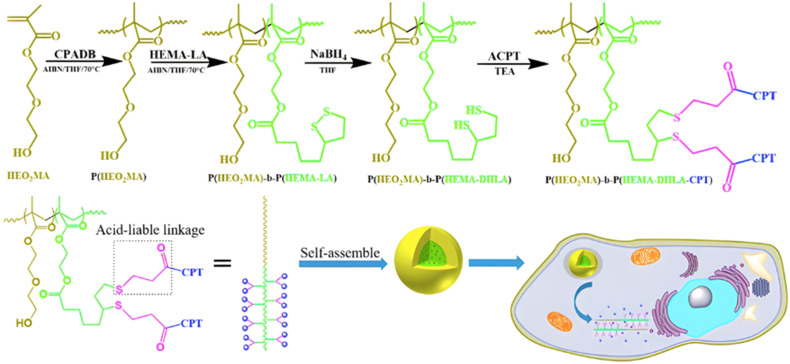
Synthesis route of P(HEO_2_MA)-*b*-P(HEMA-DHLA-βthiopro-Cpt) polymer prodrug and fabrication of pH-responsive nanoparticles for drug delivery. Adapted from ref. 141.

Although Schiff bases and ester bonds represent the most common pH-sensitive drug linkers in the design of polymer prodrugs by RDRP, other functional groups that can be cleaved by acid-sensitive hydrolysis have been reported (Fig. 1). Such a diversity originates from the compatibility of RDRP techniques with a broad range of functional groups and organic coupling reactions, leading to drug linkers exhibiting different chemical structures, stability and lability. For instance, RDRP has been applied to the synthesis of polymer prodrugs based on the thiopropionate linker which can be easily hydrolyzed under mildly acidic conditions in endosomes (Fig. 1).^138,139^ The pH-sensitivity of the thiopropionate bond has been attributed to the formation of a partial positive charge on the ester carbonyl-linked carbon due to an inductive effect from the sulfur atom.^140^ As for polymer prodrugs, a poly(2-(2-hydroethoxy)ethyl methacrylate)-*block*-poly(2-hydroxyethyl methacrylate-dihydrolipoic acid) P(HEO_2_MA)-*b*-P(HEMA-DHLA) diblock copolymer prepared by RAFT polymerization was functionalized with acrylate-bearing anticancer camptothecin (Cpt) *via* the formation of a β-thiopropionate (βthiopro) bond through Michael addition reaction on the two thiol groups of DHLA moieties (Fig. 14).^141^ The coupling of hydrophobic Cpt moieties enabled the resulting amphiphilic polymer prodrug to self-assemble into nanoparticles, which achieved 80% Cpt release at pH 5 after 96 h. *In vitro* evaluation on HeLa cells showed similar cytotoxicity than the free Cpt, which was associated to early-induced apoptosis. The polymer prodrug nanoparticles were administered to 7-week-old tumor-bearing CD-1 mice, which resulted in tumor growth suppression with a size stabilized around 70 mm^3^ after 12 days post-treatment, whereas a rapid tumor growth, from 50 to 326 mm^3^, was obtained without treatment.

#### 3.1.2. Redox-sensitivity

Even if pH-sensitivity was successfully demonstrated *in vitro* on various types of polymer prodrug nanocarriers as a promising strategy to perform spatio-temporal drug release, achieving such selective drug delivery *in vivo* by taking advantage of the small pH difference with the tumor microenvironment^54,58,114^ is much more challenging.^142,143^ Therefore, other relevant stimuli related to cancer cells,^48^ such as the hypoxia-specific environment,^57^ could constitute an interesting alternative. The presence of hypoxic cells in solid tumors is a specific feature that stimuli-sensitive nanocarriers could take advantage of to trigger drug release,^55^*via* the design of reducible drug linkers.

**Fig. 15:**
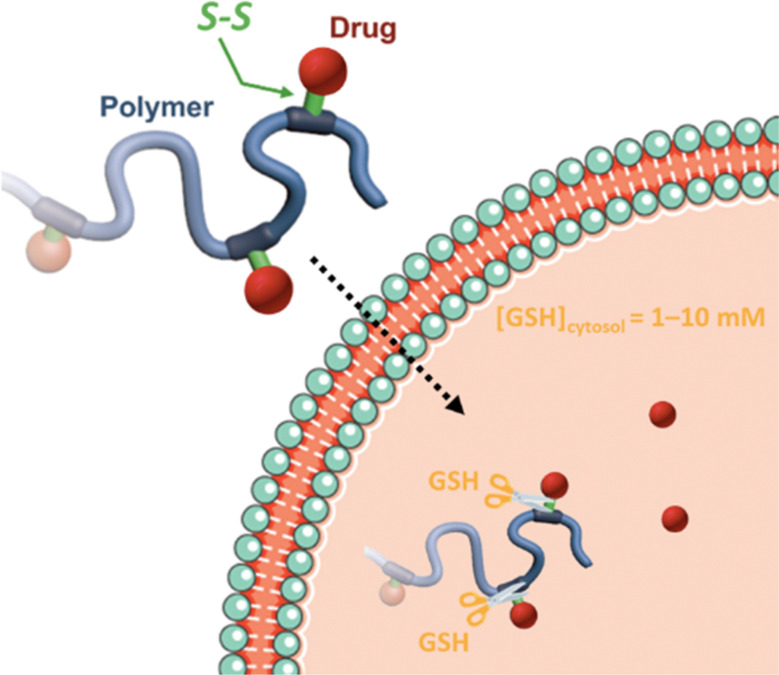
Intracellular degradation of disulfide linkers in polymer prodrugs by glutathione (GSH).

Disulfide bonds are the most commonly used reducible linkers in polymer prodrug nanocarriers from RDRP (Fig. 1), to achieve drug release upon internalization into the intracellular environment of the cancer cells. The disulfide bonds can be cleaved by electrochemical reduction induced, for instance, by reducing agents such as glutathione (GSH) or during thiol-disulfide exchange reactions.^144^ They are also relatively stable at physiological pH and are mainly reduced in the cytosol, which contains high thiol concentrations notably due to a high presence of GSH (Fig. 15). Interestingly, the relatively higher stability of disulfide bonds in biological fluids compared to pH-sensitive bonds could be advantageous for more controlled and effective delivery.^145^

RAFT polymerization has been used to prepare disulfide-containing vorinostat-conjugated diblock copolymer micelles loaded with tamoxifen for combination therapy. The diblock copolymer consists of a POEGMA block connected to a polystyrene (PS) block to which vorinostat was conjugated on the para position of the styrene units *via* disulfide linkages. Tamoxifen was physically encapsulated during the copolymer self-assembly into micelles (31 nm). Both vorinostat and tamoxifen release were shown to be dependent on GSH concentration (vorinostat: >70% release after 12 h; tamoxifen: 40% of release after 48 h using 10 mM GSH). This proved that the reducing environment was capable of inducing the release of vorinostat by cleavage of the disulfide bonds leading to micelle disassembly and release of tamoxifen. Importantly, cell viability experiments on TNBC cells showed a synergistic effect of the two drugs.^146^

**Fig. 16:**
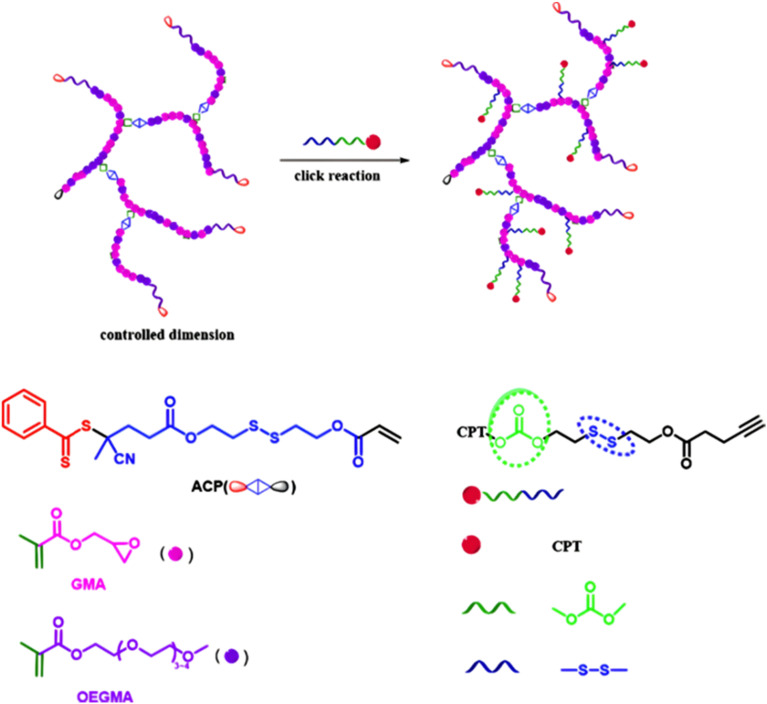
Synthesis of hyperbranched h-P(GMA-*co*-OEGMA)-*b*-POEGMA polymer prodrug of Cpt by RAFT polymerization and click chemistry, enabling the insertion of a dual-stimuli (*i.e.*, redox and pH) sensitive linker. Adapted from ref. 147.

The combination of redox- and pH-sensitivities into a single drug delivery system (referred to as dual stimuli-sensitive nanomedicines),^11^ has been widely studied to improve the spatio-temporal selectivity of drug release. From a design point of view, dual-sensitivity could be conferred either by a drug linkage integrating different chemical groups sensitive to pH and a reductive environment, or by a combination of a single stimulus-sensitive linkage and a stimulus-sensitive polymer. A typical example are RAFT-synthesized h-P(GMA-*co*-OEGMA)-*b*-POEGMA hyperbranched diblock copolymer prodrugs based on dual-responsive linkers sensitive to both pH and reductive conditions, for the delivery of Cpt (Fig. 16).^147^ The hyperbranched topology was achieved by using a chain transfer monomer, 2-((2-(acryloyl oxy)ethyl)disulfanyl)ethyl 4-cyano4-(phenylcarbonothioylthio)pentanoate (ACP), allowing a well-controlled number of disulfide linkages in each branching point. Conjugation of Cpt was performed by click chemistry after ring-opening of GMA units using sodium azide reaction leading to alkyne-functionalized Cpt-based linker containing carbonate and disulfide moieties. The combination of acidic medium (pH 5) and intracellular reductive GSH (10 mM) led to a 10-fold higher Cpt release compared to physiological conditions after 96 h (less than <5%). This dual-sensitive nanocarrier also led to a quicker drug release (45% after 24 h) compared to previously reported Cpt-polymer conjugates based on a very similar structure and topology but lacking pH-sensitivity.^148^*In vitro* evaluations showed significant cytotoxicity of the polymer prodrugs on HeLa cells even if the obtained IC_50_ was higher than that of the free drug, probably due to a slower internalization mechanism (endocytosis) and to the time required to release the drug.

The same combination of stimuli was also used to develop theranostic polymer prodrugs for cancer therapy.^149^ A random copolymer based on 2-(dimethylamino)ethyl methacrylate (DEAEMA), 4-(diphenylamino)benzyl methacrylate (TPMA) and a methyl methacrylate monomer functionalized with a *p*-nitrophenyl ester *via* a disulfide bond (MMA-*SS*-NO_2_) was designed by RAFT polymerization. It was then chain extended by MPC prior to gemcitabine (Gem) functionalization to give a PMPC-*b*-P(DEAEMA-*co*-MMA-*SS*-GEM-*co*-TPMA) copolymer prodrug with a drug loading of 8.8 wt%. The obtained micelles exhibited a mean diameter of 53 nm, which rapidly increased at pH 5 (which was attributed to hydrophobic-to-hydrophilic change of the PDEAEMA block in addition to protonation of tertiary amino groups in the PMPC block), whereas it stayed constant at pH 6 and physiological pH thanks to effective stabilization by the zwitterionic PMPC shell. Importantly, acidic (pH 5) and GSH-concentrated (10 mM) conditions resulted in high Gem release (∼95% after 48 h), compared with only 10% at pH 7.4, highlighting the benefits of combining two stimuli to enhance drug release efficiency. This system has also been equipped with an aggregation-induced emission (AIE) behavior and a two-photon capability, enabling potential use for two-photon cell imaging and deep tissue imaging. Considerable cytotoxicity was shown on 4T1 cells and *in vivo* antitumor efficacy also assessed a higher tumor inhibition and less focal necrosis, liver and spleen inflammation compared to the use of free Gem, making this system promising for both cancer treatment and diagnosis.

#### 3.1.3. Enzyme-sensitivity

Pathophysiological conditions associated with cancer are also characterized by the presence of certain specific enzymes and/or abnormal levels of enzyme expression,^58^ which stimuli-sensitive polymer prodrugs can also take advantage of. An interesting strategy, therefore, is to use the flexibility of RDRP to design polymer prodrugs incorporating enzymatically cleavable drug linkages. In this context, common families of enzymes have been often targeted as they are specific to tumor activities, as demonstrated for the proteases^150–153^ and hydrolases^130,131,154^ superfamilies. For example, a well-known and often targeted subfamily of hydrolases are esterases. Different types of esterases exist and differ in their biological targets and functions, as well as in a variable level of expression that could be dysregulated in the tumoral environment.^131,154^ It has also been reported that the stereoselectivity of some esterases can be affected in cancer cells, resulting in preferential hydrolysis of ester bonds.^155^ Thus, hydrolysis of ester bonds appears to be a relevant tumor-targeting strategy *via* the design of ester linker-containing polymer prodrugs. The family of proteases is also often targeted when designing drug delivery systems. For example, cathepsin B-derived proteases are interesting targets for drug linkers because they could be used both as a tumor biomarker and for enzymatic cleavage activity.^150,156,157^ Another major protease known for its involvement in tumor-associated mechanisms belongs to the family of extracellular matrix mellatoproteinases that have been implicated not only in cancer cell migration, but also in the regulation of cell growth and angiogenesis.^158^ Proteases are able to hydrolyze amide bonds that are introduced in drug-based conjugates using peptide units as linkers^159,160^ that represent another interesting enzyme-based strategy to design sensitive drug linkers.

##### 3.1.3.1. Esterases: ester and β-thioester bond hydrolysis

The diversity of esterases (*e.g.*, carboxylesterase, acetylcholine esterase) makes possible to trigger drug release by targeting different enzymatic pathways, such as: (i) direct drug delivery from the polymer nanocarrier *via* site-specific enzymatic cleavage or (ii) enzymatic degradation of the drug carrier itself, resulting in better exposure of the drug which will facilitate the cleavage of the polymer–drug linker.^161^ This last strategy could be employed to increase the specificity of the drug delivery by achieving active targeting.

**Fig. 17:**
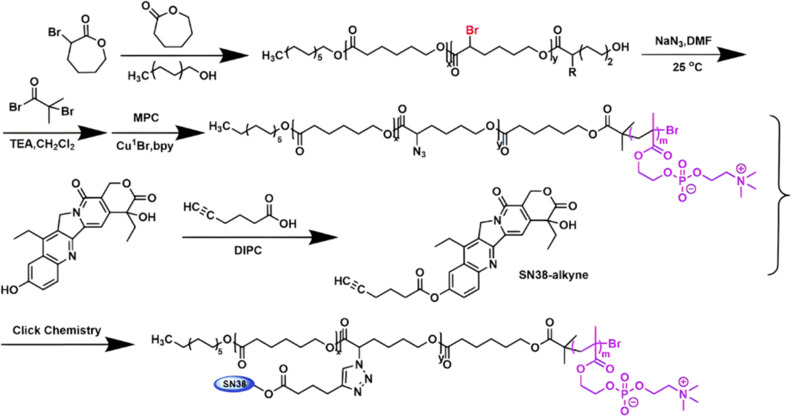
Synthetic route to P(ACL-*co*-CL)-*b*-PMPC copolymer by ROP and ATRP, followed by coupling of SN-38 by click chemistry. Adapted from ref. 162.

Several polymer prodrugs sensitive to esterases have been synthesized by RDRP (Fig. 1). For example, poly(α-azide caprolactone-*co*-caprolactone)-*b*-poly(2-methacryloyloxyethyl phosphorylcholine) (P(ACL-*co*-CL)-*b*-PMPC) was obtained by sequential ROP of ACL/CL and ATRP of MPC, followed by the side-chain coupling of the alkyne-bearing derivative of Cpt (SN-38) by click chemistry, leading to a drug content of ∼10 mol% (Fig. 17).^162^ P(CL/CL-*g*-SN38)-PMPC micelles of 196 and 237 nm with a drug loading of 12.7% exhibited 70% of SN-38 release after 70 h in presence of pig liver esterase. Interestingly, the drug linkage remained stable into the bloodstream before reaching the cytosol and the lysosomes of cancer cells. The micelles were also evaluated *in vitro* on two breast cancer cell lines (MCF-7 and 4T1) leading to a significant cytotoxicity.

**Fig. 18:**
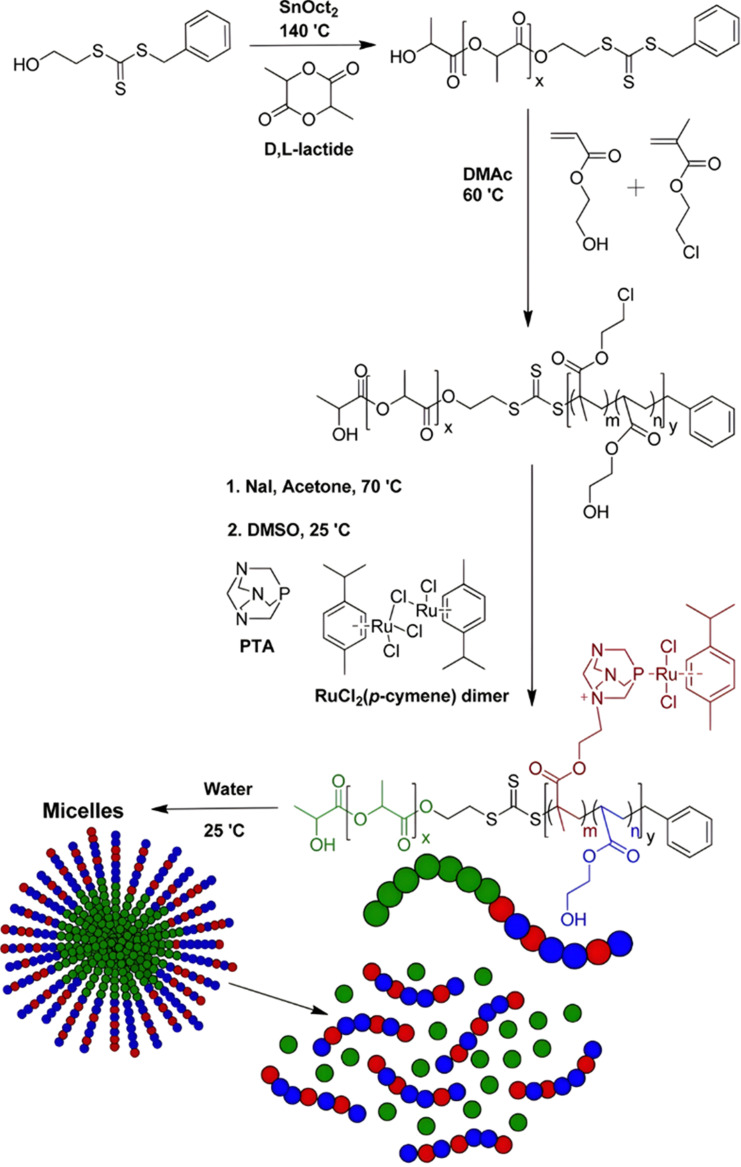
Synthesis of PLA-*b*-P(HEA-*co*-CEMA) diblock copolymer and subsequent functionalization with RAPTA-C, micellization and degradation. Adapted from ref. 163.

Esterases can also be used to degrade not the polymer–drug linker itself, but the polymer nanocarrier used to protect the drug from early degradation and to better expose it once the site of action is reached for improved therapeutic effect. This has been achieved with poly(lactide)-*block*-poly(2-hydroxyethyl acrylate-*co*-2-chloroethyl methacrylate) (PLA-*b*-P(HEA-*co*-CEMA)) diblock copolymers obtained by sequential ROP and RAFT polymerizations (Fig. 18), which were functionalized with a ruthenium-based metallodrug (RAPTA-C), known to be highly toxic *in vitro* and selective for metastases *in vivo*.^163^ Self-assembly of the copolymer prodrugs led to 250 nm – micelles exhibiting RAPTA-C drug moieties at their periphery. They showed complete disassembly when incubated with hydrolases for 2 days at 37 °C, mediated by the degradation of the PLA blocks. The micelles demonstrated a 10-fold increase in cytotoxicity on three ovarian cancer cell lines (A2780, A2780cis, and Ovcar-3) when compared with free RAPTA-C.

**Fig. 19:**
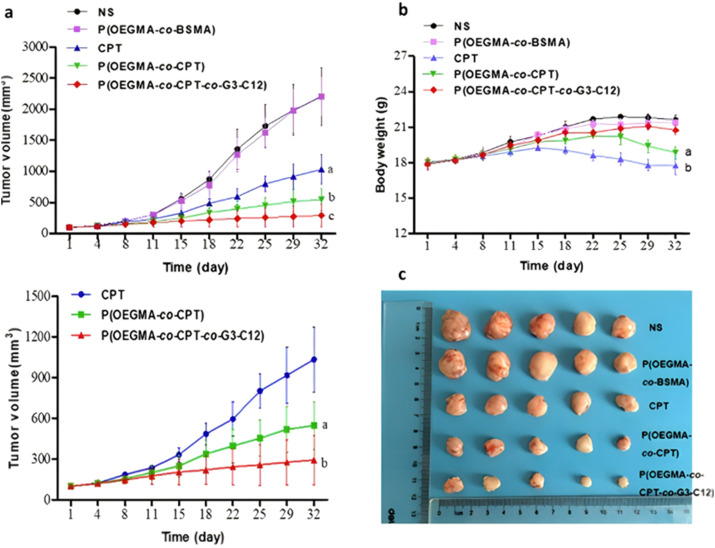
*In vivo* antitumor efficacy in DU145-bearing mice treated with free Cpt, P(OEGMA-*co*-BSMA), P(OEGMA-*co*-BSMA-*ester*-Cpt) and P(OEGMA-*co*-BSMA-*ester*-Cpt-*co*-G3-C12) nanoparticles. (a) Evolution of the tumor growth in the 0–3 000 mm^3^ range with time (up) and zoom in the 0–1 500 mm^3^ range (down); (b) evolution of the body weight with time; (c) images of excised tumors at the end of the treatment (*i.e.*, day 32). Adapted from ref. 166.

However, one has to bear in mind that the abundant presence of esterases or hydrolases in the whole biological environment^130^ (not only in tumor cells) could make this general strategy less selective *in vivo*, and may require the addition of active targeting ligands. This is the case of a RAFT-synthesized copolymer designed to target the galectin-3 receptor (which is overexpressed in prostate cancer cells^164,165^), composed of OEGMA and 3-((2-(methacryloyloxy)ethyl)thio)propanoic acid (BSMA) monomer units, onto which Cpt (drug loading 11.3 wt%) and the targeting G3-C12 peptide have been grafted *via* esterification and amidation, respectively.^166^ Incubation of the resultant P(OEGMA-*co*-BSMA-*ester*-Cpt-*co*-G3-C12) 70 nm-nanoparticles with esterases triggered 77% of drug release after 24 h, likely due to the cleavage of esterase-sensitive β-thioester bond between the polymer and Cpt. Such nanoparticles showed higher cytotoxicity on DU145 prostate cancer cells, greater cellular uptake and better anticancer efficacy on tumor-bearing mice compared with the non-targeted nanoparticles or with the free drug (Fig. 19).

**Fig. 20:**
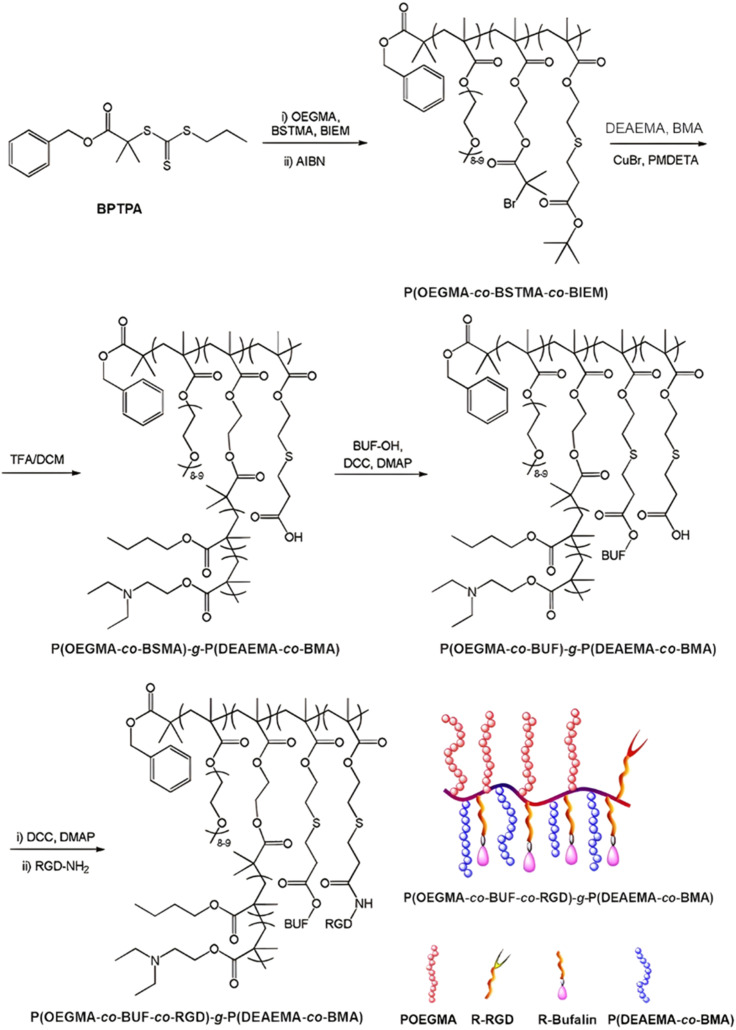
Synthesis route for P(OEGMA-*co*-BSMA-ester-BUF-*co*-RGD)-*g*-P(DEAEMA-*co*-BMA) prodrug by a combination of ATRP and RAFT polymerizations. Adapted from ref. 169.

The same design strategy has been applied to the anticancer drug bufalin (Buf) and two targeting ligands: (i) the octreotide peptide, to target somatostatin receptors overexpressed in breast cancer cells^167^ and (ii) the arginylglycylaspartic acid peptide (RGD), to improve cancer cell penetration.^168^ In both cases, the sensitivity of esterases to β-thioester bonds and the beneficial effect of the targeting ligands were demonstrated *in vitro* and *in vivo*. Such Buf-based system has been further supplemented by an endosomal escape capability *via* the design of brush-type polymer prodrug nanocarriers.^169^ This was achieved by the RAFT terpolymerization of OEGMA, protected BSMA and 2-(2-bromoisobutyryloxy)ethyl methacrylate (BIEM) as a ATRP initiator, followed by synthesis of poly(*N*,*N*-diethylaminoethyl methacrylate-*co*-butyl methacrylate (P(DEAEMA-*co*-BMA)) side brushes by ATRP from pendant BIEM moieties. After deprotection of BSMA groups, the resulting P(OEGMA-*co*-BSTMA)-*g*-P(DEAEMA-*co*-BMA) copolymer brushes were conjugated to Buf and to the RGD peptide (Fig. 20) and self-assembled into nanoparticles of 148 nm. The Buf release was significant in presence of esterases at pH 7 and, to a lower extent, at pH 5 without esterases. Interestingly, the presence of esterases at pH 5 did not result in greater release of Buf than at pH 7, probably due to a reduced enzymatic activity under acidic conditions. Although the targeted nanoparticles demonstrated higher cytotoxicity against colorectal cancer cells HCT116 compared with the non-targeted nanoparticles (IC_50_ = 10 and 80 nM, respectively), the beneficial effect of the RGD peptide *in vivo* was less marked. Nonetheless, histological and immunochemical analyses showed improved cell apoptosis, angiogenesis inhibition and anti-proliferation effect compared to the free drug.

##### 3.1.3.2. Proteases: Gly–Phe–Lys–Gly (GFLG) peptide linker

Proteases are another large family of enzymes that can be targeted for the selective cleavage of polymer–drug linkers, due to their abundance in the body^170^ and their involvement in numerous biological phenomena such as cell differentiation^171^ or angiogenesis.^172^ By hydrolyzing amide bonds, protease activity are essential in normal physiology but can also been dysregulated and implicated in the development of tumors.^150,151,173^

**Fig. 21:**
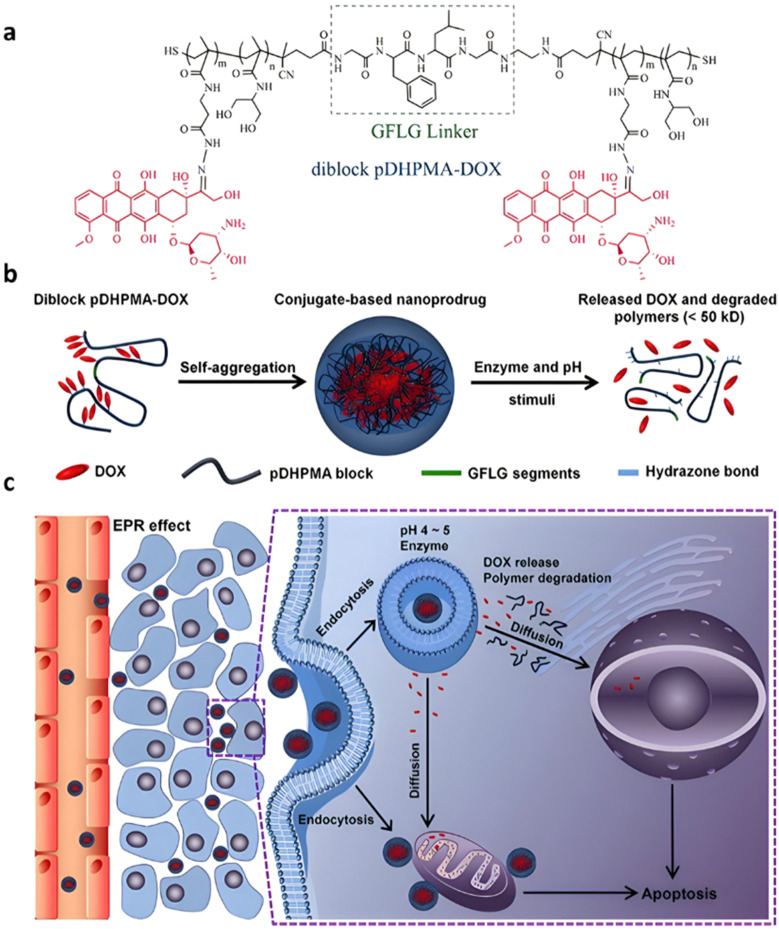
(a) Synthesis of pDHPMA-Dox polymer prodrugs; (b) self-assembly of pDHPMA-Dox polymer prodrugs into nanoparticles and stimuli-driven (*i.e.*, acid pH and enzyme) drug release; (c) suggested mechanism of drug delivery to cancer cells *via* passive targeting. Adapted from ref. 175.

Even if not directly connected to the drug, inserting peptide sequences into polymer structures is a promising approach for generating protease-sensitive polymer prodrugs by RDRP. A typical example is to use the Glycine-Phenylalanine-Leucine-Glycine (GFLG) peptide (Fig. 1), which is sensitive to the protease cathepsin B and remains stable in plasma,^174^ allowing cleavage after endocytosis. One clever strategy for inserting the GFLG peptide into RDRP polymer prodrugs is to use a difunctional GFLG-based RAFT agent, which ensures the presence of the peptide in the middle of the polymer backbone after divergent RAFT polymerization.^175^ This was applied to the synthesis of (poly(*N*-(1,3-dihydroxypropan-2-yl) methacrylamide)-*co*-methacrylamide-*hyd*-Dox)_2_-GFLG polymer prodrugs (Fig. 21a), which self-assembled into micelles of 21 nm (Fig. 21b). They exhibited selective drug release in response to tumor microenvironmental pH and enzymatic degradation into smaller fragments due to the abnormally high concentration of cathepsin B (Fig. 21c). *In vivo* experiments in BALB/c mice with 4T1 xenografted tumors showed a significant increase in blood circulation time for the Dox micelles as well as improvement in tumor growth inhibition compared with free Dox (54% and 27%, respectively).

Another approach to insert the GFLG peptide sequence into polymer prodrugs from RDRP is through the design of linear-dendritic block copolymers.^176^ This was achieved by RAFT polymerization of dendron-based (of variable valency, *n* = 1, 2 or 4) GFLG-methacrylamide monomers from a POEGMA macro-RAFT agent. The polymer prodrugs formed ∼50 nm micelles capable of encapsulating chlorin e6 as a photosensitizer, and whose degradation specifically triggered the intracellular release of Dox and Ce6 in the tumor microenvironment. This enabled the possibility of a combined therapy that suppressed tumor growth in 4T1 tumor-bearing mice.

### 3.2. The combination of endogenous and exogenous stimuli

Although the tumor microenvironment provides access to different stimuli (*e.g.*, acidic pH, higher concentration of certain enzymes, reducing environment) that can be exploited, polymer prodrugs nanocarriers from the “grafting to” approach have also been designed to be sensitive to both endogenous et exogenous stimuli (Table 2).^177^ This was achieved to improve their biological performances and tackle some potential limitations associated with the use of one unique stimulus (*e.g.*, lack of selectivity).

**Fig. 22:**
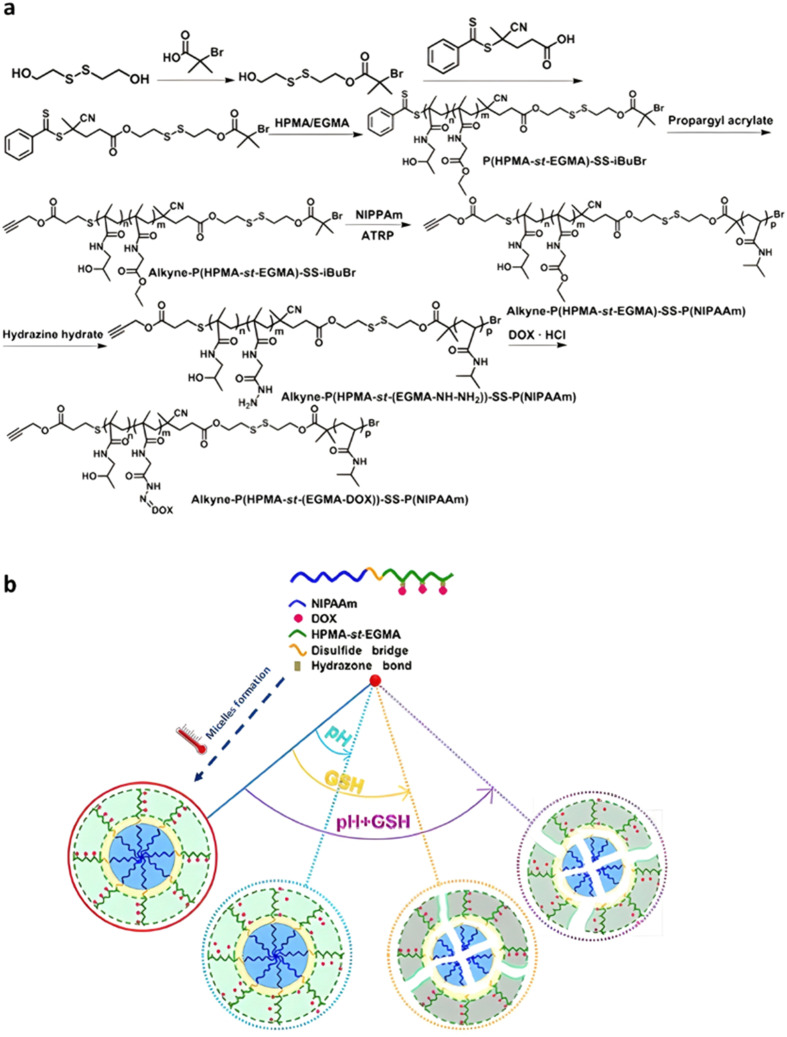
(a) Synthesis of alkyne-P(HPMA-*st*-EGMA-*hyd*-Dox)-*SS*-PNIPAAm copolymer prodrugs; (b) thermo-driven polymer prodrug micelle formation and behavior as function of pH and reductive GSH. Adapted from ref. 179.

Temperature is one of the key exogenous stimuli which has been incorporated into stimuli-sensitive prodrugs *via* the use of thermosensitive polymers exhibiting lower critical solution temperature (LCST) behavior, such as poly(*N*-isopropylacrylamide) (PNIPAAm).^178^ A polymer prodrug sensitive to pH, reducing environment and temperature, was designed from a ATRP-RAFT dual functional initiator/controlling agent containing a disulfide bond, 4-cyanopentanoic acid dithiobenzoate-*SS*-2-hydroxyethyl-2′-(bromoisobutyryl)ethyl disulfide (CPADB-*SS*-iBuBr).^179^ RAFT copolymerization of HPMA and ethyl glycinate methacrylamide (EGMA) was first carried out, leading to P(HPMA-*st*-EGMA)-*SS*-iBuBr, followed by the RAFT end group substitution by propargyl acrylate, and ATRP of NIPAAm (Fig. 22a). The resulting double hydrophilic alkyne-P(HPMA-*st*-EGMA)-*SS*-PNIPAAm diblock copolymer was then conjugated to Dox through acid-labile hydrazine linkage onto EGMA pendent units, achieving a drug loading of 13.7 wt%. Due to the LCST of the PNIPAAm block, the polymer prodrug was formulated into core–shell micelles of 180 nm *via* temperature-induced self-assembly around 36–42 °C. Higher Dox release was observed at pH 5.5 compared to pH 7.4 (69% *vs.* 40% after 48 h, respectively) at 37 °C, confirming the acid pH-triggered cleavage of hydrazone linkages and faster diffusion of Dox from the hydrophilic shell (Fig. 22b). The combination of acidic conditions and a reductive environment (GSH 10 mM) at 37 °C led to even faster Dox release in the first 12 h owing to cleavage of the disulfide bonds which led to micelle disassembly (Fig. 22b), reaching a plateau of 80% after 48 h. Unreleased Dox accounted for about 20% which could be attributed to partial co-aggregation of free Dox with the hydrophobic PNIPAAm core of the micelles. Interestingly, higher cumulative Dox release was observed in PBS (pH 7.4) at 25 °C and at 40 °C compared to 37 °C, probably due to the solubilization of micelles into unimers at 25 °C (below the LCST) and formation of aggregates at 40 °C, which destabilized the micelles initially obtained at 37 °C. Cell viability studies on HeLa cells demonstrated significant cytotoxicity notably due to the presence of disulfide bonds that could promote intracellular reductive-triggered micelle disassembly and drug release.

## 4. Linking drugs to the monomer prior to polymerization (grafting through)

The second synthetic strategy to produce polymer prodrugs by RDRP is based on the grafting of the drug onto a monomer (resulting in a prodrug monomer) prior to its polymerization, also called “grafting through” method (Fig. 3b). RDRP techniques allow for a wide range of monomers bearing functional groups to be polymerized in a controlled fashion with no or negligible side-reactions involving these chemical groups. This strategy allows to insert drug moieties at predefined ratios on a single polymer chain and to achieve higher drug loadings by avoiding steric hindrance during drug conjugation usually encountered with the “grafting to” method. The polymerization of prodrug monomers also provides greater flexibility in polymer architecture, notably by allowing the design of block copolymer prodrugs with well-defined polymer prodrug blocks.^47^ Representative examples of “grafting through” systems have been mostly obtained using ring-opening metathesis polymerization (ROMP),^180–184^ but other studies adapted this strategy to RDRP techniques such as ATRP and RAFT. Similarly to RDRP-derived polymer prodrugs obtained by the “grafting to” approach, the tumor microenvironment has been targeted by designing drug linkers sensitive to endogenous stimuli including the pH, the redox environment and the presence of specific enzymes (Table 3). The additional application of an external stimulus, such as the temperature or the light, may trigger more specifically the drug release and enhance its efficiency (see Table 4, Section 3.2).

**Table 3:** Stimuli-sensitive drug linkers in polymer prodrugs obtained by RDRP techniques *via* the “grafting through” strategy

Linker	Drug	Polymerization method	Polymer prodrug	Cleavage conditions	Release	Ref.
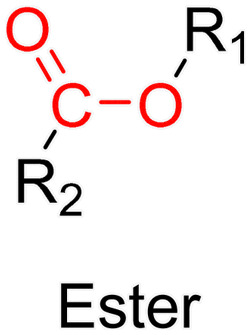	Dt	RAFT	P(SMA-*co*-OEGMA)	Human serum	50% after 384 h	185
			P(MAA-*co*-DMAEMA)-*b*-PSMA		50% after 240 h	
	POD	RAFT	PMA-*b*-PTEGMA	pH 5	52% after 72 h	188
	20 Cpt: aliphatic ester	RAFT	P(CBM-*co*-SMA)	Human serum	20 Cpt: 12% after 4 days	211
	10 Cpt: aromatic ester				10 Cpt: 37% after 4 days	
	Cpt	RAFT	H40-*star*-P(MA)-*b*-PMPC	pH	50% after 36 h	187
	Ibuprofen + Dox	ATRP	PEG-*b*-PHEMA	pH 5	55% after 10 h	189
	Mtx	RAFT	PHPMA + crosslinker: EGDMA	Esterases	No release	212
	PPMP + Dox	RAFT	POEGMA-*b*-PMA	PBS	10% after 2 h	215

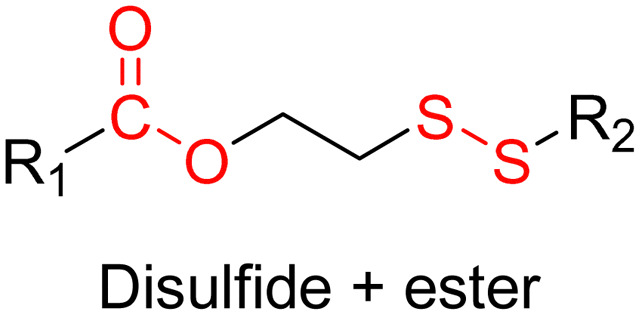	Cpt	RAFT	PEG-*b*-PHSEMA	—	—	195
	Cpt	RAFT	PEG-*b*-P(EO_2_MA-*co*-HSEMA) + cross-linkers: CBMA/BzMA	GSH 10 mM	42% after 48 h	196
	Cpt	RAFT	POEGMA-*b*-P(POEGMA-*co*-HSEMA) + cross-linkers: EGDMA/HPMA	GSH 10 mM	70% after 48 h	197
	Cpt	RAFT	h-P(HSEMA-*co*-GMA)-*b*-P(OEGMA-*co*-GPMAAm)	DTT 10 mM	60% after 24 h	147
	Cpt	ATRP	P(HEMA-*SS*-Cpt)-*b*-POEGMA	DTT 5 mM	90% after 24 h	203
	Cpt	ATRP	Dex-PHSEMA-*b*-POEGMA	DTT 10 mM	100% after 72 h	198
	Irinotecan	ATRP	α-CD-P(HSEMA-*co*-OEGMA)	GSH 10 mM	80% after 120 h	201
	Cpt	ATRP	α-CD-PEG-*b*-P(HSEMA-*b*-POEGMA	DTT 10 mM	80% after 24 h	200
	Cpt	RAFT	P(HSEMA-*co*-PEMA) + PEG-*b*-PCL	pH 6.8 + GSH 5 mM	50–80% after 50 h	209
	Cpt + Dox	ATRP	α-CD-P(HSEMA-*co*-OEGMA-*co*-DPA)	pH 5	Dox: 70% after 72 h	210
				GSH 10 mM	Cpt: 75% after 48 h	

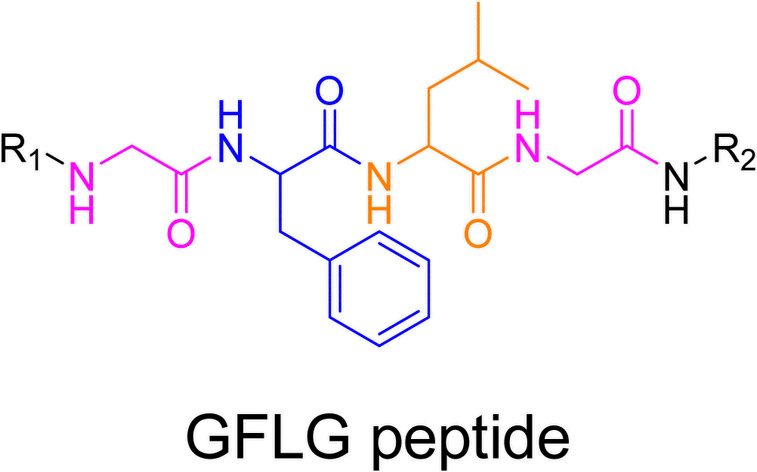	Ptx	RAFT	P(HPMA-*co*-MAAm)	pH 6 + papain 2 μM	100% after 10 h	217
	Dox	RAFT	P(HPMA-*co*-MAAm)	—	—	218
	Gem + DACH Pt	RAFT	P(HPMA-*co*-MAAm)	—	—	219
	Gem + Ptx	RAFT	P(HPMA-*co*-MAAm)	pH 6 + cathepsin B	Gem: 50% after 30 min	220
					Ptx: 50% after 45 min	
	Gem	RAFT	PHPMA	pH 5.4 + cathepsin B	95% after 3 h	221

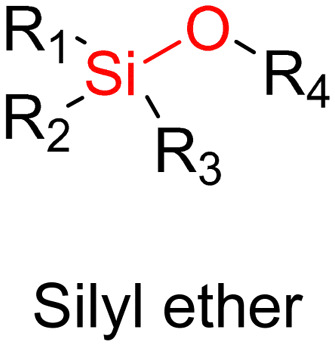	Gem	RAFT	P(HEA-*co*-OEGMA)	pH 5	100% after 20 h	193

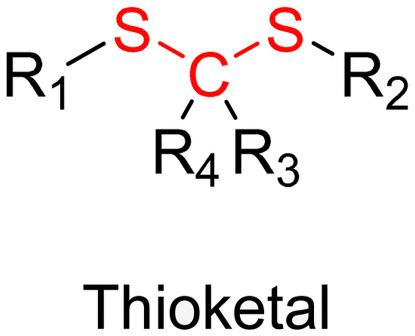	Cpt + Lapa	RAFT	PEG-*b*-P(MA)	100 mM H_2_O_2_ + Fe^2+^	75% after 48 h	207

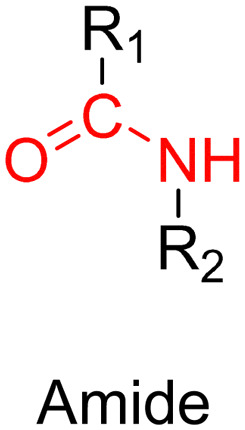	Gem	RAFT	P(SMA)	pH 5 + cathepsin B	70% after 30 days	216

**Table 4:** Dual-sensitive polymer prodrugs based on the combination of endogenous stimuli-sensitive linkers and external stimuli, obtained by RDRP techniques *via* the “grafting through” strategy

External stimuli	Drug	Drug linker	Polymerization method	Nanocarrier	Cleavage conditions	Release	Role of external stimuli	Ref.
Temperature	Dxm	pH-sensitive linkers (A-E)	RAFT	PHPMA	pH 5	<5–90% (A–E) after 500 h	Hydrogel formation at 37 °C	222 and 223

Light	MTIC	Ester + disulfide	RAFT	POEGMA-*b*-PMA3	GSH 10 mM	86% after 12 h	Disulfide cleavage	224
	Dox	Hydrazide	ATRP	PMPC-*b*-PMEMA	pH 5	40% after 72 h	Hyperthermia	226
	Cpt	Ester	RAFT	P(HEA)-*b*-PMPC + PDA NPs	pH 5	91.8% after 12 h	Photo-thermal therapy	227
	Ptx	GFLG peptide	RAFT	P(MAAm)-*b*-POEGMA	Cathepsin B	80% after 8 h	Photo-dynamic therapy	228

### 4.1. The use of endogenous stimuli

#### 4.1.1. pH-Sensitivity

The pH-sensitive polymer prodrugs derived from the “grafting through” strategy were mainly obtained by establishing ester linkages between the drug and the monomer, notably due to the ease of access to monomers bearing hydroxyl and carboxylic groups, while a few other studies reported the incorporation of silyl ether linkages (Fig. 1).

##### 4.1.1.1. Ester linker

Among the different monomers that can be used as precursors to ester linkers, 2-(methacryloyloxy)ethyl monosuccinate (SMA) offers interesting advantages compared to smaller carboxylic acid-bearing vinyl monomers such as MAA. Its copolymerization with vinyl monomers is improved by reducing steric hindrance due to the distance of the drug from the methacrylate moiety, and its central ester bond remains suitable and accessible for acid hydrolysis.

A typical example reported the coupling of SMA with the hydroxyl groups of Cpt and of the kinase inhibitor dasatinib (Dt).^185^ These monomer prodrugs were then copolymerized with OEGMA by the RAFT process to produce the corresponding P(SMA-*ester*-Dt-*co*-OEGMA) and P(SMA-*ester*-Cpt-*co*-OEGMA) hydrophilic polymer prodrugs. P(MAA-*co*-DMAEMA)-*b*-P(SMA-*ester*-Dt) amphiphilic diblock copolymer prodrug nanoparticles of 43 nm in diameter were also prepared by copolymerization of *tert*-butyl methacrylate (*t*BMA) with DMAEMA from a P(SMA-*ester*-Dt) macro-RAFT agent, to evaluate the effect of polymer morphology on drug release kinetics. It was shown that release of Dt was slightly accelerated in pH 5.8 buffer compared to pH 7.4. In addition, while the prodrug nanoparticles gave higher drug loadings compared to the hydrophilic prodrugs (44.0 and 32.5 wt%, respectively), they showed more sustained drug release in human serum (50% Dt released after 384 h and 240 h, respectively). This is due to the localization of the Dt groups in the hydrophobic core of the nanoparticles, which reduces the cleavage of ester functions, compared to when the drug is randomly distributed along a solvated polymer chain. Greater uptake and retention of the prodrug nanoparticles in K562-R1 cells compared to the free Dt was shown, as well as significant accumulation in the tumor after 24 h. Interestingly, copolymer prodrugs containing both drugs prepared by terpolymerization of OEGMA, SMA-*ester*-Cpt and SMA-*ester*-Dt (P(SMA-*ester*-Cpt-*co*-SMA-*ester*-Dt-*co*-OEGMA)), gave greater cytotoxicity *in vitro* on K562-S cells, compared to P(SMA-*ester*-Dt-*co*-OEGMA).

**Fig. 23:**
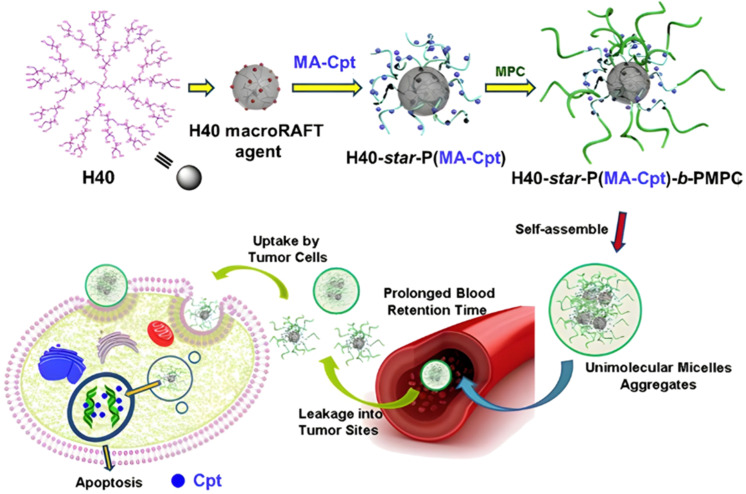
Illustration of the synthesis and delivery of the multi-polymer prodrug-arm hyperbranched H40-*star*-P(MA-Cpt)-*b*-PMPC. Adapted from ref. 187.

Due to the unique features of hyperbranched polymers (*e.g.*, easy synthesis, presence of internal cavities, multiple end-groups, 3D structure),^186^ hyperbranched polymer prodrugs based on Cpt have been developed by RDRP *via* the “grafting through” approach.^187^ Boltorn™ H40 (H40), a fourth generation hyperbranched polyester with hydroxyl terminal groups, was transformed into a H40-*star*-macro-RAFT agent followed by sequential polymerization of Cpt-bearing methacrylate (MA-*ester*-Cpt) and MPC leading to H40-*star*-P(MA-*ester*-Cpt)-*b*-PMPC multi-polymer prodrug-arm hyperbranched nanocarriers of 159 nm in diameter (Fig. 23). An acid-mediated release of Cpt was demonstrated, that reached ∼50% at pH 5 compared to only 10% at pH 7.4 after 36 h. The grafting of Rhodamine B moieties onto the structure allowed to demonstrate their effective internalization by MCF-7 cells after 4 h. *In vivo* administration of the multi-polymer prodrug-arm hyperbranched nanocarriers to tumor-bearing mice gave longer retention time, significant tumor accumulation and greater anticancer efficacy compared to free Cpt.

Using hydrophobic drugs to confer amphiphilicity to a diblock copolymer is a convenient strategy to form polymer prodrug nanoparticles, as previously illustrated with P(MAA-*co*-DMAEMA)-*b*-P(SMA-*ester*-Dt) copolymers.^185^ This approach has also been applied to the hydrophobic drug podophyllotoxin (POD), an antimitotic cyclolignan with antitumor activity, but clinically limited due to its poor solubility and severe side effects.^188^ POD was linked to a methacrylate monomer (MA-*ester*-POD) through ester linkage, followed by RAFT polymerization and chain extension of the obtained P(MA-*ester*-POD) by triethylene glycol methacrylate (TEGMA), to produce amphiphilic diblock copolymer prodrugs P(MA-*ester*-POD)-*b*-PTEGMA with 40 wt% drug loading. They were able to self-assemble into 131 nm-nanoparticles, showing acidic pH-sensitive POD release (52% at pH 5 *vs.* 20% at pH 7.4 after 72 h). Compared to free POD, the nanoparticles also exhibited good blood compatibility, superior cytotoxicity on HeLa cells (5-fold higher compared to free POD) and higher cellular uptake efficacy.

A similar synthetic strategy has also been applied to the design of polymer prodrug nanocarriers physically loaded with a second drug, for combination therapy purposes and to overcome drug resistance. This was exemplified by the design of a monomer prodrug of ibuprofen (HEMA-*ester*-Ibu) by esterification of HEMA by carbodiimide coupling chemistry, followed by its polymerization initiated by PEG-4-formylbenzoic acid (CBA)-Br as an ATRP initiator.^189^ The resulting amphiphilic PEG-*b*-P(HEMA-*ester*-Ibu) diblock copolymers formed micelles of 214 nm with high drug loading (∼47 wt%), and exhibited two levels of pH-sensitivity: (i) the ester bond from HEMA-*ester*-Ibu monomer prodrug and (ii) the benzoic–imine bond at the junction of the two polymer blocks. As a proof of concept, Dox was physically encapsulated into the nanoparticle core, with an encapsulation efficiency of 33%. Cleavage of the benzoic–imine groups at pH 5 led to nanoparticle disassembly and greater Dox release compared to physiological conditions (55 *vs.* 35% after 10 h, respectively). The release of ibuprofen was also accelerated under acidic conditions due to the nanoparticle disassembly that promoted cleavage of ester linkers. Cell assays on B16 cells demonstrated significant ibuprofen activity after ester cleavage and similar *in vivo* anti-tumor activity than free Dox in B16 tumor-bearing mice, but without systemic toxicity.

##### 4.1.1.2. Silyl ether linker

The silyl ether bond is becoming increasingly attractive as linker and crosslinker to produce acid-sensitive nanocarriers (Fig. 1).^190^ It has gained in interest as a protective group because its deprotection rate can be easily tuned by the nature of the substituents (*e.g.*, methyl, ethyl or isopropyl) carried by the silicon atom.^191^ The precise control with time of the linker cleavage could prove useful in achieving sustained drug release. In addition, it is tolerant to radical polymerization conditions, and degradation products after cleavage are non-toxic, allowing the design of acid-sensitive biomaterials with no expected detrimental side effect.^190,192^

**Fig. 24:**
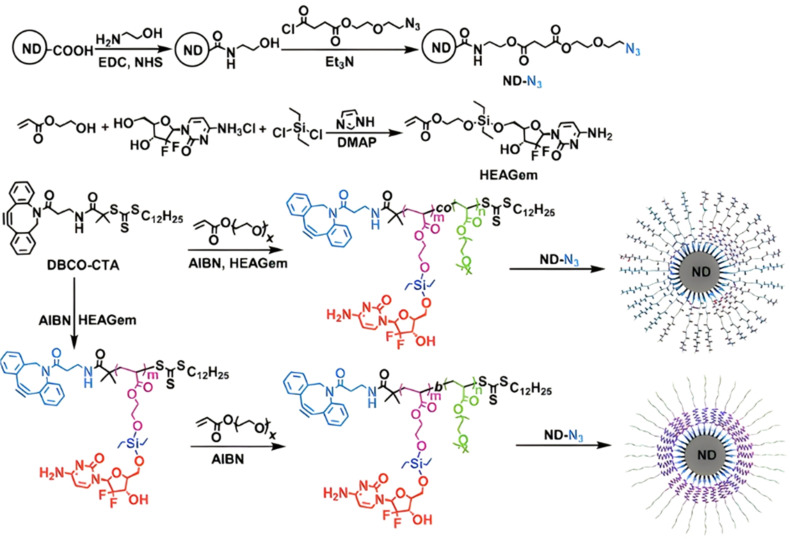
Synthesis routes of Gem-based polymer prodrug with silyl ether linker by the “grafting through” method, and its coating at the surface of NDs. Adapted from ref. 193.

This has been illustrated by the coupling of Gem to HEA *via* a silyl ether bond, obtained by reacting dichlorodiethylsilane in the presence of 4-(dimethylamino)pyridine (DMAP).^193^ HEA-*silyl*-Gem and OEGMA were then copolymerized by RAFT polymerization either simultaneously to yield P(HEA-*silyl*-Gem-*co*-OEGMA) statistical copolymers, or sequentially to produce PHEA-*silyl*-Gem-*b*-POEGMA diblock copolymers. The use of a cyclooctyne-based RAFT agent enabled grafting of the copolymers *via* copper-free click chemistry onto the surface of nanodiamonds (NDs) bearing azide groups (Fig. 24). The resulting coated NDs had an average diameter of between 160 and 270 nm, and gave faster and quantitative Gem release at pH 5 in less than 20 h, compared to pH 7.4 for which the maximum release remained below 80% after almost 100 h. *In vitro* evaluation on AsPC-1 cells showed the greatest cytotoxicity using the smallest nanoparticles with the highest OEGMA content and the longest polymer chains. This increase in cytotoxicity may be due to the shielding effect of the OEGMA coating, as well as better stabilization against aggregation, which improved cellular uptake.

#### 4.1.2. Redox-sensitivity

Polymer prodrugs featuring redox-sensitivity have also been developed by the “grafting through“ method due to the relative ease of inserting redox-sensitive groups between the drug and the monomer, such as disulfide and thioketal linkers.

##### 4.1.2.1. Disulfide bond

**Fig. 25:**
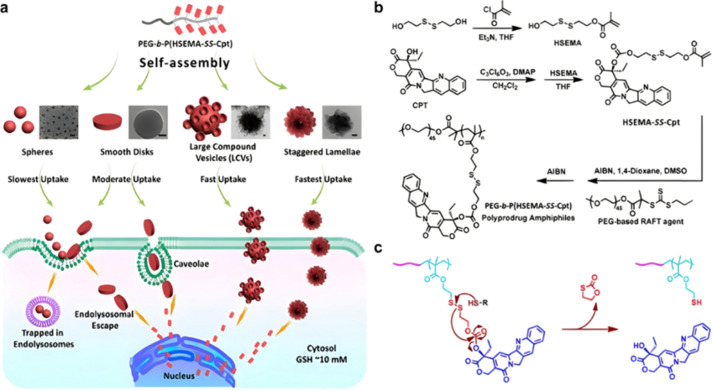
(a) Schematic illustration for the self-assembly of polymer prodrug amphiphiles into different types of nanostructures (*i.e.*, spheres, smooth disks, flowerlike large compound vesicles, and staggered lamellae with spiked periphery), exhibiting a shape-dependent fate during blood circulation, cellular internalization and transport, subcellular distribution, and degradation; (b) synthesis of reduction-responsive Cpt prodrug monomer and PEG-*b*-P(HSEMA-*SS*-Cpt) polymer prodrug amphiphiles; (c) proposed mechanism of reduction-responsive Cpt release from PEG-*b*-P(HSEMA-*SS*-Cpt) polymer prodrug amphiphiles. Adapted from ref. 195.

Disulfide-based prodrugs have proved to be interesting candidates for cancer therapy, thanks to their unique chemical and biophysical properties.^194^ Representative examples obtained by RDRP are those based on Cpt which was conjugated *via* a carbonate bond to monomers containing disulfide bond (–*SS*–). For instance, this was applied to the conjugation of Cpt *via* its 20-hydroxyl group to 2-((2-hydroxyethyl)-disulfanyl)ethyl methacrylate (HSEMA).^195^ The monomer prodrug was then polymerized from a PEG macro-RAFT agent, leading to PEG-*b*-P(HSEMA-*SS*-Cpt) polymer prodrug amphiphiles, exhibiting high drug loadings (>50 wt%) and different morphologies upon self-assembly, such as 180 nm smooth disks, 300 nm staggered lamellae, 790 nm flowerlike large compound vesicles and 43 nm spheres, as a function of the addition rate and composition of the organic solvent during nanoprecipitation (Fig. 25a and b). In terms of biological performances, staggered lamellae and smooth disks gave extended blood circulation times, while staggered lamellae exhibited the fastest cell uptake. Interestingly, staggered lamellae and flowerlike large compound vesicles induced clathrin- and caveolae-independent endocytosis, conversely to smooth disks and spheres. Cpt was efficiently delivered into the cell nucleus by all types of nanostructures, with the exception of spheres, *via* a responsive-reduction release mechanism (Fig. 25c), exhibiting higher cytotoxicity against HepG2 cells.

**Fig. 26:**
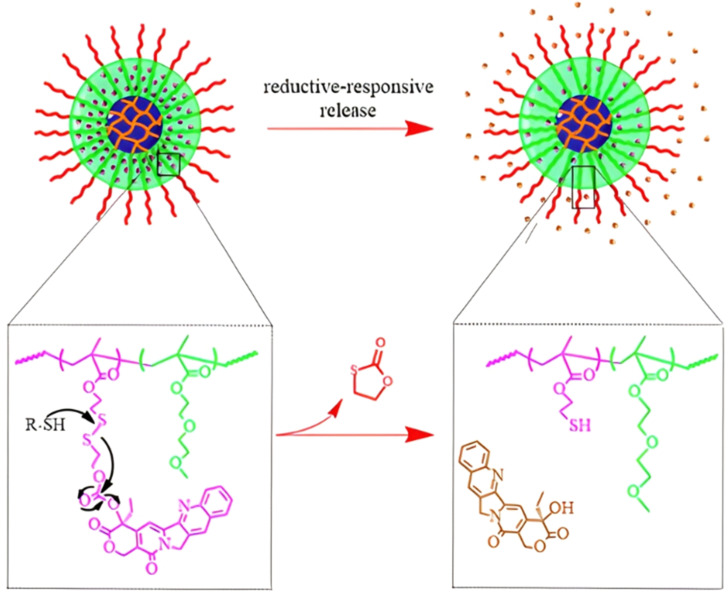
Schematic representation of the polymer prodrug nanogels and the reductive-responsive release mechanism of Cpt. Adapted from ref. 196.

HSEMA-Cpt was similarly engaged in a copolymerization with 2-(2-methoxyethoxy)ethyl methacrylate (EO_2_MA) from a PEG-based macro RAFT agent to give PEG-*b*-P(EO_2_MA-*co*-HSEMA-Cpt) diblock copolymers, exhibiting Cpt contents in the 4.2–12.8 wt% range, depending on the initial monomer ratio (Fig. 26).^196^ Subsequent chain extension by RAFT dispersion polymerization of benzylmethacrylate in presence of *N*,*N*-cystaminebismethacrylamide as a redox-sensitive crosslinker achieved stable 37 nm polymer prodrug nanogels in ethanol/water mixture. Reductive-responsive release of Cpt was demonstrated in GSH solutions (5–10 mM) leading to 35–42% release after 48 h, respectively, conversely to <5% release with 0.01 mM GSH. Two phases of Cpt release were observed: rapid release during the first 5–7 h, followed by slower release thereafter. *In vitro* studies on HeLa cells demonstrated improved internalization compared to free drug and significant cytotoxicity due to the reductive-responsive release of Cpt.

Implementation of imaging capabilities to polymer prodrug nanocarriers obtained by the “grafting through” method has also been investigated for theranostic applications. For instance, incorporation of NIR probes can be carried out by using a NIR probe-bearing methacrylate (MA-NIR) monomer prior the polymerization step. An example of MA-NIR monomer was obtained by conjugating of the hydroxyl groups of aza-BODIPY to 2-(2-bromoacetoxy)ethyl methacrylate *via* nucleophilic substitution.^197^ The MA-NIR monomer (∼1 mol%) was then copolymerized with OEGMA from a POEGMA macro-RAFT agent to form a POEGMA-*b*-P(MA-NIR-*co*-OEGMA) hydrophilic copolymer. It was then chain extended, together with POEGMA-*b*-P(POEGMA-*co*-HSEMA-*SS*-Cpt), by RAFT dispersion polymerization in water of HPMA in presence of ethyleneglycol dimethacrylate (EGDMA) to produce 30 nm-diameter polymer prodrug nanogels with NIR properties. This system proved effective in selectively releasing Cpt at high GSH concentration (10 mM) reaching 70% release after 48 h, conversely to more physiological conditions. Its imaging capability was eventually demonstrated *in vivo* on HeLa tumor-bearing nude mice making this system suitable for fluorescence imaging in real time in addition to anticancer therapeutic activity.

**Fig. 27:**
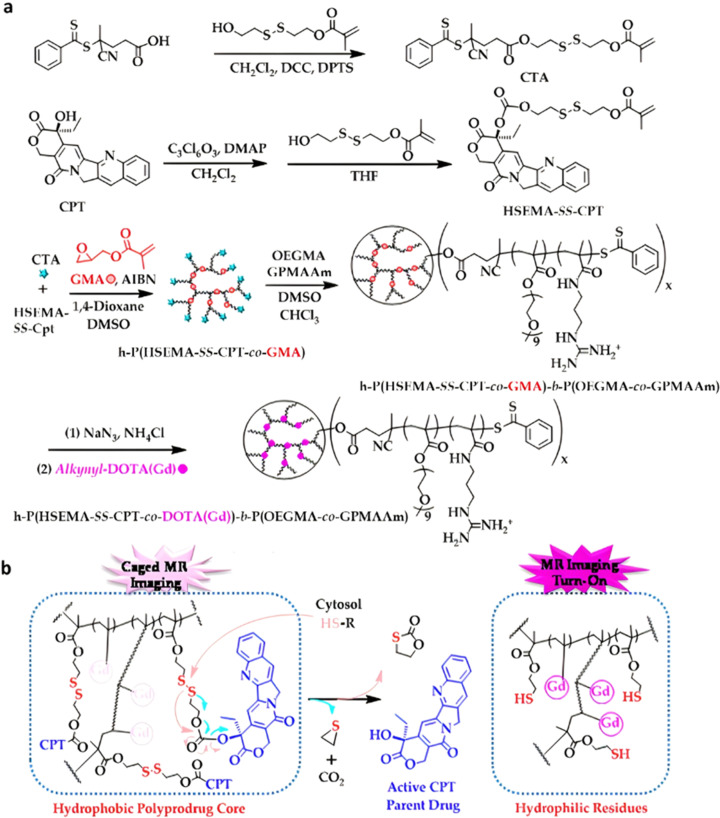
(a) Synthesis of hyperbranched polymer prodrug amphiphiles, h-P(HSEMA-SS-Cpt-*co*-DOTA(Gd))-*b*-P(OEGMA-*co*-GPMAAm); (b) proposed mechanism of reducing environment-activated Cpt release and concomitant hydrophobic–hydrophilic transition of the local milieu surrounding the Gd complex. Adapted from ref. 148.

Magnetic resonance (MR) imaging probes have also been combined to hyperbranched polymer prodrug nanocarriers by the “grafting through” approach.^148^ The synthetic strategy relied on the RAFT copolymerization of HSEMA-*SS*-Cpt and GMA from an inimer-type RAFT agent to give hyperbranched h-P(HSEMA-*SS*-Cpt-*co*-GMA) polymer prodrugs, followed by its chain extension by copolymerization of OEGMA and guanidinopropyl methacrylamide (GPMAAm), resulting in h-P(HSEMA-*SS*-Cpt-*co*-GMA)-*b*-P(OEGMA-*co*-GPMAAm) hyperbranched diblock copolymer prodrugs with 17.3 wt% Cpt (Fig. 27). Post-modification of GMA units *via* azidation then allowed the grafting of the MR contrast agent alkynyl-DOTA (Gd) using “click chemistry”. The resulting sub-100 nm polymer prodrug micelles were characterized by a hydrophobic core containing Cpt and Gd moieties, and by a guanidine-decorated hydrophilic shell. A reducing environment (10 mM dithiothreitol, DTT) resulted in increased Cpt release (60% after 24 h), compared with <5% with a lower concentration (2 μM) of DTT, which also demonstrated a good colloidal and structural stability of the micelles under physiological conditions. During cellular internalization, a reducing environment triggered release of Cpt, resulting in a 70-fold enhancement in cytotoxicity, as well as turn-on of MR imaging with a 9.6-fold increase in *T*_1_ relaxivity. Due to their hydrophilic guanidine-based shell, the polymer prodrug micelles also exhibited extended blood circulation with a half-life up to ∼9.8 h.

**Fig. 28:**
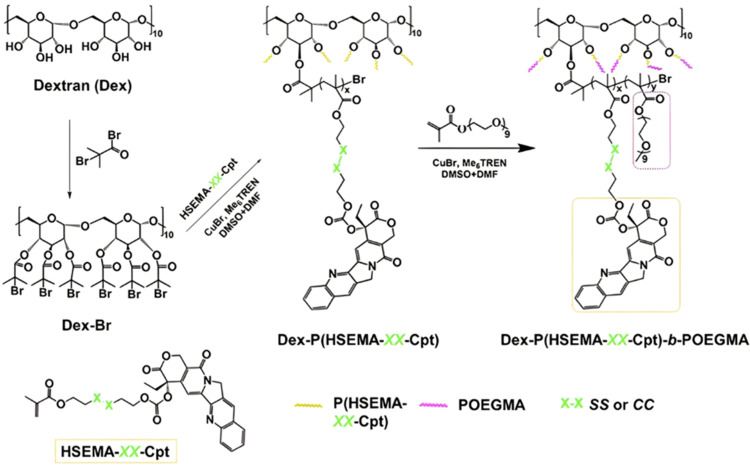
Synthetic route for hyperbranched Dex-P(HSEMA-SS-Cpt)-*b*-POEGMA polymer prodrugs and its carbon–carbon (–CC–) counterpart. Adapted from ref. 198.

Hyperbranched polymer prodrugs can also be obtained from natural polymers, such as dextran (Dex) or CDs. For instance, brominated dextran (Dex-Br) was used as an initiator for the ATRP of HSEMA-Cpt or its analogue with a carbon–carbon bond (–CC–) instead of a disulfide bond (–*SS*–), obtained by coupling Cpt to 5-hydroxy pentyl methacrylate. This was followed by chain extension with OEGMA, resulting in Dex-P(HSEMA-*SS*-Cpt)-*b*-POEGMA or its CC counterpart, respectively, capable of self-assembly into 54–74 nm micelles (Fig. 28).^198^ In a reducing environment (10 mM DTT), only prodrugs containing the disulfide bond demonstrated quantitative Cpt release (100% *vs.* <20% for –CC– counterpart, after 72 h). This was confirmed by *in vitro* studies on HeLa and MCF-7 cells, during which no cytotoxicity was shown from the prodrugs with the –CC– bond, with >90% cell viabilities, while similar cytotoxicity as free Cpt was obtained from the SS-containing prodrug.

**Fig. 29:**
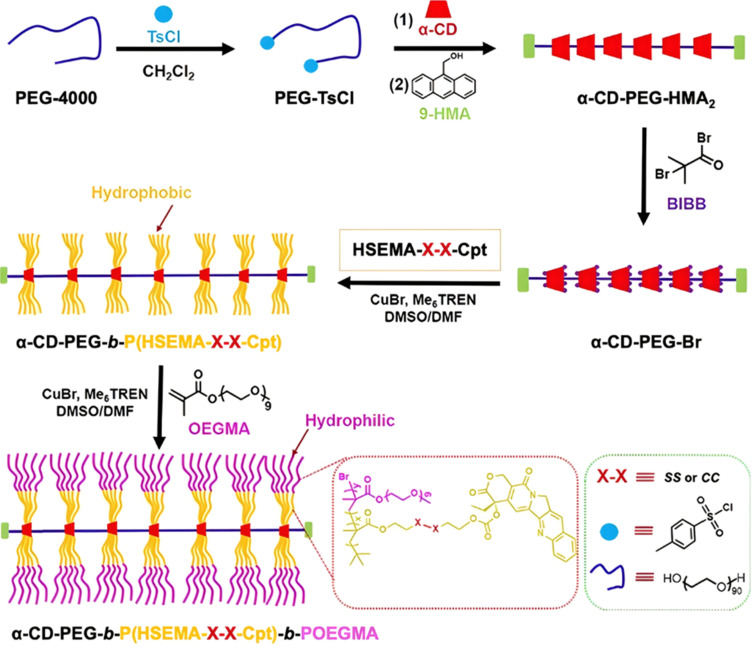
Synthesis route for unimolecular polymer prodrug micelles based on α-CD-PEG-*b*-P(HSEMA-*SS*-Cpt)-*b*-POEGMA and α-CD-PEG-*b*-P(HSEMA-CC-Cpt)-*b*-POEGMA. Adapted from ref. 200.

CDs are an interesting class of building blocks for the design of polymer prodrugs due to their biodegradability and the presence of 21 hydroxyl groups available for functionalization.^199^ Similarly to the above-mentioned Dex-based polymer prodrugs, HSEMA-*SS*-Cpt or its counterpart without the disulfide bond (–CC–) were copolymerized with OEGMA from a α-CD-PEG-Br polyrotaxane as an ATRP macroinitiator, leading to multi-arm α-CD-PEG-*b*-P(HSEMA-*SS*-Cpt)-*b*-POEGMA prodrugs and its –CC– counterpart (Fig. 29).^200^ Both supramolecular structures self-assembled in water to give monodisperse spherical micelles of respectively 114 and 120 nm. Drug release and *in vitro* studies were in line with the previous study based on Dex-based prodrugs, showing enhanced release of Cpt when exposed to DTT and higher rate of apoptosis compared to the free drug. In addition, the micellar prodrugs were rapidly and significantly internalized by HeLa cells (>90% after 2 h).

**Fig. 30:**
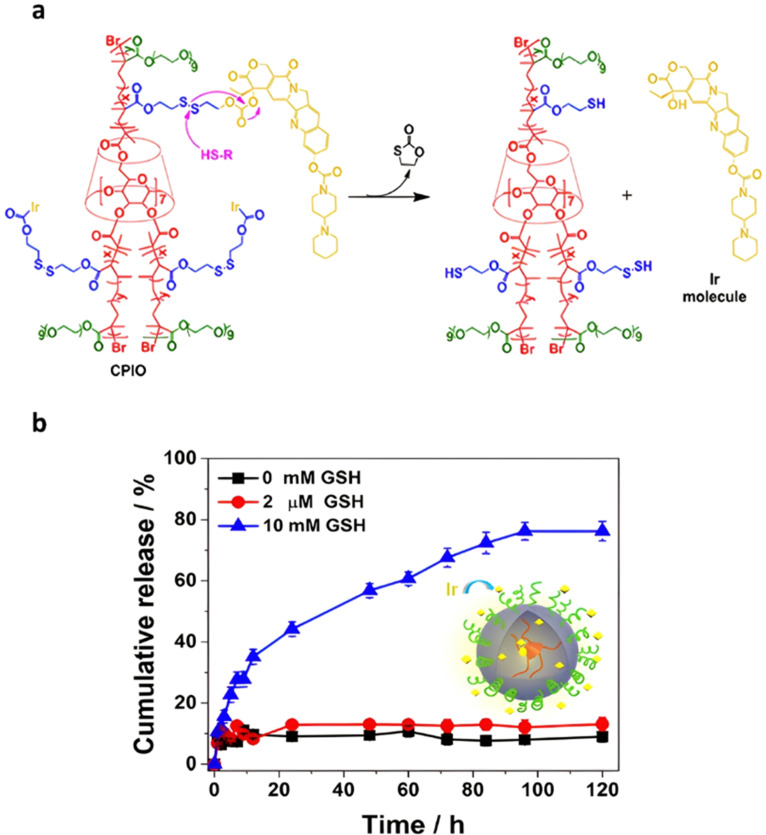
(a) Reductive-driven degradation of α-CD-P(HSEMA-*SS*-Ir-*co*-OEGMA) polymer prodrug nanoparticles; (b) associated Ir release profiles at 37 °C as function of different concentrations of reductive GSH (0, 2 μM and 10 mM). Adapted from ref. 201.

Similarly to Cpt, HSEMA has also been conjugated to the anticancer agent irinotecan (Ir) *via* establishment of a carbonate bond, to produce reducible star-like α-CD-P(HSEMA-*SS*-Ir-*co*-OEGMA) polymer prodrugs by copolymerization of HSEMA-*SS*-Ir and OEGMA from α-CD-Br ATRP (Fig. 30a).^201^ They were able to self-assemble into 50 nm micelles of ∼31 wt% drug loading, that remained stable in non-reductive conditions, whereas at high concentrations in GSH (10 mM), release of Ir reached 80% after 120 h *vs.* <15% for 0 and 2 μM GSH (Fig. 30b). Cytotoxicity assays on HeLa cells showed improved cytotoxicity of the polymer prodrugs compared to free Cpt (45% *vs.* 25% of cell viability, respectively). Similar results were obtained on MCF-7 cells, and hematology assays have demonstrated sufficient biocompatibility for other *in vivo* applications.

Another example of reducible monomer-Cpt was achieved by the coupling of Cpt to 3-((3-(2-(methacryloyloxy)ethoxy)-3-oxopropyl)disulfanyl)propanoic acid (HEMA-*SS*-COOH), a disulfide-containing monomer.^202^ The obtained HEMA-*SS*-Cpt was copolymerized with OEGMA from a carboxylic acid-bearing ATRP initiator, aiming to increase the water solubility of the polymer prodrug while protecting Cpt from early degradation. The LHRH (Luteinizing Hormone-Releasing Hormone) targeting peptide was eventually grafted to the carboxylic acid chain end of HOOC-P(HEMA-*SS*-Cpt)-*b*-POEGMA polymer prodrugs to facilitate its cellular uptake by cancer cells that overexpressed LHRH receptors.^203^ The resulting 20 nm-micelles induced significant Cpt release, reaching 90% after 24 h under 5 mM of DTT. The targeting properties of the polymer prodrug were evaluated on overexpressing LHRH receptor cell lines (A2780, IGROV-1, CACO-2), showing comparable cytotoxicity to that of free Cpt, conversely to the low cytotoxicity obtained on a LHRHR-negative cell line (CALU-3).

##### 4.1.2.2. Thioketal bond

**Fig. 31:**
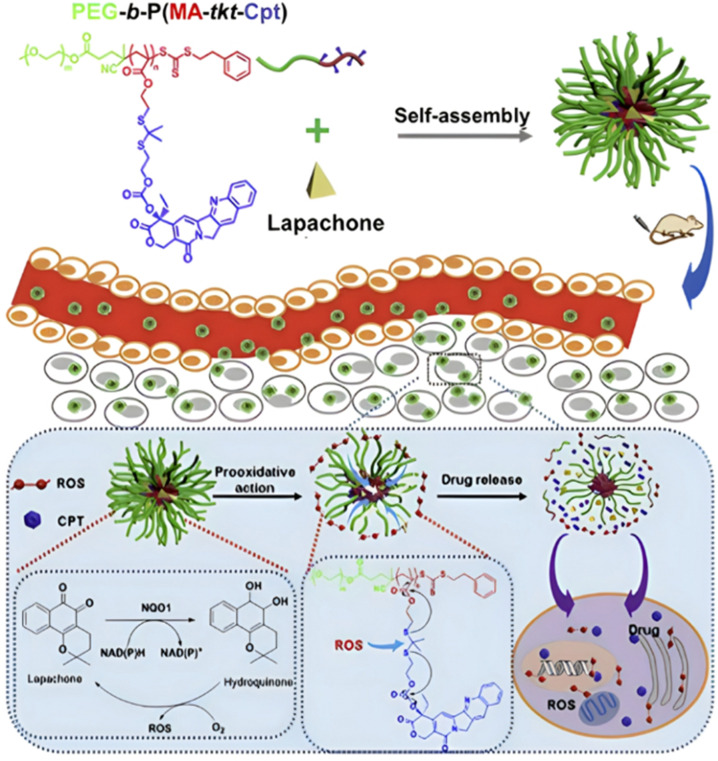
Synthetic scheme for Lapa-loaded, PEG-*b*-P(MA-tkl-Cpt) polymer prodrugs micelles and their tumor-specific oxidative stress amplification for ROS-driven Cpt release. Adapted from ref. 207.

Redox-responsive polymer prodrugs derived from the “grafting through” method can also target the tumoral environment by having cleavable chemical bonds between the drug and the monomer that are sensitive to reactive oxygen species (ROS),^204^ which are often observed in cancer cells and play a key role in angiogenesis.^205^ The thioketal (tkl) bond is a representative example in this area (Fig. 1) as it is degraded through a self-immolative pathway when exposed to high levels of ROS.^206^ Similarly to disulfide-containing Cpt-monomers, Cpt was conjugated to 2-((2-((2-hydroxyethyl)thio)propan-2-yl)thio)ethyl methacrylate which contains a thioketal group between the methacrylate moiety and the drug (Fig. 31).^207^ Such monomer (MA-tkl-Cpt) was polymerized by RAFT from a PEG-based macro-RAFT agent, to give redox-responsive polymer prodrug micelles of 48 nm in diameter. They were also able to physically encapsulate a second hydrophobic drug, β-lapachone (Lapa), known to specifically increase the ROS level in cancer cells,^208^ with high encapsulation efficiency (98.5%) and a loading in Lapa of 9%. Such dual-drug micelles aimed to induce a ROS production *via* Lapa release followed by the ROS-mediated thioketal cleavage (Fig. 31), triggering the Cpt release. In absence of ROS, the Cpt release remained negligible, avoiding a toxic release in healthy tissues, whereas high levels of ROS (1 mM H_2_O_2_ + Fe^2+^) led to enhanced release of Cpt. Surprisingly, an early burst release of Lapa was observed at very low ROS levels, probably due to the non-covalent interactions between Lapa and the polymer chains. *In vitro* studies have demonstrated a significant ROS production induced by Lapa in 4T1 cancer cells as well as a synergistic activity with higher cytotoxicity of dual-loaded micelles compared to the physical mixture of the two drugs. *In vivo* administration of dual-loaded micelles in 4T1 tumor-bearing mice confirmed their specific accumulation at the tumor site, resulting in enhanced antitumor efficacy and in suppression of tumor growth, without systemic toxicity.

##### 4.1.2.3. Redox- and pH-sensitivities

**Fig. 32:**
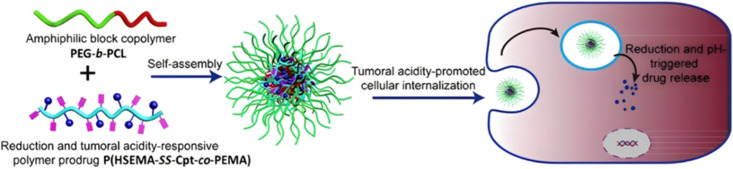
Schematic illustration of the self-assembly and acid-driven cell internalization of PEG-*b*-PCL micelles loaded with P(HSEMA-*SS*-Cpt-*co*-PEMA) polymer prodrugs. Adapted from ref. 209.

The combination of redox-sensitivity through insertion of disulfide or thioketal bonds, and pH-sensitivity *via* the use of pH-sensitive polymer blocks, has been investigated to improve the drug delivery to tumors. Such dual-stimuli responsive polymer prodrugs deriving from the “grafting through” approach have been obtained by RAFT copolymerization of HSEMA-*SS*-Cpt and 2-(piperidin-1-yl)ethyl methacrylate (PEMA) as a pH-responsive monomer, leading to P(HSEMA-*SS*-Cpt-*co*-PEMA) random copolymers with high drug loadings (13.5–27.1%) (Fig. 32).^209^ Their co-nanoprecipitation with PEG-*b*-PCL resulted in sub-50 nm micelles exhibiting <5% release of Cpt at pH 7.4 under non-reductive environment after 48 h, 20% release within 50 h under reductive environment (5 mM GSH) at pH 7.4, and 50–80% release after 50 h when combining reductive environment with acidic pH (6.8). Decreasing the pH to 5.4 only slightly increased the release of Cpt, probably due to the protonation of PEMA units in the 6.8–7.4 pH range, leading to the loose structure of cores. *In vitro* and *in vivo* evaluations demonstrated enhanced cell internalization in HepG2 cells and greater cytotoxicity, together with effective growth suppression of multicellular tumor spheroids at pH 6.8, respectively.

**Fig. 33:**
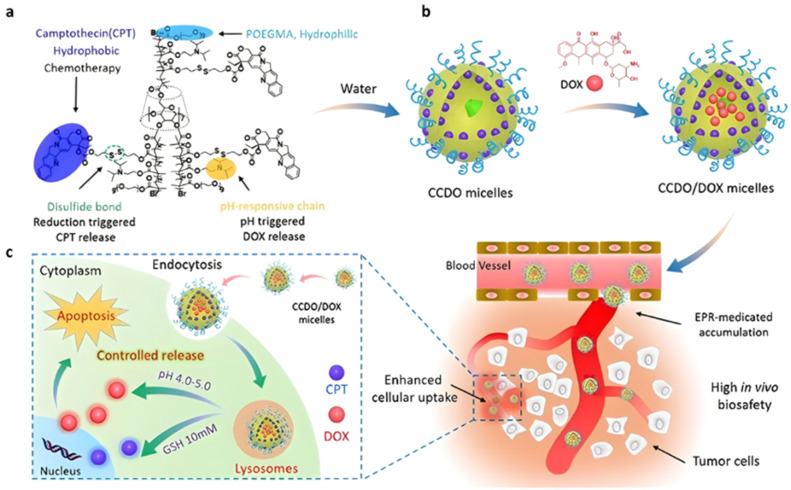
(a) Chemical structure of dual-sensitive (*i.e.*, redox and pH) Cpt-based α-CD-P(HSEMA-*SS*-Cpt-*co*-OEGMA-*co*-DPA) polymer prodrug; (b) self-assembly in water and physical encapsulation of Dox; (c) cellular release mechanism induced by pH and reductive environment. Adapted from ref. 210.

Combining redox and pH sensitivities can also enable separate delivery of two different drugs, such as Cpt and Dox. Terpolymerization of HSEMA-*SS*-Cpt, OEGMA and 2-(diisopropylamino)ethyl methacrylate (DPA) from a trifunctional α-CD ATRP initiator led to α-CD-P(HSEMA-*SS*-Cpt-*co*-OEGMA-*co*-DPA) 3-arm star polymer prodrugs (Fig. 33a).^210^ Interestingly, they could be formulated into 69 nm stable micelles loaded with Dox (Fig. 33b), with a loading in Cpt and Dox of 18.9 wt% and 5.2 wt%, respectively. Reductive environment (10 mM GSH) led to significant release of Cpt (75% after 48 h), while slightly acidic conditions (pH 5) promoted release of Dox (70% after 72 h), due to protonation of DPA units, creating a more hydrophilic environment (Fig. 33c). Cell viability assays on HeLa and MCF-7 cells demonstrated synergistic effect of the drug combination compared to Dox-free micelles, as well as good blood compatibility.

#### 4.1.3. Enzymatic-sensitivity

As with the “grafting to” strategy, enzymatically cleavable bonds can be inserted between the drug and the monomer using the “grafting through” approach, targeting the two main enzyme families, esterases^130,154,155,168^ and proteases,^150,153,173^ for the cleavage of ester and peptidyl/amide bonds, respectively.

##### 4.1.3.1. Esterases: ester bond hydrolysis

**Fig. 34:**
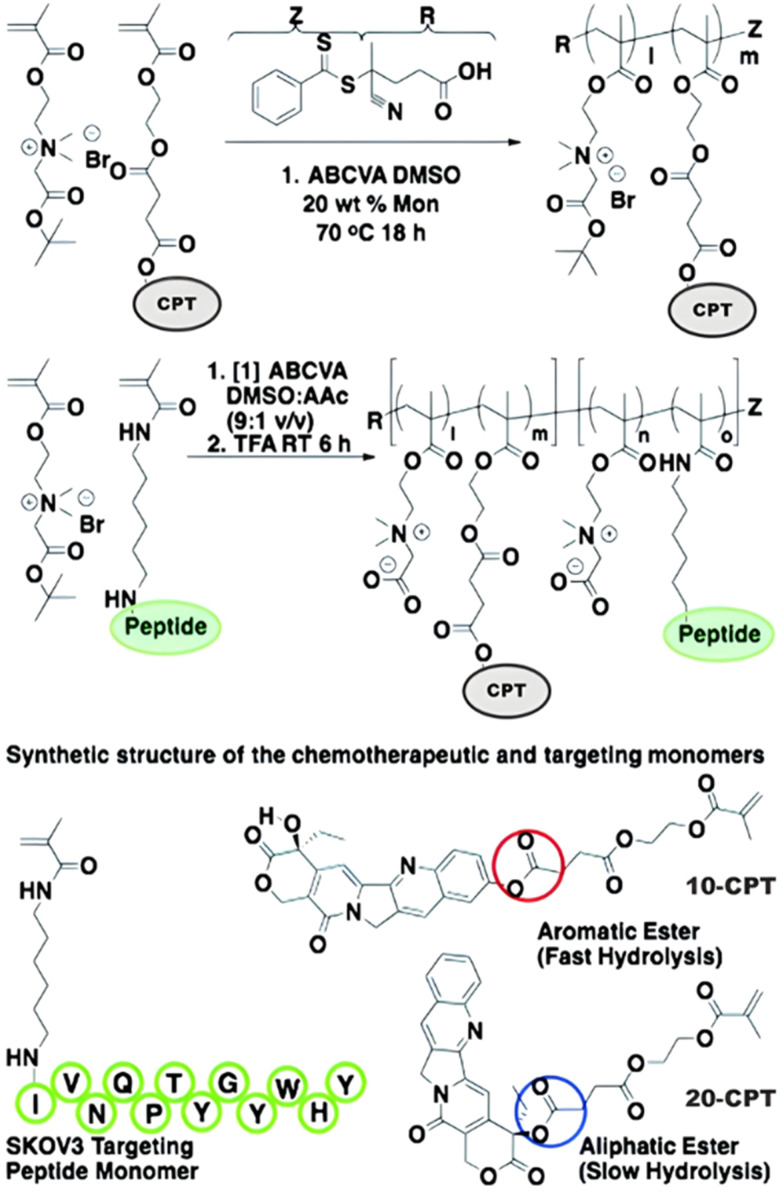
Synthetic scheme for the preparation of GE11 peptide-functionalized diblock copolymer prodrugs *via* coupling to Cpt using an aliphatic ester bond (20 Cpt) or an aromatic ester bond (10 Cpt). Adapted from ref. 211.

The central ester group of the SMA monomer, previously used to develop pH-sensitive polymer prodrugs nanocarriers, was also found to be effective in conferring enzymatic sensitivity. For example, SMA-*ester*-Cpt was copolymerized with carboxy betaine methacrylate (CBM) by RAFT polymerization to yield hydrophilic, zwitterionic polymer prodrugs (Fig. 34).^211^ To modulate the drug release kinetics, coupling between SMA and Cpt was performed *via* carbodiimide chemistry, either on the hydroxyl groups of the aliphatic ester of Cpt (SMA-*ester*-20Cpt), or on the aromatic ester of Cpt (SMA-*ester*-10Cpt) (Fig. 34). P(CBM-*co*-SMA-*ester*-10Cpt) and P(CBM-*co*-SMA-*ester*-20Cpt) gave drug loadings of ∼20 wt%. Due to the different nature of the linkers, and probably to their accessibility by enzymes, P(CBM-*co*-SMA-*ester*-10Cpt) showed faster Cpt release than P(CBM-*co*-SMA-*ester*-20Cpt) in human serum (37 and 12% after 4 days, respectively). Such difference in drug release has been directly translated into cytotoxicity on SKOV3 cells, with an IC_50_ value ∼100 times higher for P(CBM-*co*-SMA-*ester*-10Cpt) compared to P(CBM-*co*-SMA-*ester*-20Cpt). To target the epidermal growth factor receptors overexpressed in cancer cells, these two polymer prodrugs where chain extended by copolymerization of CBM and GE11 peptide-functionalized methacrylamide (GE11-MAAm) (Fig. 34). Flow cytometry studies showed a 2-fold increase in binding for the targeted polymer prodrug relative to the untargeted counterparts.

The synthesis of enzyme-sensitive polymer prodrug nanocarriers based on the anticancer drug methotrexate (Mtx) has also been attempted by the “grafting through” approach. It was carried out by esterification of HPMA with Mtx (HPMA-*ester*-Mtx) under DCC/DMAP coupling conditions, followed by its RAFT polymerization from a PHPMA macro-RAFT agent in presence of EGDMA, resulting in core-crosslinked star polymer prodrugs of 20 nm with a Mtx loading of 20 wt%.^212^ Whereas porcine liver esterase achieved a significant release of Mtx from HPMA-Mtx (30% after 96 h), no release was observed from the core-crosslinked star polymer prodrugs, probably due to poor enzymatic access to the ester bond. This study highlighted the key role of polymer architecture and linker environment in its cleavage and drug release efficiency.

**Fig. 35:**
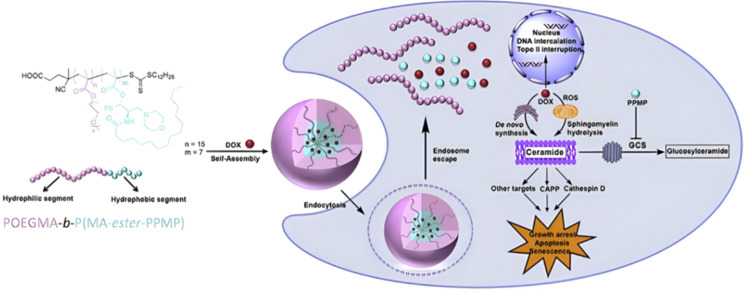
Structure of POEGMA-*b*-P(MA-*ester*-PPMP) amphiphilic diblock copolymer prodrug, physical encapsulation of Dox during its self-assembly, as well as proposed cellular mechanism of action after endosomal escape and drug release. Adapted from ref. 215.

1-Phenyl-2-palmitoylamino-3-morpholino-1-propanol) (PPMP) is a potent inhibitor of glucosylceramide synthase (GCS), which can induce cell death and synergies with chemotherapeutic agents by increasing the level of ceramides in tumor cells and by overcoming cell resistance.^213,214^ It has been turned into a methacrylate monomer through ester bonding (MA-*ester*-PPMP) and polymerized from a POEGMA macro-RAFT agent to give POEGMA-*b*-P(MA-*ester*-PPMP) amphiphilic diblock copolymer prodrugs (Fig. 35).^215^ Dox was encapsulated during self-assembly of the copolymer to give 105 nm micelles with a Dox loading of 6.5 wt%. Even if Dox release was only evidenced in PBS (10% after 2 h), *in vitro* studies in 4T1.2 cells demonstrated synergistic activity of dual-loaded micelles likely due to the release of PPMP *via* cleavage of ester bonds by tumor esterases.

##### 4.1.3.2. Proteases: amide and GFLG peptide bonds hydrolysis

Only a few examples of amide bond-containing prodrug monomers have been reported as these bonds remain very stable in physiological conditions. The amine group of Gem was subjected to amidation with the carboxylic acid group of SMA, leading to the SMA-*amide*-Gem prodrug monomer comprising two cleavable linkers: (i) a central ester bond and (ii) an amide bond connected to Gem.^216^ SMA-*amide*-Gem was polymerized by the RAFT technique resulting in high Gem loading (50 wt%) P(SMA-*amide*-Gem) polymer prodrugs, able to self-assemble into 90 nm nanoparticles. The Gem release was greater under acidic conditions (pH 5) compared to physiological conditions (20% *vs.* 50%, respectively, after 30 days), and further enhanced in presence of cathepsin B at pH 5, leading to 70% Gem release after 30 days. Whereas P(SMA-*amide*-Gem) polymer prodrug nanoparticles conducted to lower cytotoxicity on Mia PaCa-2 cells compared to the free drug at low doses after 72 h, they exhibited superior cytotoxicity over a prolonged time frame (30 days).

**Fig. 36:**
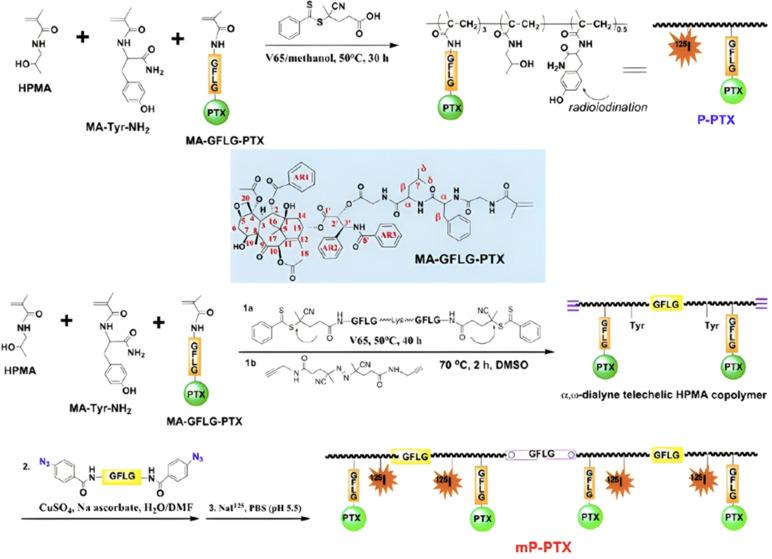
Synthetic strategy for P(HPMA-*co*-MAAm-GFLG-Ptx) copolymer prodrugs (P-PTX) and their multiblock counterparts obtained (mP-PTX) by Cu(i)-catalyzed click chemistry. Adapted from ref. 217.

Due to its versatility, the GFLG peptide was also advantageously used as a protease-responsive linker for the design of polymer prodrugs from the “grating through” method. An efficient strategy relied on the successful functionalization of GFLG-bearing methacrylamide by a small library of anticancer drugs, such as paclitaxel (Ptx),^217^ Dox,^218^ and Gem.^219,220^ These prodrug monomers were copolymerized with HPMA by the RAFT process to produce two different families of polymer prodrugs: (i) P(HPMA-*co*-MAAm-GFLG-drug) copolymer prodrugs and (ii) multiblock copolymer prodrugs in which the different P(HPMA-*co*-MAAm-GFLG-drug) blocks were connected through GFLG linkers *via* Cu(i)-catalyzed or thiol–ene click chemistry (Fig. 36). For instance, with the Ptx-based polymer prodrugs,^217^ it was shown that the cysteine protease papain caused the molecular weight of multiblock copolymer prodrugs to decrease to half the original value due to cleavage of GFLG moieties. Cumulative releases of Ptx over time were similar for both types of copolymer prodrug, even if a slight decrease in Ptx release from the multiblock copolymer prodrugs was observed, probably linked to the formation of a more compact coil due to enhanced hydrophobic interactions. After radiolabeling of the prodrugs (Fig. 36), *in vivo* studies highlighted the long-circulating properties of the multiblock copolymer prodrugs, with an increased half-life of 27.5 h compared to 13 h for the simple copolymer prodrugs, and only 2 h for Ptx. The multiblock copolymer prodrugs also exhibited greater anticancer efficacy in A2780 human ovarian tumor-bearing mice. Similar results were obtained for the Dox-based multiblock copolymer prodrugs as they were the most efficient for tumor growth inhibition, with an optimal molecular weight of about 100 kDa to enhance the antitumor efficacy.^218^ This versatility of the PHPMA drug delivery platform has also been applied to the co-delivery of Gem and diaminocylohexane platinum (DACH Pt) and of Gem and Ptx by simple copolymerization of the respective monomer prodrugs (or the chelating ligand-bearing monomer in case of DACH Pt).^219,220^

**Fig. 37:**
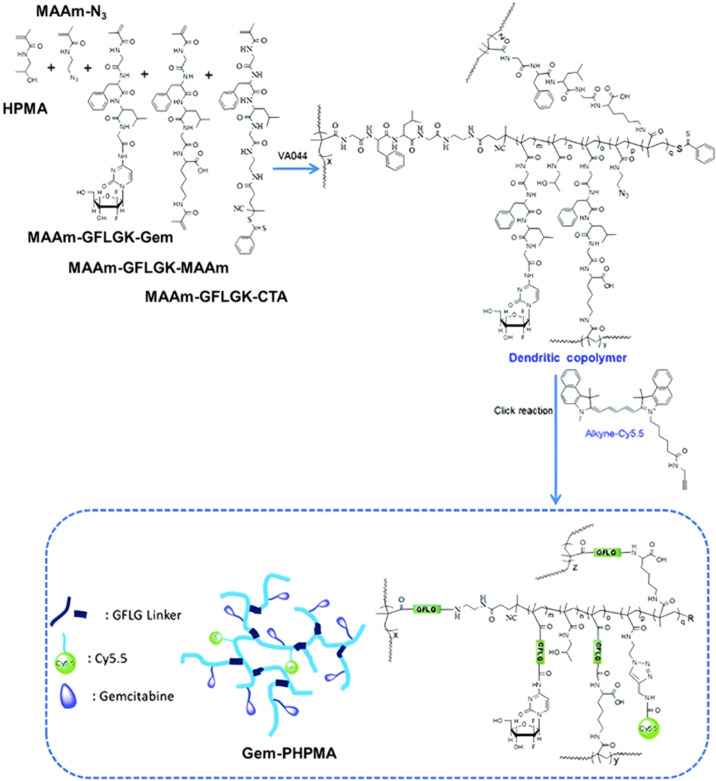
Synthesis route for hyperbranched, crosslinked Gem-PHPMA polymer prodrugs. Adapted from ref. 221.

Similarly, hyperbranched, crosslinked Gem-PHPMA polymer prodrugs (Gem loading of 5.6 wt%) have been obtained *via* RAFT copolymerization of MAAm-GFLGK-Gem, HPMA, MAAm-GFLGK-MAAm, MAAm-GFLG-4-cyanopentanoic acid dithiobenzoate as the CTA and MAAm-N_3_ for further click chemistry with a NIR dye alkyne (Fig. 37).^221^ 55 to 85 nm stable polymer prodrug nanoparticles were obtained upon self-assembly, with >95% of Gem released after 3 h in presence of cathepsin B at pH 5.4, whereas no release was observed at pH 5.4 without cathepsin B. The polymer prodrug nanoparticles exhibited long-circulating properties and slow clearance through kidney filtration, as well as greater tumor inhibition compared to the free Gem.

### 4.2. The combination of endogenous and exogenous stimuli

Polymer prodrugs synthesized by RDRP techniques *via* the “grafting through” method can also be engineered to be sensitive to both endogenous (*e.g.*, pH, reductive environment, enzymatic action) and to exogenous stimuli (*e.g.*, temperature, light suitable) (Table 4).

#### 4.2.1. Linkers sensitive to endogenous stimuli combined to temperature-sensitivity

**Fig. 38:**
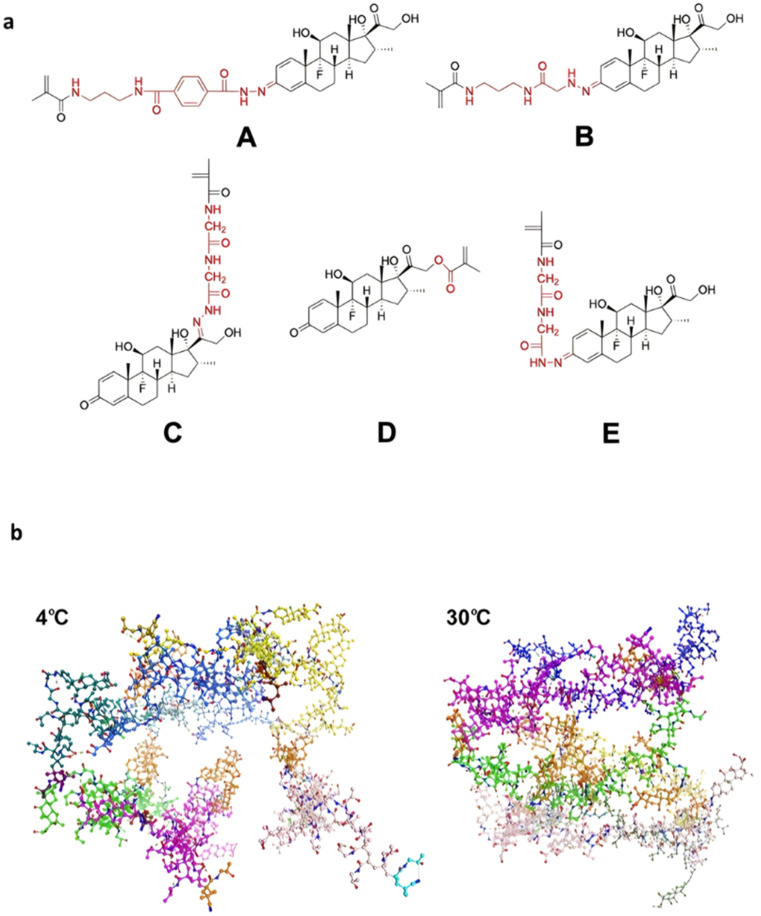
(a) Chemical structures of the six different Dxm-based prodrug monomers A–E; (b) molecular representations from dynamic simulations of Dxm-based hydrogel in water at 4 °C and at 30 °C. Six ProGel-Dxm polymers are colored in yellow, blue, dark green, bright green, magenta, and pale colors. The Dxm molecules are colored in orange. Adapted from ref. 223.

To develop macromolecular prodrugs for the treatment of rheumatoid arthritis (RA) with fine-tuned activation kinetics, prodrug monomers of dexamethasone (Dxm) have been equipped with five different pH-sensitive linkers (Fig. 38a, monomers A to E), followed by their RAFT copolymerization at 10 wt% with HPMA to give a small library of P(HPMA-*co*-M-Dxm) (with M = A to E) water-soluble polymer prodrugs.^222^ Under acidic conditions (pH 5 and 6), P(HPMA-*co*-E-Dxm) gave the fastest Dxm release and P(HPMA-*co*-D-Dxm) the slowest, while they were all relatively stable (<10% release after 500 h) under physiological conditions (pH 7.4). In human serum, P(HPMA-*co*-B-Dxm) and P(HPMA-*co*-D-Dxm) achieved 5% release of Dxm, while the other polymer prodrugs achieved <1% release. Interestingly, only P(HPMA-*co*-B-Dxm) enabled 50% release of Dxm in rat serum, while the other structures achieved a maximum of 6% release. These results showed a broad spectrum of activation kinetics. The *in vivo* evaluation demonstrated that the P(HPMA-*co*-E-*hyd*-Dxm) polymer prodrug, which contains a hydrazone linker, was the most effective in preserving joint structural integrity in a rat model of adjuvant-induced arthritis.

It is interesting to note that increasing the Dxm content from 10 to 24 wt% led to the appearance of a phase transition temperature and the formation of a hydrogel around 30 °C.^223^ Molecular dynamics simulations revealed a correlation between Dxm content and drug release kinetics from the hydrogel, as the higher the Dxm content, the slower the release, which was probably due to two factors: (i) an increase in hydrophobic aggregation for higher drug amounts and (ii) a more limited exposure (on the surface of the hydrogel only) to the releasing medium. This computational study also highlighted an interconnected conformation in which hydrophobic Dxm molecules could coalesce at 30 °C, while a more extended conformation was obtained at 4 °C (Fig. 38b). Such dual-sensitive polymer prodrug hydrogel was able to be retained in the synovial cavity for at least one month, enabling a sustained drug release by slow dissolution of the polymer scaffold and release of water-soluble polymer prodrugs before being processed by phagocytic synoviocytes. It has been shown to sustainably improve joint inflammation and pain in a rodent model of inflammatory arthritis and osteoarthritis.

#### 4.2.2. Linkers sensitive to endogenous stimuli combined to light-sensitivity

**Fig. 39:**
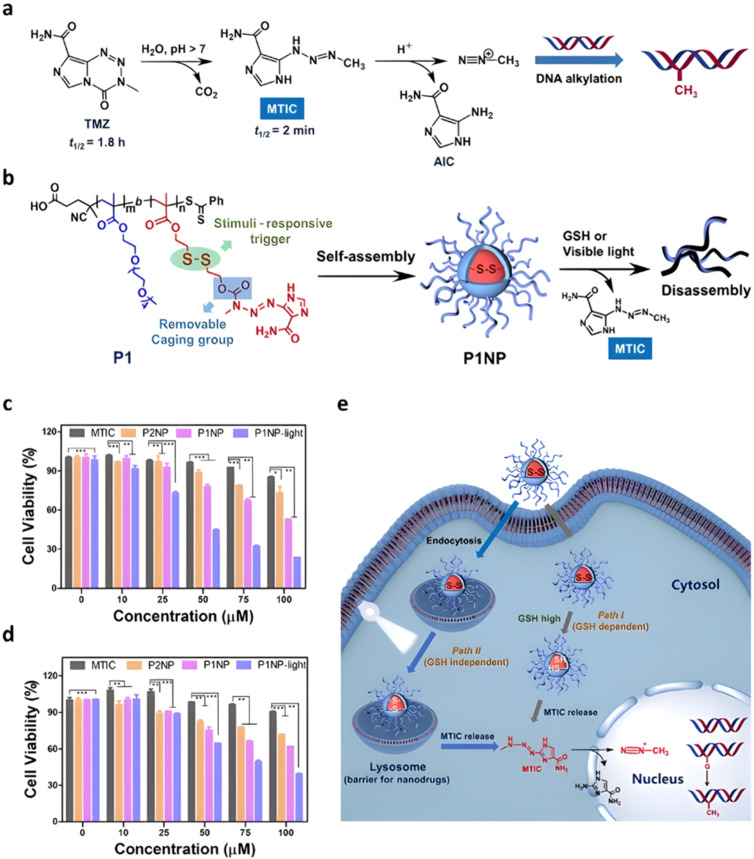
(a) Hydrolysis mechanism of the TMZ anticancer agent; (b) structure, self-assembly and drug release of reductive-responsive MTIC-based polymer prodrug; cell viabilities of: (c) U87MG cells and (d) T98G cells after 72 h of treatment with free MTIC, non-redox-responsive polymer prodrug nanoparticles (P2NP) and redox-responsive polymer prodrug nanoparticles (P1NP) under light irradiation (LED 405 nm, 40 mW cm^−2^) or not. *n* = 3, *p < 0.05, **p < 0.01, and ***p < 0.001; (e) schematic representation of the proposed intracellular effects of redox-responsive polymer prodrug nanoparticles. Adapted from ref. 224.

Light sensitivity has also been implemented to polymer prodrugs from the “grafting through” method already sensitive to endogenous stimuli, to improve drug release efficiency. For example, the active intermediate (3-methyltriazene-1-yl)imidazole-4-carboxamide (MTIC) of the anticancer agent temozolomide (Tmz) has been conjugated to a disulfide-bearing and light-sensitive monomer 2-((2-(((4-nitrophenoxy)carbonyl)oxy)ethyl)disulfanyl)ethyl methacrylate,^224^ in order to increase its short half-life (∼2 min) *via* the design polymer prodrugs (Fig. 39a).^225^ The redox- and light-responsive prodrug monomer was polymerized from a POEGMA macro-RAFT agent to achieve diblock copolymer prodrugs able to form nanoparticles of 135 nm in diameter upon self-assembly (Fig. 39b). It was shown that prodrug activation successfully occurred *in vitro* following two different pathways: (i) high GSH levels (GSH dependent pathway) triggered the release of MTIC by thiol/disulfide exchange followed by tandem reactions, inducing decaging of MTIC, or (ii) under low GSH levels (GSH independent pathway), visible light (LED 405 nm, 40 mW cm^−2^) induced release of MTIC *via* homolytic disulfide scission, followed by tandem reactions and MTIC decaging (Fig. 39b). Cell viability assays on U87MG and T98G cells showed that light activation applied on the redox-responsive polymer prodrug nanoparticles gave the highest cytotoxicity, compared to free MTIC (which is rapidly degraded), non-redox-responsive polymer prodrug nanoparticles and redox-responsive polymer prodrug nanoparticles without light (Fig. 39c–e).

**Fig. 40:**
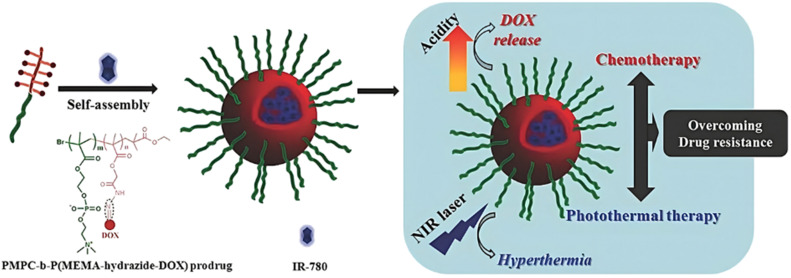
Schematic representation of IR-780-loaded PMPC-*b*-P(MEMA-*hyd*-Dox) diblock copolymer prodrug nanoparticles for chemo-photothermal therapy to overcome drug resistance. Adapted from ref. 226.

Light irradiation can also be combined to endogenous stimuli for anticancer therapeutic strategies based on hyperthermia, and photothermal and photodynamic therapies (PTT and PDT, respectively), using NIR irradiation. For example, a pH-sensitive prodrug monomer of Dox was synthesized by coupling Dox to MEMA *via* a hydrazide linker, followed by its copolymerization with MPC by ATRP to achieve PMPC-*b*-P(MEMA-*hyd*-Dox) diblock copolymer prodrugs with 10.7 wt% Dox (Fig. 40). Their self-assembly into 170 nm nanoparticles also enabled the encapsulation of the IR-780 photosensitizer with a loading efficiency of 4.8%.^226^ Upon NIR laser irradiation and acidic condition-mediated Dox release, the polymer prodrug nanoparticles significantly enhanced intracellular Dox accumulation and induced the cell apoptosis in Dox-resistant MCF-7/ADR cells. In addition, hyperthermia induced significant inhibition of MCF-7/ADR tumor growth in tumor-bearing mice.

**Fig. 41:**
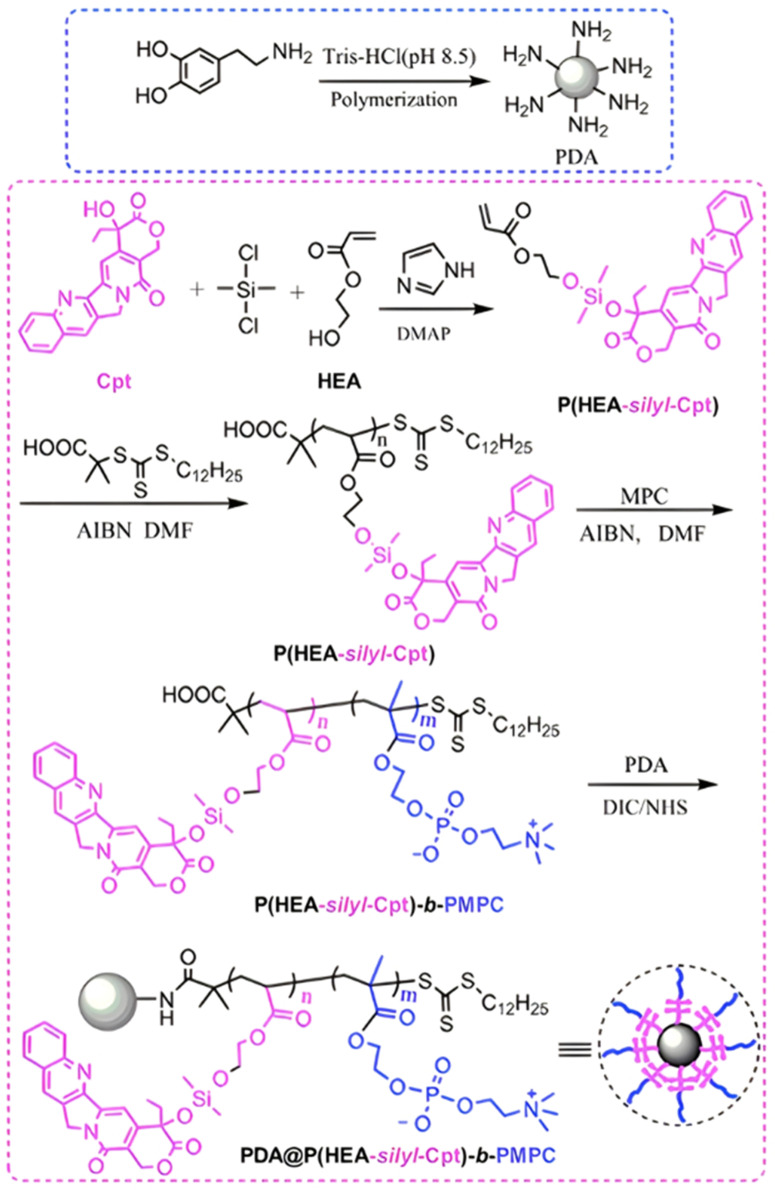
Synthetic route for pH-sensitive P(HEA-*silyl*-Cpt)-*b*-PMPC diblock copolymer prodrug and grafting onto PDA nanoparticles for combined pH- and light-sensitivities. Adapted from ref. 227.

Such a combination of stimuli has also been employed to design multi-responsive polymer prodrug nanocarriers based on Cpt.^227^ A silyl ester bond has been inserted between Cpt and HEA using chlorodimethylsilane prior to its RAFT polymerization and followed by chain-extension with MPC to achieve P(HEA-*silyl*-Cpt)-*b*-PMPC diblock copolymer prodrug (Fig. 41). Its grafting onto polydopamine (PDA) nanoparticles *via* amidation was then carried out without altering the photothermal properties of PDA. Significant silyl ether bond cleavage was observed at pH 5 compared to pH 7.4 (∼92 *vs.* 22% after 12 h, respectively). Such drug release in acidic conditions was further enhanced upon laser irradiation, up to 71% at pH 5 after 120 min (*vs.* 52% without laser), confirming a synergistic effect of pH-driven cleavage and photothermal therapy. Cytotoxicity studies on HeLa cells and *in vivo* experiments on tumor-bearing mice confirmed the beneficial effect of combining pH- and light-sensitivities for anticancer therapy.

**Fig. 42:**
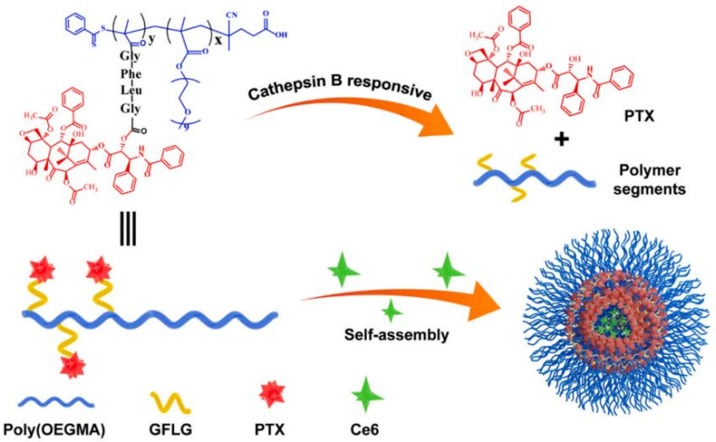
Structure of P(MAAm-GFLG-Ptx)-*b*-POEGMA polymer prodrugs and their degradation mediated by cathepsin B, leading to release of Ptx and Ce6. Adapted from ref. 228.

The combination of enzyme-sensitivity and the possibility of performing PDT using polymer prodrugs obtained by the “grafting through” method has also been reported. The enzyme-sensitive monomer prodrug based on Ptx, MAAm-GFLG-Ptx, was polymerized from a POEGMA macro-RAFT agent leading to 130 nm P(MAAm-GFLG-Ptx)-*b*-POEGMA polymer prodrug nanoparticles able to physically encapsulate Chlorin e6 as the photosensitizer for PDT (Fig. 42).^228^ The presence of cathepsin B accelerated the release of Ptx *in vitro*, reaching 80% after 8 h. In parallel, the loaded-photosensitizer was also quickly released, which was assigned to the disassembly of the nanoparticles. *In vivo* experiments on T24 bladder multicellular tumor spheroids and on T24 tumor-bearing mice showed significant antitumor efficacy due to the combination of Ptx chemotherapy and PDT.

## 5. Growing the polymer from the drug (grafting from)

**Fig. 43:**
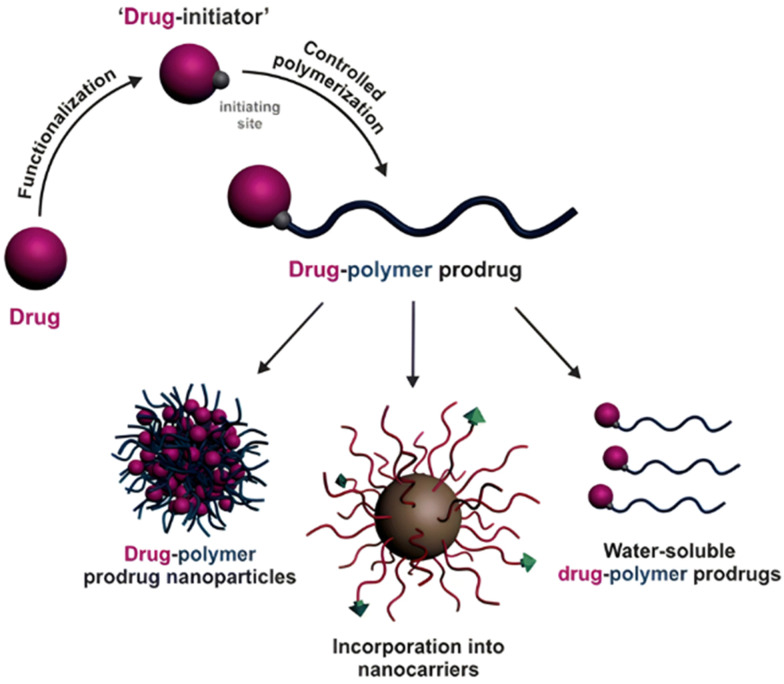
Design of polymer prodrugs by the “grafting from” or “drug-initiated” method and their use in drug delivery under the form of polymer prodrug nanoparticles, polymer prodrugs loaded into drug delivery systems or as water-soluble polymer prodrugs. Adapted from ref. 97.

To continue the analogy with polymer synthesis, the third synthetic strategy to design polymer prodrugs is termed the “grafting from” or “drug-initiated” method (Fig. 3c). It is perhaps the most simple and efficient route to construct polymer prodrugs by RDRP due to its simplicity, versatility and robustness,^97^ compared with the “grafting to” and “grafting through” approaches, which still possess some limitations (*e.g.*, multistep synthetic routes, significant workup, moderate yields, *etc.*). It relies on the growing by RDRP of a polymer chain from the drug previously functionalized by controlling agent (*e.g.*, alkoxyamine for NMP, ATRP initiator, RAFT agent), resulting in polymer prodrug consisting of one drug molecule linked at the chain-end of a well-defined polymer (Fig. 43). After polymerization, a simple purification step is required to remove the unreacted monomer which is often a volatile. In addition, due to the flexibility and tolerance of RDRP towards functional groups, the “grafting from” approach represents an efficient route to produce a wide range of polymer prodrugs with tunable properties (*e.g.*, soluble, amphiphilic, hydrophobic), thanks to the easy modulation of the nature of the drug, the linker and the polymer (Fig. 43).

Similarly to the “grafting to” and “grafting through” approaches, facile insertion of stimuli-sensitive linkers between the drug and the polymer chain has also been achieved with the “grafting from” approach, leading to a diversity of activable drug delivery systems either by endogenous stimuli (Table 5) alone or in combination with external stimuli such as the temperature (see Table 6, Section 4.2).

**Table 5:** Stimuli-sensitive drug linkers in polymer prodrugs obtained by RDRP techniques *via* the “grafting from” or “drug-initiated” strategy

Linker	Drug	Polymerization method	Nanocarrier	Cleavageconditions	Release	Ref.
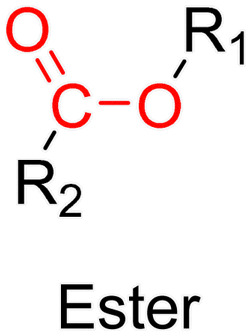	CdA (ester) (diglycolate)	NMP	PI	Human serum	Ester: <1% after 24 h	235
					Diglycolate: 20% after 24 h	
	Ptx (diglycolate)	NMP	PI	Human serum	3–5% after 24 h	237
	Ptx (diglycolate)	RAFT	POEGMA	Human serum	30% after 24 h	237
	CdA (ester) (diglycolate)	RAFT	PSqMA	Human serum	Ester: 25% after 24 h	236
					Diglycolate: 28% after 24 h	

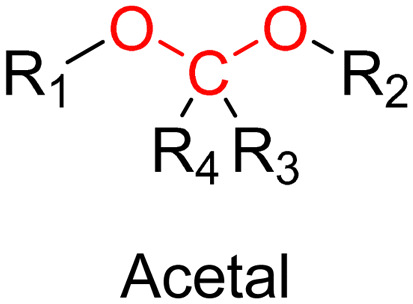	Ptx	RAFT (initiator + DHP) (initiator + DEGVE)	PDMA	pH 4	DHP: <10% after 96 h	234
					0% after 96 h	
				pH 5	DEGVE: 30% after 96 h	
					< 10% after 96 h	

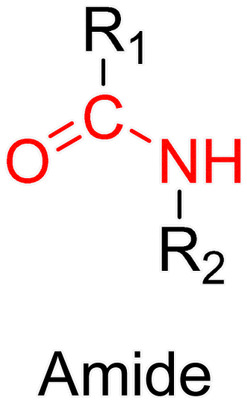	Gem	RAFT	Gem-PMMA	pH 5.5 + cathepsin B	71.6% after 72 h	231
	Gem (amide) (amide + diglycolate)	NMPrROP	Gem-P(MMA-*co*-MPDL)	Human serum	Amide: <2% after 24 h	238
					Amide/diglycolate: <7–13% after 24 h	
			Gem-P(OEGMA-*co*-MPDL)		Amide: <8–25% after 24 h	
	Gem	NMP	PI	Human serum	6.1% after 24 h	230
	Gem (amide) + Dox/Lap (succinate) (diglycolate)	NMP	PI	Human serum	Gem: <2% after 24 h	239
					Dox/Lap: no release	

**Table 6:** Dual-sensitive prodrugs based on the combination of endogenous stimuli-sensitive linkers and external stimuli obtained *via* the “drug-initiated” strategy

External stimuli	Drug	Drug linker	Polymerization method	Nanocarrier	Cleavage conditions	Release	Role of external stimuli	Ref.
Temperature	Gem	Amide	RAFT	PHEA-*b*-PHEAmTHP	Human serum	∼10% after 168 h	Thermo-driven all-aqueous formulation	242

### 5.1. The use of endogenous stimuli

#### 5.1.1. pH-Sensitivity

Similarly to polymer prodrugs obtained by the two previous strategies, pH-sensitivity has also been implemented into polymer prodrugs from the “grafting from” pathway *via* the easy insertion of pH-sensitive linkers between the drug-controlling agent and the growing polymer chain.

##### 5.1.1.1. Amide linker

**Fig. 44:**
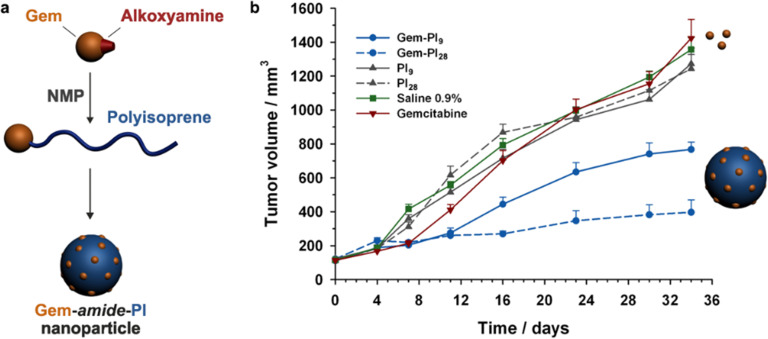
(a) Schematic representation of the synthesis of Gem-*amide*-PI polymer prodrugs by NMP and their self-assembly into nanoparticles; (b) evolutions of tumor volume with time following intravenous injection (on days 0, 4, 8 and 12) of Gem (–▼–, 7 mg kg^−1^), Gem-*amide*-PI nanoparticles [–●– (Gem-PI9) and --●-- (Gem-PI_28_), 7 mg kg^−1^ Gem-equivalent dose], control (–■–, saline 0.9%) and PI nanoparticles [–▲–, (PI_9_) and –▲--, (PI_28_), same dose of polymer as Gem-PI]. Adapted from ref. 230.

The amide bond is suitable for enzymatic cleavage (*e.g.*, proteases), but also for non-enzymatic cleavage following either direct hydrolysis or intramolecular aminolysis (Fig. 1).^229^ A typical example is the coupling of Gem through its amine group to: (i) the NMP alkoxyamine initiator 2-(*N-tert*-butyl-*N*-(1-diethoxyphosphoryl-2,2-dimethylpropyl)aminoxy)propionic acid) (AMA-SG1) using PyBOP as a coupling agent prior to the polymerization of isoprene(i) to produce Gem-*amide*-PI polymer prodrugs (Fig. 44a)^230^ or (ii) *S*-1-dodecyl-*S*′-(α,α′-dimethyl-α′′-acetic acid)trithiocarbonate as a RAFT agent prior to the polymerization of the methyl methacrylate (MMA) to achieve Gem-*amide*-PMMA polymer prodrugs.^231^ In both cases, Gem-*amide*-PI and Gem-*amide*-PMMA polymer prodrugs exhibited high drug loadings (the lower the *M*_n_, the higher the drug loading) in the 10–30 wt% range and ability to self-assemble into 120–160 nm nanoparticles by nanoprecipitation without the use of a surfactant. Enhanced Gem release at pH 5.5 from Gem-*amide*-PMMA has been observed after 72 h reaching ∼47% compared to only 10% at physiological pH. Interestingly, further enhancement of the Gem release by a factor 1.5 (reaching ∼72%) was observed at pH 5.5 in presence of cathepsin B. *In vitro* assays on a small library of cancer cell lines (*i.e.*, MiaPaCa-2, L1210, CCRF-CEM, A549 and MCF-7) demonstrated significant cytotoxicity of both types of polymer prodrug nanoparticles (Fig. 44b). *In vivo* studies on MiaPaCa-2 and A549 tumor-bearing mice concluded to a significant anticancer efficacy and reduced side effects compared to free Gem.

##### 5.1.1.2. Acetal linker

**Fig. 45:**
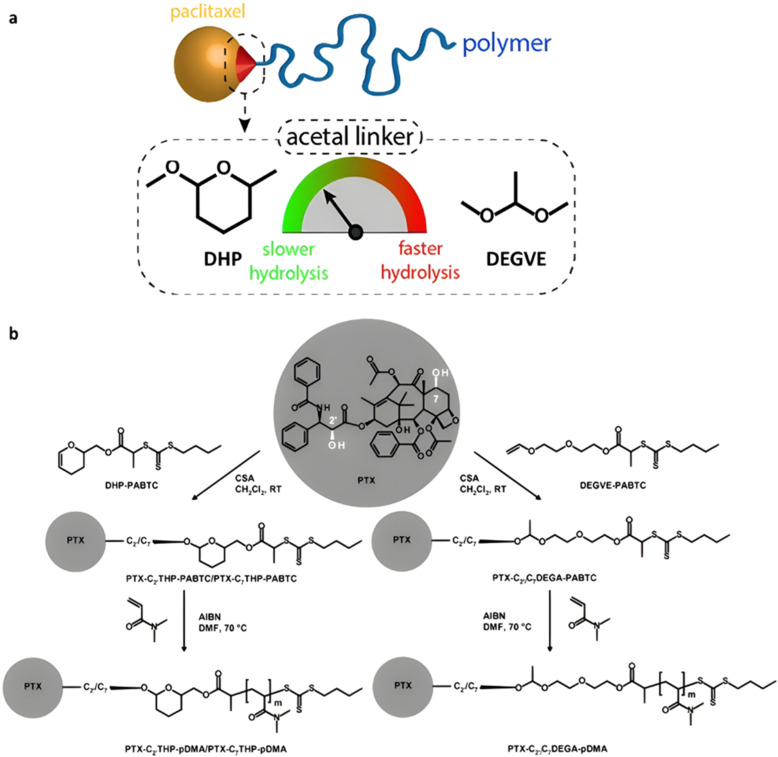
(a) Schematic representation of hydrolysis kinetics using linear (DEGVE) and cyclic (DHP) acetal linkers in Ptx-*acetal*-PDMA prodrugs; (b) acid-catalyzed acetalization of Ptx with a dihydropyran (DHP) or a di(ethylene glycol) vinyl ether (DEGVE) derivative of PABTC. Adapted from ref. 234.

The acetal linker has been used to design stimuli-sensitive drug delivery systems due to its sensitivity to slightly acidic pH and its ease of formation, requiring only alcohol and ketone/aldehyde functional groups (Fig. 1).^232^ Based on previous work on the synthesis of Ptx-*ester*-poly(*N*,*N*-dimethylacrylamide) (Ptx-*ester*-PDMA) by the “drug-initiated” method,^233^ conjugation of Ptx *via* its C2′ or C7 hydroxyl groups to (2-(butylthiocarbonothioylthio)propanoic acid (PABTC) as a RAFT agent was carried out. It was equipped with either a dihydropyran (DHP) or a di(ethylene glycol)vinyl ether (DEGVE) moiety, leading to a cyclic or linear acetal bond, respectively (Fig. 45).^234^ Both Ptx-based RAFT agents were used to control the polymerization of DMA resulting in Ptx-*acetal*-PDMA polymer prodrugs. Both families of polymer prodrugs formed micelles of 15 nm in diameter upon self-assembly, which remained stable at pH 7.4 without early release of Ptx. Interestingly, acidic conditions (pH 5) resulted in the release of 6% Ptx after 4 days for the linear acetal linker, whereas no release has been observed for the cyclic counterpart, probably due to its higher chemical stability. Stronger acid conditions (pH 4) further enhanced the release with similar trend (30% *vs.* <5% after 4 days, respectively). The specificity of pH-mediated Ptx release was also demonstrated by the absence of release in the presence of fetal bovine serum for both types of acetal linkers. *In vitro* evaluations on SKOV-3 cells confirmed the drug release profiles, as linear acetal linker induced greater cytotoxicity than the cyclic counterpart, with IC_50_ values of 0.51 μM and 95 μM, respectively.

#### 5.1.2. Enzymatic-sensitivity

Enzyme sensitivity is another feature that can be easily conferred on RDRP-derived polymer prodrugs obtained by the “grafting from” approach.

##### 5.1.2.1. Ester linker

**Fig. 46:**
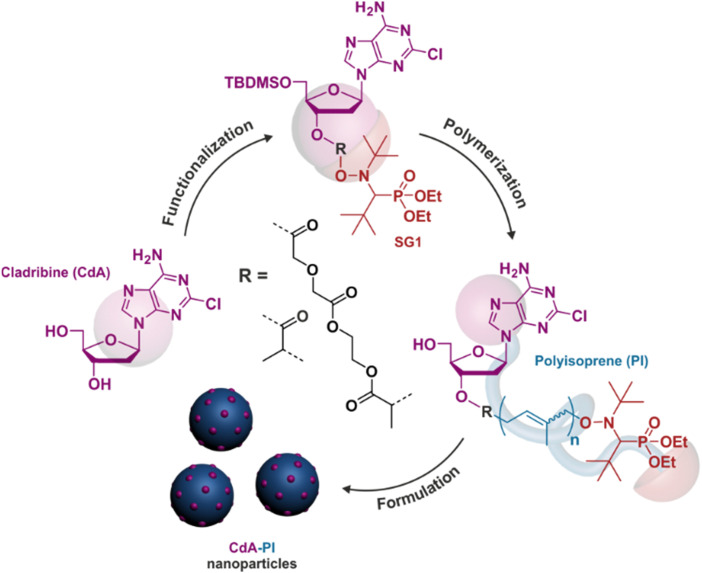
“Drug-initiated” NMP of isoprene using CdA-based initiator and formulation into polymer prodrug nanoparticles. Adapted from ref. 235.

Ester groups in polymer prodrugs deriving from the “drug-initiated” method can be efficiently cleaved by enzymes providing they are accessible and solvated enough. This is what has been shown by a series of studies aiming to establish a structure–drug release–cytotoxicity relationships. One of them reported the synthesis of polymer prodrugs based on cladribine (CdA), an anticancer agent used in the treatment of some leukemias.^235^ CdA was conjugated to AMA-SG1 through either a methyl-substituted ester bond (CdA-*ester*-AMA-SG1) or a diglycolate bond (CdA-*digly*-AMA-SG1), which is known to be more hydrophilic and labile. Both drug-alkoxyamines were used to polymerize isoprene by NMP, producing CdA-*ester*-PI and CdA-*digly*-PI polymer prodrugs, respectively (Fig. 46). The nanoparticles obtained by nanoprecipitation exhibited a diameter in the 110–160 nm range, with no release of CdA in human serum from CdA-*ester*-PI (<1% after 24 h) conversely to 20% from CdA-*digly*-PI. This result was assigned to the difference in steric hindrance, but also to a greater solvation and lability of the diglycolate linker. Interestingly, no obvious difference of release in PBS was observed (<1 and <3%, respectively, after 24 h), confirming the enzymatic-driven cleavage of the ester groups. In line with the release experiments, CdA-*digly*-PI nanoparticles showed significant cytotoxicity on L1210 cells, whereas CdA-*ester*-PI nanoparticles did not induce any cell death.

**Fig. 47:**
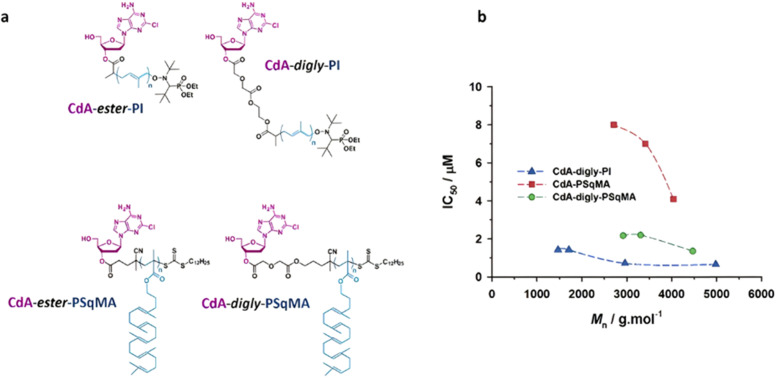
(a) Structure of CdA-*digly*-PI, CdA-*digly*-PSqMA, CdA-*ester*-PSqMA (linear) and CdA-*ester*-PI (methyl-substituted) polymer prodrugs; (b) evolution of the IC_50_ as a function of the nature and of the *M*_n_ of the CdA-based polymer prodrugs. Note that CdA-*ester*-PI (methyl-substituted) is not represented because the IC_50_ was never reached due to the absence of CdA release. Adapted from ref. 236.

Analogous polymer prodrugs obtained by replacing isoprene by squalene methacrylate (SqMA) and exhibiting either a linear ester or a diglycolate linker have also been reported (Fig. 47a). It was shown that CdA-*digly*-PSqMA and CdA-*ester*-PSqMA nanoparticles resulted in the significant and comparable release of CdA in human serum (25–28% after 24 h). This is explained by the hydrophilic nature of CdA, which promotes solvation and cleavage of the ester bonds. Moreover, no release was shown in PBS, which supported the specificity of the enzymatic cleavage of the diglycolate bond.^236^ Although no difference in terms of drug release was shown, CdA-*digly*-PSqMA nanoparticles exhibited the highest cytotoxicity on L1210 cancer cells compared to the linear ester counterpart.

Taking all these results about CdA-based polymer prodrugs together, a cytotoxicity ranking was established, reflecting the importance of the nature of the linker (*e.g.*, solvation, steric hindrance), but also the influence of the steric hindrance provided by the polymer (linear for PI *vs.* comb-like for PSqMA): CdA-*digly*-PI > CdA-*digly*-PSqMA > CdA-*ester*-PSqMA (linear) ≫ CdA-*ester*-PI (methyl-substituted). It was also shown that the *M*_n_ of the polymer employed can also play a role in the cytotoxicity, especially for moderately active polymer prodrugs (Fig. 47b).

**Fig. 48:**
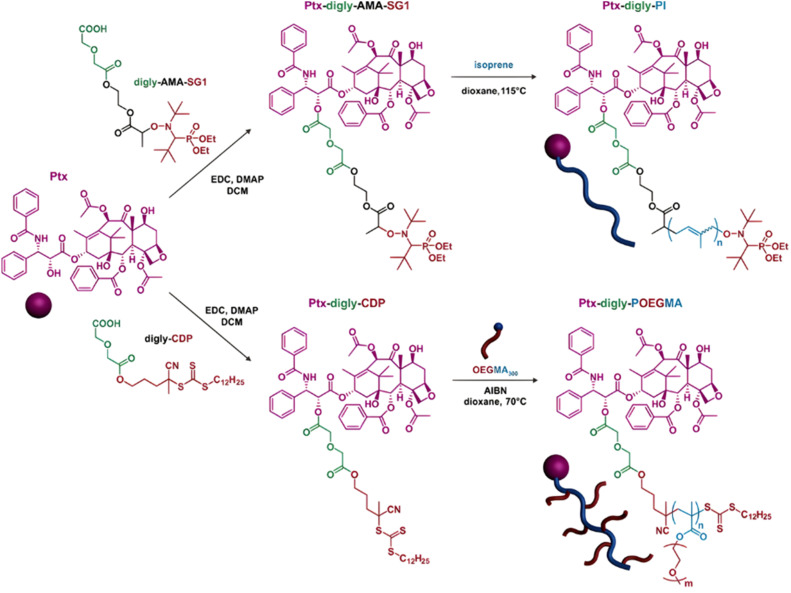
Synthesis of Ptx-*digly*-PI and PEGylated Ptx-*digly*-POEGMA polymer prodrugs by NMP. Adapted from ref. 237.

As expected, when developing similar polymer prodrugs with a strongly hydrophobic drug such as Ptx, by polymerizing isoprene by NMP from a Ptx-*digly*-AMA-SG1 initiator (Fig. 48), the resulting Ptx-*digly*-PI nanoparticles led to much lower release of Ptx in human serum compared to CdA-*digly*-PI counterparts (3–5% *vs.* 20%, respectively, after 24 h).^237^ This result supported the detrimental effect of a hydrophobic environment around the ester group, preventing efficient enzymatic cleavage. Interestingly, this could be improved by growing a hydrophilic polymer such as POEGMA from Ptx (Fig. 48). The Ptx-*digly*-POEGMA polymer prodrugs were able to self-assemble into nanoparticles of 133–151 nm in diameter, which led to 32% release of Ptx in human serum after 24 h. This showed that the detrimental effect of using hydrophobic drugs can be compensated by the use of hydrophilic polymers when constructing the polymer prodrugs by the “drug-initiated“ method. Importantly, the released Ptx in PBS reached 14% after 24 h, which is higher than any PI-based prodrugs.

##### 5.1.2.2. Amide linker

**Fig. 49:**
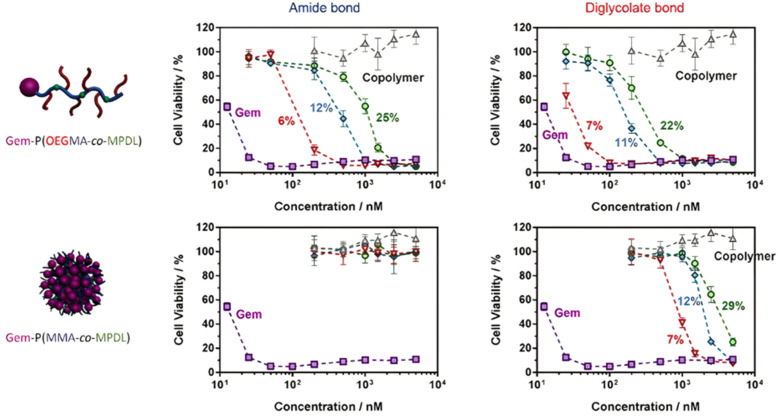
Cell viability (MTT test) with increasing concentrations of Gem, Gem-*amide*-P(MMA-*co*-MPDL), Gem-*amide-digly*-P(MMA-*co*-MPDL), Gem-*amide*-P(OEGMA-*co*-MPDL), Gem-*amide-digly*-P(OEGMA-*co*-MPDL) and P(MMA-*co*-MPDL) on MiaPaCa-2 cells. Adapted from ref. 238.

Like ester groups, the susceptibility to enzymatic cleavage of amide linkers in “drug-initiated” polymer prodrugs is strongly influenced by solvation and steric hindrance in the vicinity of the amide functionality. This was illustrated by a study of the relationship between polymer prodrug structure, drug release and cytotoxicity on Gem-based polymer prodrugs, whose amide linker had different environments affecting its cleavage.^238^ More specifically, by using the corresponding alkoxyamine initiator, four different families of polymer prodrugs were synthesized by copolymerization of a traditional vinyl monomer (MMA or OEGMA) and 2-methylene-4-phenyl-1,3-dioxolane (MPDL), a cyclic ketene acetal (CKA) monomer precursor of ester group in the main chain: (i) Gem-*amide*-P(MMA-*co*-MPDL); (ii) Gem-*amide-digly*-P(MMA-*co*-MPDL); (iii) Gem-*amide*-P(OEGMA-*co*-MPDL) and (iv) Gem-*amide-digly*-P(OEGMA-*co*-MPDL). For each family of polymer prodrugs, three different MPDL contents were investigated: 7, 11 and 24 mol%. Hydrophobic polymer prodrugs based on MMA formed 109–196 nm nanoparticles by nanoprecipitation, while the use of OEGMA as the main vinyl monomer led to hydrophilic polymer prodrugs. The release of Gem was studied in human serum for 24 h and resulted in the following trend in terms of drug release efficiency: Gem-*amide*-P(MMA-*co*-MPDL) with less than 2% release < Gem-*amide-digly*-P(MMA-*co*-MPDL) with 7–13% release < Gem-*amide*-P(OEGMA-*co*-MPDL) with 8–25% release < Gem-*amide-digly*-P(OEGMA-*co*-MPDL) with 18–70% release. Remarkably, such ranking directly correlated with the cytotoxicity of the polymer prodrugs on both MiaPaCa-2 and A549 cells (Fig. 49). Overall, the anticancer activity was independently governed by three structural parameters: (i) soluble OEGMA-based prodrugs were more cytotoxic than MMA-based counterparts due to a greater solvation of the linker; (ii) the lower the MPDL content, the greater the anticancer activity due to a decrease in hydrophobic monomer units and (iii) a diglycolate moiety afforded greater cytotoxicity compared to a simple amide bond due to its greater hydrophilicity and lability.

**Fig. 50:**
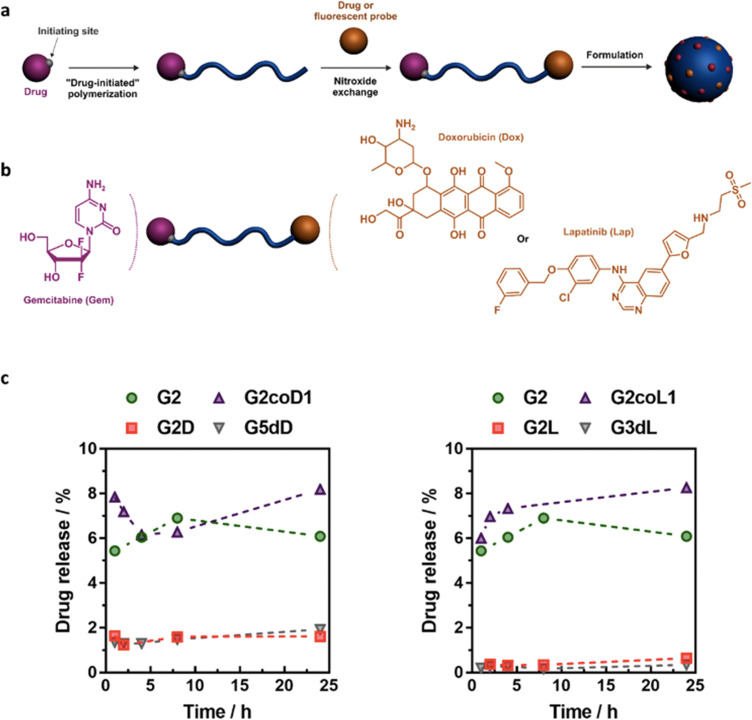
(a) Synthesis of heterobifunctional polymer prodrugs by “drug-initiated” synthesis of polymer prodrugs followed by the nitroxide exchange reaction using a functional nitroxide; (b) heterotelechelic polymer prodrug bearing either Gem/Dox or Gem/Lap combination; (c) Gem release profiles at 37 °C in human serum from: Gem-*amide*-PI (G2), Gem-*amide*-PI-*amide*-Dox (G2D), Gem-*amide*-PI-*amide-digly*-Dox (G5dD), Gem-*amide*-PI-*amide*-Lap (G2L) and Gem-*amide*-PI-*amide-digly*-Lap (G3dL) nanoparticles, and from nanoparticles obtained by the co-nanoprecipitation of Gem-*amide*-PI and PI-*amide*-Dox (G2coD1) or PI-*amide*-Lap (G2coL1) prodrugs. Adapted from ref. 100.

In relation to previous results, the supramolecular organization of polymer prodrug nanoparticles derived from the “drug-initiated” method can also greatly affect the drug release kinetics. This has been shown with heterotelechelic polymer prodrugs in which the chain end of Gem-*amide*-PI prodrugs has been functionalized with another anticancer drug such as Dox or Lapatinib (Lap), for combination therapy purposes.^100^ This was achieved by a general post-functionalization approach *via* the nitroxide exchange reaction using 2,2,6,6-tetramethylpiperidin-1-yl)oxy (TEMPO) nitroxide previously functionalized by the drug of interest (Fig. 50a), which can also be adapted to targeting ligands,^239^ as well as fluorescent dyes for *in vitro* and *in vivo* imaging.^240^ While Gem was linked to PI through an amide linker, Dox and Lap were conjugated through either an *amide* or an amide-diglycolate linker. Resulting heterobifunctional prodrugs all formed nanoparticles (Fig. 50b), with diameters in the 99–142 nm range but exhibited different Gem release kinetics in human serum depending on the polymer prodrug structure. While the release of Gem from Gem-*amide*-PI nanoparticles reached 6% after 24 h, it was considerably lowered (0.5–2%) with all heterobifunctional polymer prodrug nanoparticles (Fig. 50c). Interestingly, co-nanoprecipitation of monofunctional polymer prodrugs (*i.e.*, Gem-*amide*-PI and PI-*amide*-Dox) resulted in Gem release similar to that of Gem-amide-PI nanoparticles (∼8%). These results suggested that dual functionalization of the same polymer chain by different drugs induced a change in the supramolecular organization of nanoparticles, therefore affecting drug localization and ultimately access by enzymes. Furthermore, neither Dox nor Lap could be detected during release study, regardless of the nature of their linker, which can be explained by their strong hydrophobicity, hiding them into the core of the nanoparticles, combined with the too high colloidal stability of PI-based polymer prodrugs during *in vitro* experiments. The influence of dual functionalization has also been demonstrated on cytotoxicity as Gem-*amide*-PI-*amide*-Dox nanoparticles did not show any improvement over monofunctional polymer prodrug nanoparticles, but co-nanoprecipitation of Gem-amide-PI/PI-amide-Lap polymer prodrugs led to synergistic effect on MCF-7 cells.

**Fig. 51:**
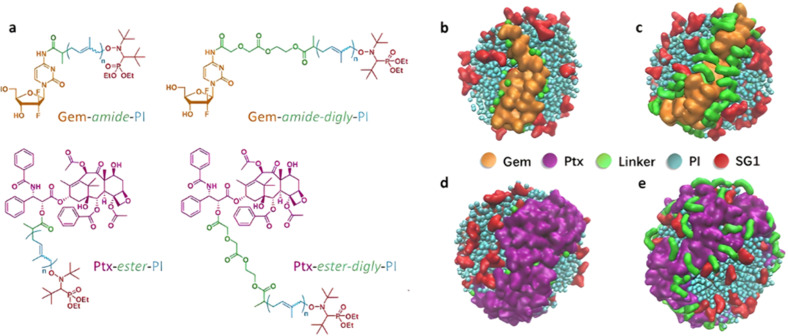
(a) Chemical structures of Gem-*amide*-PI, Gem-*amide-digly*-PI, Ptx-*ester*-PI and Ptx-*ester-digly*-PI polymer prodrugs. Illustration of the Gem (orange beads), Ptx (purple beads), linker (green beads) packing at the nanoparticle periphery compared to the SG1 nitroxide groups (red beads) and isoprene units (blue beads) for (b) Gem-*amide*-PI, (c) Gem-*amide-digly*-PI, (d) Ptx-*ester*-PI and (e) Ptx-*ester-digly*-PI. Adapted from ref. 241.

To better anticipate the efficacy of drug release and hence the cytotoxicity of polymer prodrugs obtained by this synthetic strategy, a coarse-grained molecular dynamics simulation study based on the MARTINI 2 force field was carried out on four representative polymer prodrugs, two with Gem (Gem-*amide*-PI and Gem-*amide-digly*-PI) and two with Ptx (Ptx-*ester*-PI and Ptx-*ester-digly*-PI), to gain insight into their supramolecular organization and in particular le localization of the drug and the drug–polymer linker (Fig. 51).^241^ It was shown that the drug–polymer linkers (green beads) were not fully accessible to solvent, probably due to drug aggregation and/or partial burying in the nanoparticle core. More precisely, among the three possible cleavage sites of the *digly* linker, the one close to the drug was poorly solvated, similarly to the unique cleavage site of the amide/ester linker. Its two other cleavage sites, that include a labile ester group, were significantly more accessible to the solvent. These simulations might account for the differences observed in drug release experiments between the four polymer prodrug structures.

### 5.2. The use of exogenous stimuli

It has been previously shown that the combination of stimuli-sensitive drug linkers with external stimuli could help trigger and/or refine the drug release. The temperature remains an easy stimulus to implement as polymers can be thermo-sensitive with a LCST or an upper critical solution temperature (UCST).

**Fig. 52:**
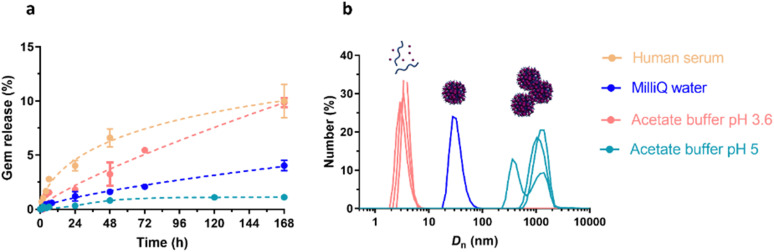
(a) Gem release profiles (HPLC) of Gem-PHEA-*b*-PHEAmTHP prodrug nanoparticles in MilliQ water, human serum, and acid buffers; (b) DLS evaluation of the number-average diameter (*D*_n_) of associated (nano-)objects. Adapted from ref. 242.

In this context, Gem has been conjugated to the RAFT agent PABTC *via* an amide bond prior to the copolymerization of HEA with hydroxyethylacrylamide tetrahydropyran (HEAmTHP).^242^ Resulting Gem-*amide*-PHEA-*b*-PHEAmTHP diblock copolymer prodrugs were sensitive to three stimuli (Table 6): (i) the presence of proteases due to the amide linker, (ii) the acid pH due to the acetal bond inserted between the THP moiety and HEAm and (iii) the temperature as PHEAmTHP exhibited a LCST behavior. A copolymer with a Gem-PHEA : HEAmTHP molar ratio of 1 : 30 led to the formation of 50 nm-nanoparticles by an all water nanoprecipitation process, which consists of solubilizing the copolymer in water at a temperature below its transition temperature (8 °C), then adding the copolymer solution to water at a temperature above its transition temperature. Only a modest release of Gem (<5% after 168 h) was obtained in water and in slightly acidic medium (pH 5). However, such prodrug nanoparticles exhibited significantly higher Gem release at lower pH (3.6) and in human serum, leading to a 2-fold faster release (10% after 168 h) in both media (Fig. 52a). Faster Gem release in human serum is likely assigned to the presence of specific enzymes able to cleave the amide bond between Gem and the copolymer. The significant difference in Gem release at pH 5 and 3.6 lies in the fact that only partial acetal hydrolysis may be achieved at pH 5 whereas it may be complete at pH 3.6 (the rate of acetal hydrolysis is indeed strongly accelerated when decreasing pH, following a first-order kinetics^243,244^). These behavioral differences were confirmed by DLS showing aggregation of nanoparticles in acidic buffers at pH 3.6 and pH 5 (*D*_n_ = 3 900 and 2 900 nm, respectively), followed by their dissolution after 168 h at pH 3.6 (*D*_n_ = 4 nm), while larger objects could still be observed at pH 5 (*D*_n_ = 1 500 nm) (Fig. 52b) that may trap the drug. *In vitro* studies on A549 and Mia PaCa-2 cell lines demonstrated fast cell internalization after 4 h and significant cytotoxicity even if the IC_50_ remained slightly higher than free drug.

## 6. Conclusion

In this review article, we have covered the different synthetic strategies to achieve polymer prodrug nanocarriers by RDRP techniques that are sensitive to endogenous in combination or not with exogenous stimuli.

The ease of synthesis of polymer prodrugs *via* the “grafting to” approach has facilitated the insertion of various chemical bonds between the drug and the polymer, and also between polymer blocks, aiming to design multi-responsive drug delivery systems. Additional sensitivity to exogenous stimuli has also been conferred to enhance the drug delivery efficacy and ultimately the therapeutic effect. However, this synthetic approach led to relatively low drug loadings due to steric hindrance problems that disadvantaged drug conjugation and also necessitated multi-step synthetic procedures and extensive workup. The “grafting through” approach has enabled the design of various polymer prodrug nanocarriers with higher drug loadings due to less steric hindrance when polymerizing prodrug monomers instead of grafting drug onto preformed polymers. A diversity of drug–polymer linkers has been incorporated between the monomer and the drug, and within the monomer, each responding to endogenous stimuli and enabling a better control of the drug release kinetics. The combination of different stimuli can help trigger the drug release and enhance the therapeutic efficacy. This approach also enabled the implementation of two different endogenous stimuli using stimuli-responsive drug linkers and polymers. It has also been possible to combine endogenous stimuli to external sensitivities to exert additional therapeutic modalities such as hyperthermia, PDT or PTT therapies. The simplicity, robustness and versatility of the recently developed “drug-initiated”/“grafting from” method has made it possible to design polymer prodrugs based on different drugs and with adjustable properties, enabling either nanoparticles/micelles or water-soluble polymer prodrugs to be obtained. The added value of RDRP regarding the “grafting from“ approach is the possibility of adjusting drug loading notably by varying the polymer chain length, as well as chain-end functionalization to insert other molecules of interest (*e.g.*, second drug, targeting ligand, fluorescent probe).

It should be noted that, as with every class of drug delivery system, and in view of the many new systems regularly reported in the literature, the need for comparative studies appears essential, given their essential role in identifying the most promising candidates for clinical application.

## 7. Lessons learned from antibody–drug conjugates (ADCs)

Antibody–drug conjugates (ADCs) have shown success in targeted therapies by leveraging the specificity of antibodies to deliver cytotoxic payloads directly to cancer cells.^245^ The success of ADCs in targeted cancer therapy may offer valuable insights for the development of next-generation polymer prodrug nanocarriers.

When using RDRP techniques, polymer prodrug nanocarriers can easily benefit from similar strategies by incorporating targeting moieties into their structure. For instance, RDRP polymers car be functionalized *via* post-polymerization at the chain end with small targeting ligands such as vitamins, either by using controlling agents bearing functional handles^246^ to enable coupling with the targeting moiety, or by performing chain-end modification to directly install the targeting moiety.^240^ Alternatively, targeted polymer prodrugs car be obtained in one step by the “drug-initiated*”* method *via* the use of pre-functionalized controlling agents with the targeting ligand.^247^ Future developments could involve applying this strategy to much more effective ligands such as proteins,^248^ aptamers or nanobodies/antibody fragments. This approach could significantly improve drug delivery to diseased tissues and reduce potential side effects on healthy cells.

The linker chemistry between the antibody and drug in ADCs plays a crucial role in achieving controlled drug release at the target site.^249^ This ensures that the cytotoxic payload is released only after reaching the cancer cells, minimizing systemic exposure. Polymer prodrug nanocarrier development can benefit from similar linker strategies using the robustness of RDRP techniques and their compatibility with a wide range of functional groups. By incorporating carefully designed cleavable linkers responsive to specific stimuli like enzymes or changes in pH, one can design polymer prodrug nanocarriers that release the drug mainly upon encountering these triggers at the target site.

Similar to ADCs, which are often designed to be biocompatible and ultimately degraded by the body,^245^ polymer prodrug nanocarriers also need to prioritize these aspects for safe and effective drug delivery. RDRP techniques allow for the synthesis of biocompatible^105^ and (bio)degradable^39^ polymers to ensure the safety and degradability of polymer prodrug nanocarriers. This guarantees that the polymer carrier itself does not cause toxicity and is eventually degraded and fully excreted by the body after the drug has delivered its therapeutic effect.

The success of ADCs also relies on careful selection of both the antibody and the cytotoxic drug to ensure optimal targeting and therapeutic effect.^245^ Similarly, for polymer prodrug nanocarriers, the choice of the polymer, drug, and linker needs to be carefully considered and optimized based on the specific therapeutic target.

By incorporating these lessons learned from the well-established field of ADCs, one can design even more effective for polymer prodrug nanocarriers with targeted delivery, controlled release, and enhanced biocompatibility, leading to the next generation of drug delivery systems.

## Conflicts of interest

There are no conflicts to declare.
